# Redox‐Switchable Poly‐Lewis Acids Allow the Controlled Release of Guests

**DOI:** 10.1002/anie.202509191

**Published:** 2025-08-12

**Authors:** Maximilian J. Klingsiek, Yury V. Vishnevskiy, Julian Buth, Jan‐Hendrik Lamm, Beate Neumann, Hans‐Georg Stammler, Norbert W. Mitzel

**Affiliations:** ^1^ Lehrstuhl für Anorganische Chemie und Strukturchemie (ACS), Fakultät für Chemie Universität Bielefeld Universitätsstraße 25 D‐33615 Bielefeld Germany

**Keywords:** Controlled release, Coordination polymers, Host–guest chemistry, Poly‐Lewis acids, Redox switching

## Abstract

Presently, there is a notable interest in poly‐Lewis acids (PLAs), their host‐guest chemistry and their application in catalytic processes. The present study combines PLAs with redox‐active dibenzo[*a*,*e*]cyclooctatetraene (dbCOT) units. dbCOT‐based PLAs can be planarised by a two‐electron reduction. The molecular structures of the reduced PLAs, studied by sc‐XRD experiments and DFT calculations, show the formation of extended π‐systems. This π‐system extends to the Lewis acid functions and reduces their acidity. This allows a controlled release of complexed guest molecules. Oxidation regenerates the original Lewis acidity. Such reduction/oxidation cycles were also applied to an Al─P coordination polymer. sc‐XRD shows the connectivity of the coordination polymer to remain unchanged, so that the polymer is stable in the neutral and reduced state. The possibility of switching such dbCOT‐based PLAs and their adducts as well as the formed coordination polymers between two states with different structures and electronic compositions is of interest for the development of functional materials in the fields of electronics, optics and magnetism as well as for the development of switchable catalysts.

## Graphical abstract

This study introduces redox‐switchable poly‐Lewis acids (PLAs) based on dibenzo[*a*,*e*]cyclooctatetraene, whose reduction leads to planarisation, π‐system extension, and weakened Lewis acidity, enabling controlled guest release. The reversible switching of these PLAs and their coordination polymers highlights their potential for applications in catalysis, electronics, and functional materials.

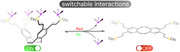

## Introduction

Poly‐Lewis acids (PLAs) are compounds that typically comprise a donor‐free organic backbone carrying two or more Lewis acid functions. The Lewis acid functions are either directly attached to the mostly aromatic backbone or joined to it via linkers such as alkyne or alkene units.^[^ ^1^, ^2^, ^3^, ^4^, ^5^, ^6^, ^7^, ^8^, ^9^, ^10^, ^11^, ^12^ ^]^ The Lewis acid functions of such PLAs can be based on a variety of elements, including silicon,^[^ ^13^, ^14^, ^15^ ^]^ tin,^[^ ^16^ ^]^ antimony,^[^ ^17^, ^18^, ^19^ ^]^ or mercury.^[^ ^20^, ^21^ ^]^ However, Group 13 elements (B, Al, Ga, and In) are the most commonly used due to their vacant p‐orbital and thus inherent electron deficiency, which eliminates the need for electron‐withdrawing substituents to induce relevant Lewis acidities.^[^ ^2^, ^3^, ^4^, ^5^, ^6^, ^8^, ^10^, ^12^, ^22^, ^23^, ^24^, ^25^, ^26^, ^27^, ^28^ ^]^ In recent years, there has been a significant increase in the investigation of bidentate^[^ ^13^, ^18^, ^23^, ^24^, ^29^, ^30^, ^31^ ^]^ PLAs; however, tri‐,^[^ ^11^, ^12^, ^14^, ^30^ ^]^ tetra‐,^[^ ^2^, ^19^, ^32^ ^]^ penta‐,^[^ ^33^ ^]^ hexa‐,^[^ ^12^, ^27^, ^34^ ^]^ and octadentate^[^ ^1^ ^]^ systems have also been reported. A novel backbone for tetradentate PLAs is dibenzocyclooctatetraene (dbCOT), which can be linked to Lewis acid functions with vinyl and ethynyl spacers. Such PLAs have been synthesised and a substantial range of host‐guest complexes have been generated and characterised, demonstrating their general reactivity.^[^ ^2^ ^]^

dbCOT is a non‐aromatic 16 π‐electron system that assumes a tub shape in equilibrium. In solution, dbCOT undergoes inversion by overcoming an energy barrier of 51 kJ mol^−1^.^[^ ^35^, ^36^ ^]^ In 1965, Katz succeeded in converting dbCOT to a planar Hückel‐aromatic 18 π‐electron system by means of a two‐electron reduction with lithium.^[^ ^37^ ^]^

In 2022, Petrukhina *et al.* determined the molecular structure of dbCOT^2−^ with Li^+^−Cs^+^ as counterions.^[^ ^38^ ^]^ In these molecular structures, the alkali element cations are bonded in varying hapticity to the cyclooctatetraene core. The molecular structure of the “naked” dianion was determined by complexing the potassium ion with [2.2.2]cryptand. The structures confirm the planarisation of dbCOT and the increased aromaticity of the COT core, while the benzene units become more diene‐like.^[^ ^38^ ^]^ In subsequent studies, Strand and co‐workers constructed electro‐mechanically switchable hydrocarbons by linking up to five dbCOT units via ethinyl spacers. They can be successively reduced electrochemically or with potassium and give several defined states that they deconvoluted by spectroscopy, but molecular structures were not determined yet. These systems provide an interesting platform for new responsive materials and switches.^[^ ^39^ ^]^ In 2021, the synthesis of dbCOT‐functionalised polymers was reported, which have the potential to serve as a material for battery electrodes.^[^ ^40^ ^]^

dbCOT thus represents a redox‐switchable unit, while the best known and most extensively studied redox‐switchable compound is ferrocene, and its derivatives find application in sensor technology, catalysis, medicine, and as functional materials.^[^ ^41^ ^]^ For example, polyferrocenes such as poly(vinylferrocene) are suitable as cathode materials for lithium‐ion batteries.^[^ ^42^ ^]^ Transition metal free redox‐switchable compounds have also been the focus of extensive research, with notable examples including the quaternary 4,4′‐bipyridinium salts (viologens) that have found application in electrochromic materials.^[^ ^43^, ^44^ ^]^ In addition, Davis and Beer have developed systems with redox‐switchable chalcogen bonds using viologen or ferrocene units as redox‐active units. Depending on the oxidation state, these systems can interact with anions via chalcogen bonding or release them.^[^ ^45^ ^]^

Due to the significant potential applications of dbCOT‐containing compounds, it is obvious to investigate the reducibility of dbCOT‐based PLAs and their reactivities and electronic properties dependent on the redox state. In addition, redox‐switchable (polydentate) host systems with classical Lewis acid functions have been less studied to date.^[^ ^46^ ^]^

## Results and Discussion

### Two‐Fold Reduction

**Figure 1:**
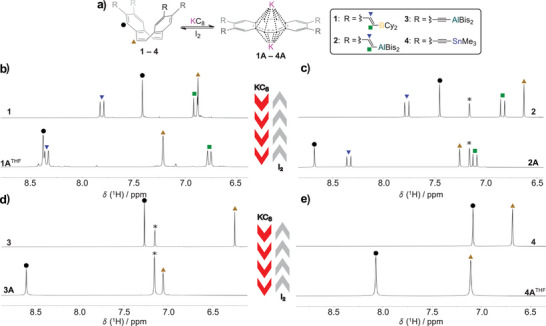
a) shows the general reduction of PLAs **1**–**4** using KC_8_, as well as the oxidation with iodine, illustrating the structural change of the PLAs associated with this redox reaction, where Bis stands for ‐CH(SiMe_3_)_2_ and Cy for ─C_6_H_11_; b)–e), show the higher field of the ^1^H NMR spectra of the neutral PLAs **1**–**4** and the reduced dications **1A**–**4A** which can be converted into each other by means of redox reactions; in b) and e) THF‐*d*_8_ was used as solvent in c) and d) C_6_D_6_ (*) was used to prevent adduct formation between the THF and the PLAs; for more details, see Supporting Information.

Previously, we established the synthesis of poly‐Lewis acids (PLAs) based on the *sym*‐dibenzo[*a*,*e*]cyclooctatetraene (dbCOT) and investigated their reactivity towards a broad range of neutral (multidentate) Lewis bases.^[^ ^2^ ^]^ As the PLAs contain the redox‐active dbCOT units, we initially investigated their two‐electron reduction using an excess of alkali metals (Li, Na, and K) based on established literature protocols.^[^ ^37^, ^38^ ^]^ However, this approach resulted in the decomposition of the systems in all cases. In contrast, reduction with a stoichiometric amount of potassium graphite (KC_8_) resulted in an immediate quantitative reduction of PLAs **1**–**4** (Figure 1a).

The reduction of the PLAs with fluorine‐containing functions (R = ─CH═CHB(C_6_F_5_)_2_, ─C≡CSb(C_2_F_5_)_2_, C≡CSeCF_3_) did not lead to a conversion in any of the reductions tested, nor was decomposition observed. Even when [2.2.2]cryptand was added prior to KC_8_, no conversion of the fluorinated systems was observed.

The reductions of colourless solutions of **1**–**4** in THF and partially in benzene (**2** and **3**) result in an intense colouration (**1** ^2−^: red, **2** ^2−^: green, **3** ^2−^ and **4** ^2−^: purple; for ultraviolet‐visible (UV–vis) data see Figures S49–S64). The possibility of generating radical anions was also investigated by adding only one equivalent of KC_8_. This led to a 1:1 mixture of neutral and dianionic compounds, which persisted even after heating to 60 °C for several hours.

The reduction process involves the conversion of tub‐shaped non‐aromatic 16 π‐electron systems into planar Hückel‐aromatic 18 π‐electron systems. The ring current effect, resulting from the enlargement of the aromatic system upon reduction, leads to a low‐field shift of the two ^1^H NMR signals associated with the dbCOT unit (see Figure 1b–e). In the systems with vinyl spacers, **1** and **2**, the signals of the vinyl hydrogen atoms are clearly shifted and show an increased shift difference, indicating that the electrons of the vinyl units are involved in the resulting π‐system. Shifts are also observed in the signals corresponding to the heteroatoms, resulting in a broadening of the ^11^B NMR signals of **1A** ^THF^ and the appearance of an additional signal at −13.5 ppm, which disappears after oxidation back to **1** with iodine (see Figures S15 and S19b). A slight shift of the ^29^Si NMR signal upon reduction is observed for the Bis_2_Al‐containing (Bis = ‐CH(SiMe_3_)_2_) compounds **2** and **3** (**2**: −3.6 to −3.4 ppm; **3**: −3.1 to −2.9 ppm, see Figures S29b and S39b). A similar shift from −67.8 to −74.0 ppm is observed for the ^119^Sn resonance of **4/4**K_2_ (SI Figure S48b). All ^13^C resonances for the COT rings exhibit characteristic high‐field shifts (H*C* ^COT^ for PLA: 133–134 ppm, H*C* ^COT^ for PLA^2−^: 100–98 ppm; C*C* ^COT^ for PLA: 138–137 ppm, C*C* ^COT^ for PLA^2−^: 110–108 ppm, see Supporting Information).^[^ ^2^ ^]^

The reduced systems can be converted back to the neutral species by oxidation with iodine. The oxidation of **1** ^2−^ and **4** ^2−^ was also possible using *o*‐chloranil as oxidant. However, when *o*‐chloranil was used in excess, it led to the partial decomposition of the systems (Figure S2). In the case of **2** ^2−^ and **3** ^2−^, due to the higher oxophilicity of the AlBis_2_ than that of the BCy_2_ and SnMe_3_ functions, resulted in an unselective reaction between the oxygen‐containing oxidant *o*‐chloranil and the PLAs **2** and **3**.

In addition to chemical redox‐switchability of the PLA, their electrochemical switchability was tested using cyclic voltammetry under various conditions. However, reversible processes could not be achieved; instead, a shiny metallic coating formed on the electrode surface. We suspect that the applied voltage may cause an over‐reduction of the system, leading to the decomposition of the species, analogous to the above described reactions with an excess of reducing agents. Since the redox potential could not be determined electrochemically, it can only be estimated based on the fact that the compounds can be reduced by lithium, sodium, and potassium, but not by magnesium. This indicates that the redox potential lies between the potentials of magnesium and sodium under the given conditions (benzene or THF as solvents).

### Molecular Structure of the Dianions

The solid‐state structures of the dianions were determined by X‐ray diffraction using single crystals obtained from concentrated solutions at room temperature. In some cases, crystallisation was achieved only after adding a potassium complexing agent, such as Cy_2_‐18‐crown‐6 or [2.2.2]cryptand.^[^ ^47^ ^]^

**Figure 2:**
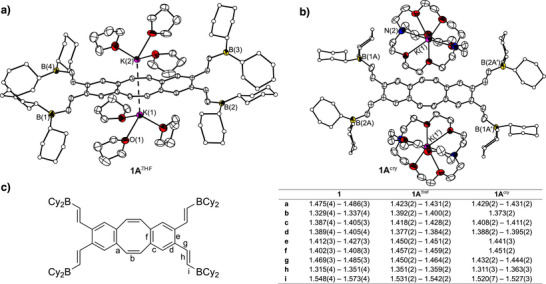
a) and b) show the molecular structure of **1A** ^THF^ and **1A** ^cry^ in the crystalline state. Displacement ellipsoids are drawn with a probability of 40%. For reasons of clarity, minor occupied disordered atoms and H atoms are omitted and the carbon atoms of the cyclohexyl groups are only shown as spheres. c) Selected C─C bond lengths (Å) in **1**, **1A** ^THF^, and **1A** ^cry^ and a labelling scheme. Data of **1** were taken from the literature.^[^ ^2^ ^]^

The compounds [{K^+^(THF)_3_}_2_(**1** ^2−^)] (**1A** ^THF^) and [{K^+^([2.2.2]cryptand)}_2_(**1** ^2−^)] (**1A** ^cry^) (Figure 2a,b) were crystallised from concentrated THF solutions.^[^ ^47^ ^]^ The molecular structure of **1A** ^THF^ shows that the potassium cations are coordinated on both sides of the cyclooctatetraene (COT) system in an $\eta^{8}$‐fashion. In comparison, the K^+^ ions in **1A** ^cry^ are completely enclosed by the ligand, so that the dbCOT does not interact with the cations. This results in the structure of the “naked” dianion **1** ^2−^, allowing a comparison of the structural motif of **1**, **1A** ^THF^ and **1** ^2−^ without the direct influence of the metal bond (Figure 2). The trends in bond length changes (Figure 2c) are consistent with those reported by Petrukhina *et al.* for the reduction of unsubstituted dbCOT,^[^ ^38^ ^]^ i.e., bond **a** is shortened and **b** is elongated, which can be attributed to the aromatisation of the COT core. Thus, in the reduced state, bond **a** gains more double bond character and **b** less. The benzene rings also take on a diene character, which is reflected in the longer bonds **c**, **e**, and **f** and in a shorter bond **d**. However, the changes in bond lengths **a**–**f** determined following the reduction of the unsubstituted dbCOT^[^ ^38^ ^]^ are much more pronounced, than those observed after the reduction of **1**. This is because the CHCHBCy_2_ units in **1** are also part of the π‐system formed upon reduction, leading to shorter bonds **g** and **i**, and a longer bond **h**. So the additional electrons added by the reduction are delocalised via a larger π‐system in **1** ^2−^ compared to the unsubstituted dbCOT^2−^, resulting in less pronounced changes in the bond lengths **a**–**f**. The extension of the π‐system causes the vinyl groups to lie in the same plane as the planarised dbCOT system as does the coordination plane of the boron atoms, so that the vacant p‐orbitals are aligned orthogonally to the dbCOT plane.

**Figure 3:**
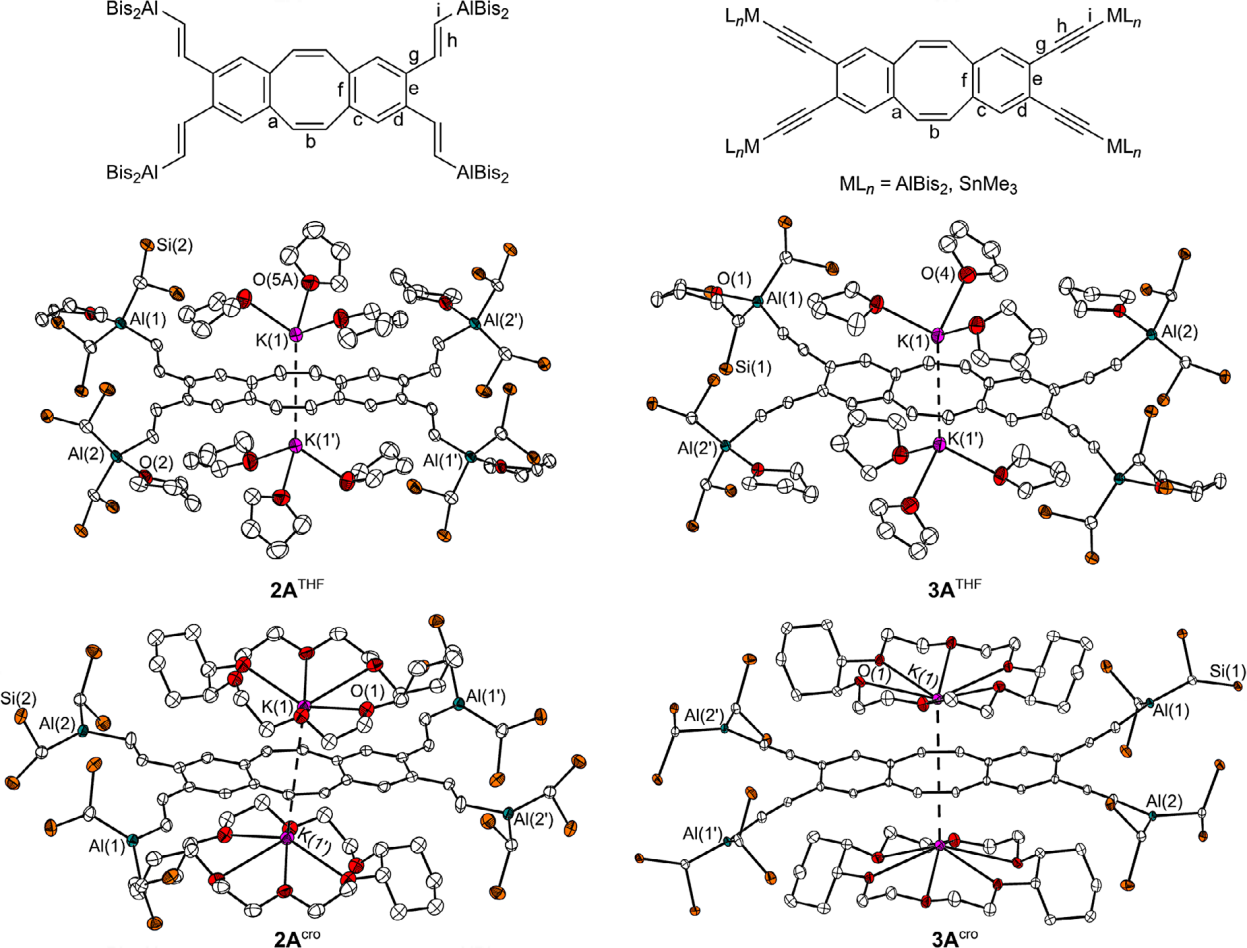
The general Lewis formulae including the bonding nomenclature of the neutral compounds are shown above. Below are the molecular structures of **2A** ^THF^, **3A** ^THF^, **2A** ^cro^, and **3A** ^cro^ in the crystalline state. Displacement ellipsoids are drawn with a probability of 40%. For reasons of clarity, minor occupied disordered atoms, H atoms and the methyl groups are omitted. For more detailed information on the bond lengths, see Tables S1–S3 and Figures S65–S73 for larger images of the molecular structures.^[^ ^47^ ^]^

Crystals suitable for X‐ray diffraction of the reduced aluminium‐containing systems **2** and **3** have been obtained from THF solutions (Figure 3). However, the coordination of THF to the aluminium atoms of these reduced species leads to 1:4 (host:guest) adducts, [{K^+^(THF)_3_}_2_{**2** ^2−^(THF)_4_}] (**2A** ^THF^) and [{K^+^(THF)_3_}_2_ **3** ^2−^(THF)_4_}] (**3A** ^THF^). In order to obtain solid‐state structures of the free PLA^2−^ anions, we conducted the reduction in benzene as a non‐donating solvent, which was not necessary for **1** as this PLA does not form adducts with THF.^[^ ^2^ ^]^ However, the crystallisation of **2** ^2−^ and **3** ^2−^ containing species from benzene was only achieved by adding Cy_2_‐18‐crown‐6 or [2.2.2]cryptand, so that the molecular structures of [{K^+^(Cy_2_‐18‐crown‐6)}_2_(**2** ^2−^)] (**2A** ^cro^), [{K^+^([2.2.2]cryptand)}_2_(**2** ^2−^)] (**2A** ^cry^) and [{K^+^(Cy_2_‐18‐crown‐6)}_2_(**3** ^2−^)] (**3A** ^cro^), could be determined by means of X‐ray diffraction (Figures 3 and S69).^[^ ^47^ ^]^ Despite several attempts, a molecular structure of [{K^+^([2.2.2]cryptand)}_2_(**3** ^2−^)] could not be elucidated due to limited data quality. However, suitable single crystals of **4** ^2−^ were obtained from THF with [2.2.2]cryptand as complexing agent and the structure of [{K^+^([2.2.2]cryptand)}_2_(**4** ^2−^)] (**4A** ^cry^) was determined (see Figure S73).

All solid‐state structures of the reduced systems show significant variations in the lengths of their bonds **a**–**f** in comparison to their neutral counterparts–analogous to the changes previously mentioned for the reduction of **1** (see Tables S1–S3). Changes in the bond lengths **g**, **h**, and **i** of the spacers (ethenyl and ethynyl) were also observed.

In all cases, the bonds **g** and **i** are shorter than in their neutral counterparts or to comparable compounds known from literature which also contain the spacer together with the AlBis_2_ unit.^[^ ^5^, ^26^, ^27^ ^]^ This indicates that the reduction of compounds **1**–**4** leads to the formation of a π‐system, which extends over the dbCOT system and the spacers, and includes the metal substituents. A comparison of the solid‐state structure of the reduced THF adduct **2A** ^THF^ with the neutral adduct **2**·4THF also shows an extension of the Al─O bonds (**2**·4THF: 1.940(2)–1.962(2) Å, **2A** ^THF^: 1.962(2)–1.980(2) Å) after reduction. In the alkyne containing system **3**, the reduction of the THF adduct **3**·4THF shows little or no influence on the Al─O bond (**3**·4THF: 1.928(3)–1.935(3) Å, **3A** ^THF^: 1.935(2)–1.941(2) Å).

This can be related to the higher Lewis acidity of the ─C≡CAlBis_2_ unit compared to the ─CH═CHAlBis_2_ unit.^[^ ^2^ ^]^ The longer Al─O bond of the reduced species demonstrates that the Lewis acid‐base interaction between the PLAs and the base molecules depends on the redox state.

A structural comparison of similar systems with different potassium ligands reveals that the position of the potassium cation significantly affects the bond lengths, whether it is located at the COT units or encapsulated by a cryptand. When the potassium cation is coordinated directly to the COT units, the changes in bond lengths **a** and **b** are more pronounced than when the potassium is sequestered by the cryptand and does not interact with the COT. Conversely, changes in bond lengths **g** and **i** are greater when the potassium cation is complexed by the cryptand.

This observation aligns with the colour change seen upon addition of cryptands, suggesting an increased delocalisation of the π‐system. In systems where potassium binds to the COT, the two additional electrons are more strongly localised on the COT units. In contrast, when potassium is complexed by the cryptand and does not interact with the COT, these electrons tend to be more localised on the Lewis acidic units.

### Quantum Chemical Calculations

In order to gain further insight into the electronic structures of **1**/**1** ^2−^ and **3**/**3** ^2−^, we performed density functional theory (DFT) calculations and natural bond orbital (NBO) analyses. These two systems were chosen to consider both the influence of the different spacers (vinyl versus alkynyl) and the different metals (boron versus aluminium). Furthermore, the switchability of the adducts of **1** and **3** was analysed in more detail in the studies mentioned below. NBO analyses are based on PBE0/def2‐TZVP wave‐functions.

**Figure 4:**
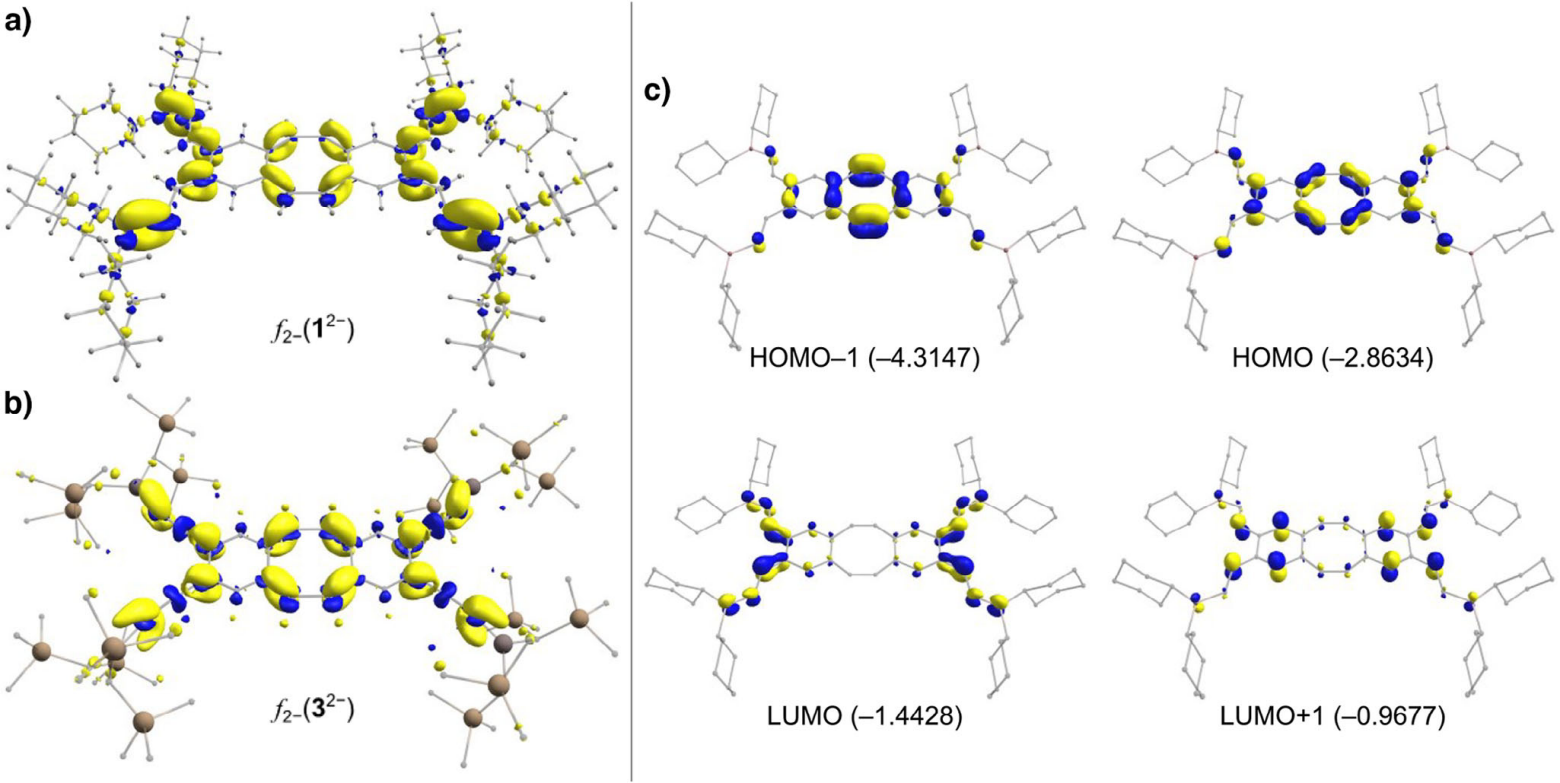
a) shows the electron density difference of *f*_2−_(**1** ^2−^) and b) that of *f*_2−_(**3** ^2−^). The areas shown in yellow are those with a higher electron density and those shown in blue are those with a lower electron density. c) selected molecular orbitals (at PBE0/def2‐TZVP, 0.05 au isosurfaces) of compound **1** ^2−^ with the respective energies (in eV).

Wiberg bond indices (WIBs, Table S9) obtained from this analysis are consistent with the trends determined from the molecular structures, i.e., the bond orders of the C─C multiple bonds (**b**, **h**) decrease, whereas the bond orders of the original C─C single bonds (**a**, **g**) increase in the reduced species **1** ^2−^ and **3** ^2−^ compared to those of the neutral analogues. Furthermore, an increase in the bond orders of the C─M bonds (labelled **i** in Figure 3) of **1** ^2−^ and **3** ^2−^ can be recognised after the reduction. The natural charges from the NBO analyses (Table S9), show a decrease of the positive charge on the metal atoms by 0.19 *e* for the pair **1**/**1** ^2−^ and by 0.1 *e* for the pair **3**/**3** ^2−^ upon reduction. These results support the conclusion drawn from the molecular structures that the two‐electron reduction results in the formation of a π‐system that extends over the spacers to the Lewis acid functions. In order to estimate the localisation of the two extra electrons in **1** ^2−^ and **3** ^2−^, electron density differences (*f*_2−_) were calculated in analogy to Fukui functions.^[^ ^48^ ^]^ *f*_2−_ is defined by *f*_2−_ = *ρ_N_* − *ρ_N_*_−2_, where *ρ_N_* is the electron density of the dianion with a total of *N* electrons and *ρ_N_*_−2_ is the electron density for the same structure with two electrons removed (Figure 4). The resulting plots show the presence of increased electron density after reduction, indicated by yellow colouring, and decreased electron density, indicated by blue colouring. In both cases, reduction leads to a significant increase in electron density of bond **a**, and similarly for bonds **g** and **i** of the spacers. In addition, no substantial change in electron density is observed within the six‐membered rings. The substituents of the metal atoms (Cy and Bis) also show no significant change in electron density, because they are not part of the forming π‐system. This observation indicates also that the electron density associated with the two additional electrons is predominantly localised within bonds **a**, **g**, and **i**.

We also calculated the fractional occupation number weighted density (FOD) for **1**, **1** ^2−^, **3**, and **3** ^2−^. FOD analyses provide information about the localisation of so‐called “hot” (strongly correlated and chemically active) electrons in a molecule (see Figures S80–S83).^[^ ^49^ ^]^ The resulting FOD numbers (*N*_FOD_) for **1**/**1** ^2−^ (1.93 *e*/3.48 *e*) and **3**/**3** ^2−^ (2.33 *e*/3.88 *e*) demonstrate a marked increase of “hot” electrons upon reduction, suggesting a moderate to high level of electron correlation. The FOD plot of the dianion **1** ^2−^ displays the distribution of highly correlated electrons across the dbCOT system and the ─CH═CHB substituents (see Figure S81). Furthermore, **3** ^2−^ also displays a distribution across the dbCOT system (see Figure S83). However, in contrast to **1** ^2−^, in which the boron atoms clearly contribute to FOD, the aluminium atoms in **3** ^2−^ are significantly less involved.

As a representative of the other compounds, the orbitals involved in the electron transition of **1** ^2−^ are responsible for the extreme colouring of the reduced species and were examined more closely using TD‐DFT calculations to simulate UV–vis spectra (see the Supporting Information for details). Experimentally, the UV–vis spectrum of **1A** ^THF^ shows two absorption bands of almost equal intensity ($\lambda_{\max}$ = 515, 885 nm). The TD‐DFT calculations attribute these absorption bands to the transitions HOMO−1 → LUMO+1 (λ = 439 nm) and HOMO → LUMO (λ = 844 nm). Figure 4c shows the corresponding orbitals. The calculations indicate the presence of a small HOMO─‐LUMO gap of 1.4 eV, with the highest occupied molecular orbital (HOMO) and HOMO−1 of **1** ^2−^ primarily located on the dbCOT unit, and the lowest unoccupied molecular orbital (LUMO) predominantly found on the ─CH═CHBCy_2_ units.

### Redox Switchability of Lewis Acid‐Base Interactions

**Figure 5:**
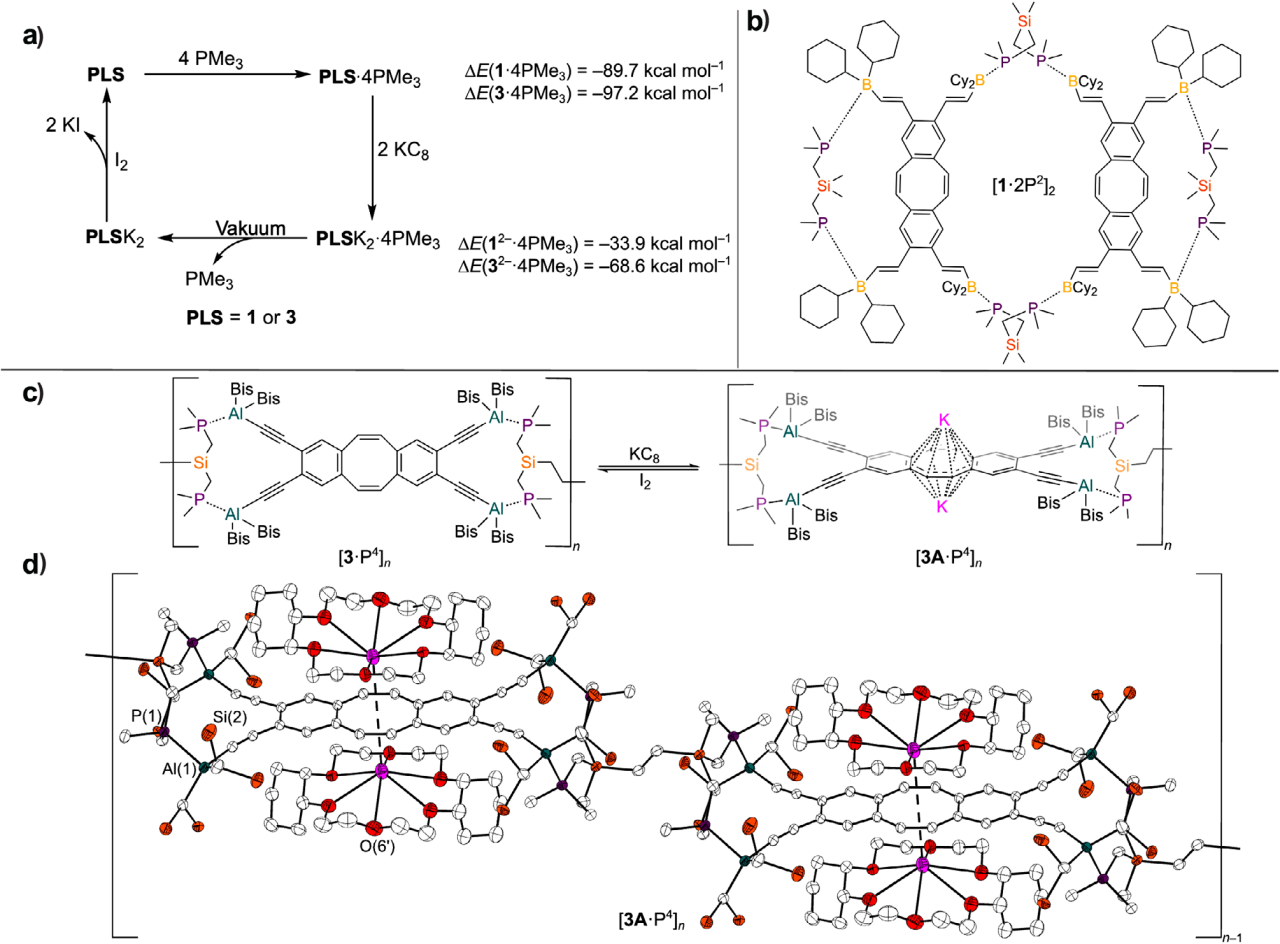
Investigations on the redox switchability of the adducts. a) shows the reduction‐induced release of PMe_3_ from the corresponding 1:4 adducts due to the weakening of the interaction, as well as the calculated energies (*∆E*). b) shows the Lewis structural formula of [**1**·2P^2^]_2_. c) schematic of the switchability of the Al─P coordination polymer [**3**·P^4^]*_n_* to [**3**K_2_·P^4^]*_n_* and d) shows the molecular structure in the solid‐state of [**3**K_2_·P^4^]*_n_* with Cy_2_‐18‐crown‐6 as potassium complex former. Displacement ellipsoids, drawn with a probability of 40%. For reasons of clarity, slightly occupied disordered atoms, H atoms and the methyl groups are omitted. For more detailed information on the bond lengths, see Table S3 and Figure S74 for more images of the molecular structure.

**Figure 6:**
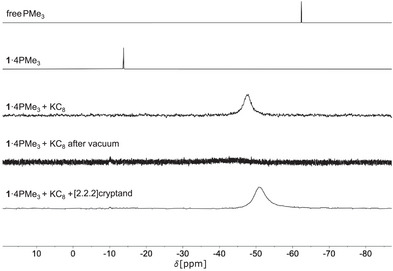
^31^P NMR spectra of the redox switching experiments with **1**·4PMe_3_, after addition of KC_8_, after addition of KC_8_ and drying in vacuum and after reduction with KC_8_ and addition of [2.2.2]cryptand in THF‐*d*_8_ at 298 K, 202 MHz.

The influence of reduction on the interaction between the PLAs and the Lewis bases was studied by adduct formation of PLAs **1** and **3** with different phosphanes, including PMe_3_, bis[(dimethylphosphanyl)methyl]dimethylsilane (P^2^) and 4,7‐bis[(dimethylphosphanyl)methyl]‐2,4,7,9‐tetramethyl‐2,9‐diphospha‐4,7‐disiladecane (P^4^), as Lewis bases. These phosphanes represent suitable probes for the investigation of Lewis acid‐base interactions, as the ^31^P{^1^H} NMR signal exhibits characteristic low‐field shifts upon adduct formation. Since **2** showed only a weak (PMe_3_ is removable from the adduct by applying vacuum) and **4** no interaction with the tested phosphanes (NMR spectroscopy), we did not investigate them in more detail. The reaction of **1** and **3** with PMe_3_ led to the formation of stable 1:4 adducts (**PLA**·4PMe_3_) immediately after addition, which do not release the bound phosphanes, even under harsh conditions of reduced pressure and elevated temperature (0.003 mbar, 80 °C, >1 h). However, when the adducts **1**·4PMe_3_ or **3**·4PMe_3_ are reduced by KC_8_, the interactions between the Lewis acid functions and the base PMe_3_ are weakened. This is evident from the high‐field shift of the ^31^P{^1^H} signals after reduction. The signals thus approach the chemical shift of free PMe_3_ at −62.4 ppm. After reduction, the phosphane can be removed at room temperature under reduced pressure and is then no longer detectable by ^31^P{^1^H} NMR in the redissolved residue. Instead, the material removed under reduced pressure contain only the respective solvent and free PMe_3_. Subsequent oxidation of the reduced **PLA**K_2_ systems using iodine results in the recovery of the free PLAs **1** or **3** and completes the reaction cycle (see Figure 5a). The reduced systems **1A** ^THF^ and **3A** contain two potassium cations per PLA unit. Because the potassium cations are Lewis acids, they can interact directly with PMe_3_. They also affect the distribution of electrons in the π‐system through their interaction with the COT unit and thus indirectly influence the interaction of the Lewis acidic unit and PMe_3_. To investigate the influence of potassium, [2.2.2]cryptand was added in a separate experiment after the reduction of the PMe_3_ adducts. This removes the potassium cations from the COT moieties and inhibits their reactivity. The ^31^P{^1^H} NMR spectra (see Figures 6 and S8) show that this addition further weakens the interaction between the Lewis acid and PMe_3_, which is reflected in an approximation of the phosphorus signals to −62.4 ppm. This allows the strength of the Lewis acidity of the system to be further decreased and thus to be controlled by the reagents complexing the potassium cations. This is also consistent with the previously discussed changes in bond lengths observed in the different reduced systems–namely, when potassium is coordinated to the COT units versus when it is complexed by the cryptand.

In order to take a closer look on the weakening of the interaction between PMe_3_ and the Lewis acid units upon reduction, we calculated the energies (*∆E*) for the reaction of PMe_3_ with the neutral compounds **1** and **3** and with the dianionic compounds **1** ^2−^ and **3** ^2−^. The calculations were performed on geometrically optimised structures at the PBEh‐3c level of theory. The binding energies of the neutral adducts are −89.7 for **1**·4PMe_3_ and −97.2 kcal mol^−1^ for **3**·4PMe_3_. The reduction process leads to the formation of energetically less stable species, with binding energies *∆E* of −33.9 for **1** ^2−^·4PMe_3_ and −68.6 kcal mol^−1^ for **3** ^2−^·4PMe_3_. This is consistent with and quantifies the experimental findings of a weakening of the interaction between the PLA and PMe_3_ upon reduction.

In addition to the PMe_3_ adducts, the effect of reduction on the more complex adducts [**1**·2P^2^]_2_ and [**3**·P^4^]*_n_* was also investigated (see Figure 5b,c). The adduct [**1**·2P^2^]_2_, a 2:4 adduct in both the solid and liquid phases,^[^ ^2^ ^]^ was reduced using KC_8_ and subsequently oxidised with *o*‐chloranil. The ^31^P{^1^H} NMR signal shifts from −7.1 ppm for [**1**·2P^2^]_2_ upon reduction to −47.8 ppm and back to −7.1 ppm after oxidation (see Figure S10). The ^1^H NMR spectrum displays markedly sharper signals after the reduction in comparison to those observed for [**1**·2P^2^]_2_, thereby supporting the hypothesis that the 2:4 adduct [**1**·2P^2^]_2_ undergoes dissociation. This is consistent with the hypothesis that the complexation of P^2^ in the intramolecular binding pocket is no longer feasible due to the planarization of the dbCOT system. However, after oxidation, strongly broadened signals arise which deviate slightly from those of the previously used [**1**·2P^2^]_2_. The connectivity of the adduct formed after oxidation could not be clarified in more detail.

In comparison with [**1**·2P^2^]_2_, the coordination polymer [**3**·P^4^]*_n_* does not complex the base across the cavity,^[^ ^2^ ^]^ resulting in the Al─P bond remaining intact during reduction of the adduct [**3**·P^4^]*_n_*. Following the oxidation of the reduced coordination polymer [**3A**·P^4^]*_n_* with iodine, the resulting NMR spectra exhibited the same signals as prior to reduction, thereby indicating that this coordination polymer can be reversibly switched between these two states. It should be noted that [**3**·P^4^]*_n_* crystallises quickly from benzene upon the addition of **3** and P^4^, and the resulting polymer is poorly soluble. Consequently, the benzene suspension containing crystalline [**3**·P^4^]*_n_* and KC_8_ must be heated to 80 °C to enable the reduction of [**3**·P^4^]*_n_*. The reduced species is more soluble and does not crystallise immediately from a dilute solution; crystallization only occurs after the addition of Cy_2_‐18‐crown‐6 and concentration of the solution. It therefore remains unclear whether the coordination polymer is reduced or whether heating causes the polymer to dissociate into smaller oligomers or monomers, which are then reduced in solution. These reduced species could subsequently reassemble into a coordination polymer during crystallization. The single crystals obtained were suitable for structure determination by X‐ray diffraction (Figure 5d).^[^ ^47^ ^]^ The molecular structure shows the complete 2*n*‐fold reduction of [**3**·P^4^]*_n_* to [**3A**·P^4^]*_n_*, with two potassium cations complexed on both sides of the COT unit of the PLA units of the coordination polymer. The potassium ions are further coordinated by the Cy_2_‐18‐crown‐6 bases. As described above for all reduced systems, planarisation of the COT units contribute to the formation of a planar π‐system across the dbCOT and the spacers. Accordingly, there are changes in the lengths of the bonds **a**–**i** (Table S3) in the same way as described for **3A** ^THF^ and **3A** ^cro^. A change in the Al─P bond lengths, which covers a size range of 2.481(1)–2.524(1) Å was not observed. Thus, even in the reduced state, the preservation of the Al─P bond appears to be energetically favourable and the reduction does not destabilise the coordination polymer. Consequently, [**3**·P^4^]*_n_* is a stable polymer that can undergo reversible switching via redox reactions, resulting in structural and electronic changes to the polymer. This renders the compound intriguing for the development of functional and switchable materials for electrical and optical applications. The dbCOT units make this system a transition‐metal free redox‐active macromolecule. Compared to the non‐reduced coordination polymer [**3**·P^4^]*_n_* the reduction leads to a elongation of the polymer repetition unit [**3**·P^4^] by 3.651 Å (measured between two aluminium atoms). This resembles the electro‐mechanically switchable hydrocarbons of Strand *et al.*, mentioned in the introduction.^[^ ^39^ ^]^

During these and the previous investigations, it was observed that the molecular structures of the neutral systems often show solvent molecules (such as benzene as well as *n*‐hexane) or even other base molecules, such as bisimidazolylmethane, trapped in the cavity of the PLAs by weak interactions.^[^ ^2^ ^]^ Upon reduction and the resulting structural change of the PLAs, this cavity between the side wings disappears and the molecules trapped in the cavity are released. Consequently, these redox‐switchable PLAs can be also regarded as a form of switchable molecular tweezers.

## Conclusion

In this study, we have demonstrated the redox‐switchability of PLAs based on the *sym*‐dibenzo[*a*,*e*]cyclooctatetraene (dbCOT) framework. The reduction of these systems with potassium graphite results in significant structural and electronic changes, including the planarisation of the dbCOT unit and the formation of an extended π‐system. This delocalisation was observed in crystallographic and detailed in computational studies. The two additional electrons are delocalised over an extended π‐system spanning the dbCOT, the alkene/alkyne spacers and the Lewis acid sites, which reduces their acidity. The reduced PLAs could also be oxidised back to the neutral compounds so that it is possible to switch back and forth between these two redox states. The systems are therefore redox‐switchable PLAs. The reduction significantly weakens the acidities of the PLAs and consequently their interaction strengths towards Lewis bases such as PMe_3_, leading to a switchable removability of the phosphane ligands from their complexes, veryfied in a series of experiments. The computed electronic energies of reduced and oxidised forms of the PLA confirmed this observation, demonstrating a substantial energetic destabilisation of the Lewis acid‐base interactions upon reduction. Furthermore, the redoxswitching of the aluminium‐phosphane coordination polymer leads to a pronounced variation in length of the polymer repetition unit, which highlights the potential of these system as functional materials for electronic and optical applications.

Overall, our results establish dbCOT‐based PLAs as a class of redox‐switchable Lewis acids with tuneable electronic properties. These findings offer the potential for the development of responsive molecular materials that could be used in catalysis, molecular switching, and electronic devices.

## Supporting Information

The data supporting the findings of this study are available within the article and Supplementary Information.^[^ ^2^, ^5^, ^26^, ^27^, ^48^, ^50^, ^51^, ^52^, ^53^, ^54^, ^55^, ^56^, ^57^, ^58^, ^59^, ^60^ ^]^ Source data are provided with this paper. CCDC 2441691–2441700 contain the supplementary crystallographic data for this paper.

## Conflict of Interests

The authors declare no conflict of interest.

## Supporting information



Supporting Information

Supporting Information

## Data Availability

The data that support the findings of this study are available in the Supporting Information of this article.
