# Supplementary material for: Redox‐Switchable Poly‐Lewis Acids Allow the Controlled Release of Guests
Source: Angew Chem Int Ed Engl. 2025 Aug 12;64(38):e202509191. doi: 10.1002/anie.202509191 (PMC12435436; doi:10.1002/anie.202509191)
Supplement: Supplementary file 1 — Supporting Information [file ANIE-64-e202509191-s001.pdf]

Supplementary information

Redox-Switchable Poly-Lewis Acids Allow the Controlled Release of Guests

Maximilian J. Klingsiek,[a] Yury V. Vishnevskiy,[a] Julian Buth,[a] Jan-Hendrik Lamm,[a] Beate Neumann,[a] Hans-Georg Stammler,[a] and Norbert W. Mitzel[a]*

[a] Universität Bielefeld, Fakultät für Chemie, Lehrstuhl für Anorganische Chemie und Strukturchemie, Center for Molecular Materials CM_2, Universitätsstraße 25, D-33615 Bielefeld,
E-Mail: mitzel@uni-bielefeld.de; FAX: +49 521 106 6020; Tel; +495211066128

Table of contents

General Information 2

Synthesis and Characterization 3

Preparation of [{K^+(THF)$_3$}$_2$(**1**$^{2-}$)] (**1A**^{THF}) 3

Preparation of [{K^+(Cy_2-18-crown-6)(THF)$_2$}$_2$(**1**$^{2-}$)] (**1A**^{cro}) and [{K^+([2.2.2]cryptand)}$_2$(**1**$^{2-}$)] (**1A**^{cry}) 3

Preparation of [{K^+(THF)$_3$}$_2${**2**$^{2-}$ (THF)$_4$}] (**2A**^{THF}) 4

Preparation of [{K^+}$_2${**2**$^{2-}$}] (**2A**) 4

Preparation of [{K^+(THF)$_3$}$_2${**3**$^{2-}$(THF)$_4$}] (**3A**^{THF}) 4

Preparation of [{K^+}$_2${3^{2-}}] (**3A**) 4

Preparation of [{K^+(Cy_2-18-crown-6)}$_2$(**2**$^{2-}$)] (**2A**^{cro}), [{K^+([2.2.2]cryptand)}$_2$(**2**$^{2-}$)] (**2A**^{cry}), [{K^+(Cy_2-18-crown-6)}$_2$(**3**$^{2-}$)] (**3A**^{cro}) and [{K^+([2.2.2]cryptand)}$_2$(**3**$^{2-}$)] (**3A**^{cry}) 5

Preparation of [{K^+([2.2.2]cryptand)}$_2$(**4**$^{2-}$)] (**4A**^{cry}) 5

Investigations into the reduction of fluorinated PLAs [$(C_6F_5)_2$BCHCH]$_4$–, [$(C_2F_5)_2$SbCC]$_4$–, [F_3CSeCC]$_4$–dbCOT5

NMR-Investigations 6

NMR-investigation of redox switchability with *o*-chloranil 6

NMR-investigation of redox switchability with iodine 6

NMR-investigations into the switchability of PMe_3 adducts 7

NMR investigations on the switchability of [**1**·2P^2]$_2$ 10

NMR investigations on the reduction of [**3**·P^4]$_n$ 10

NMR Spectra 13

Compound **1A**^{THF} 13

Compound **1A**^{cro} 14

Compound **1A**^{cry} 17

Compound **2A**^{THF} 18

Compound **2A** 20

Compound **2A**^{cro} 22

Compound **2A**^{cry} 22

Compound **3A**^{THF} 24

Compound **3A** 25

Compound **3A**^{cro} 27

Compound **3A**^{cry} 28

Compound **4A**^{THF} 30

UV-Vis Spectra 33

X-ray Crystallography 41

Quantum Chemical Calculations 50

References 61

General Information

Compounds **1**–**4** have been synthesized according to the synthetic route we have already reported.[1] All operations with air and moisture sensitive compounds were performed under conventional Schlenk technique or in gloveboxes under inert nitrogen or argon atmosphere. The solvents THF (potassium) and benzene (NaK) were dried by common methods and freshly distilled before use. THF-d_8 and C_6D_6 was dried over NaK and stored over molecular sieve (4 Å) after vacuum-transferred.

NMR spectra were recorded on a Bruker Avance III 500 and Bruker Avance III 300. The chemical shifts (δ) were measured in ppm (parts per million) and the spectra were referenced to the residual signal of proton-containing

solvents ($CDCl_3$: ^{1}H NMR, δ = 7.26 ppm, ^{13}C NMR, δ = 77.16 ppm; C_6D_6: ^{1}H NMR, δ = 7.16 ppm, ^{13}C NMR, δ = 128.06 ppm) or external standards (^{11}B: $BF_3{\cdot}Et_2O$, ^{29}Si: $SiMe_4$, ^{31}P: 85% H_3PO_4, ^{119}Sn: $SnMe_4$).
Elemental analyses were performed with *HEKAtech EURO EA* instrument (too low carbon values due to the formation of silicon, boron or aluminium carbide). The extreme air- and moisture sensitivity as well as the formation of boron, silicon and aluminium carbide of crystals **1A**^{cry}, **2A**^{cry} and **3A**^{cro} prevented us from obtaining suitable and reproducible elemental analysis data.
The UV−vis spectra were recorded on a Thermo Scientific Genesys 50 UV−visible spectrophotometer.
The numbering schema for NMR spectroscopic assignments is shown in Scheme S1.

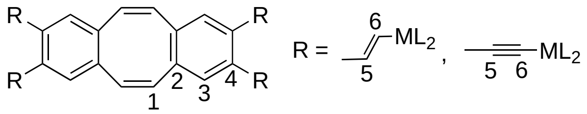

Scheme S1. Numbering scheme for NMR spectroscopic assignments

Synthesis and Characterization

Preparation of [{K^{+}(THF)$_3$}$_2$(**1**$^{2-}$)] (**1A**^{THF})

KC_8 (10 mg, 74 µmol, 2.2 eq.) was added to a solution of compound **1** (33 mg, 33 µmol, 1.0 eq.) in THF (1 mL). The solution immediately turned to a deep dark red colour. After 2 min the suspension was filtered to remove any graphite and the residue was washed with THF (0.3 mL). The resulting dark red solution was gradually concentrated under reduced pressure to give **1A**^{THF} as a dark red crystalline solid. The resulting single crystals were suitable for determining the solid-state structure using X-ray diffraction experiments. As can be seen from the elemental analysis, the THF free species [{K^{+}}$_2$(**1**$^{2-}$}] (35 mg) is obtained as a dark red solid after extensive drying in a vacuum.
^{1}H NMR (500 MHz, THF-d_8) δ = 8.35 (s, 4H, **H**^{3}), 8.31 (d, $^{3}J_{H,H}$ = 17.2 Hz, 4H, **H**^{5}), 7.18 (s, 4H, **H**^{1}), 6.73 (d, $^{3}J_{H,H}$ = 17.0 Hz, 4H, **H**^{6}), 1.85–1.77 (m, 6H, **H**^{Cy}), 1.67–1.60 (m, 5H, **H**^{Cy}), 1.48–1.36 (m, 11H, **H**^{Cy}) ppm. – ^{11}B NMR (160 MHz, THF-d_8) δ = −13.8 ppm. – $^{13}C\{^{1}H\}$ NMR (126 MHz, THF-d_8) δ = 150.3 (**C**^{5}), 139.1 (**C**^{4}), 120.2 (**C**^{2}), 119.8 (**C**^{6}), 113.6 (**C**^{3}), 107.9 (**C**^{1}), 33.6 (**C**^{Cy}), 29.1 (**C**^{Cy}), 27.8 (**C**^{Cy}), 27.3 (**C**^{Cy}). – UV-vis (THF, λ_{max}, nm): 885, 515. – Elemental analysis calcd (%) for $C_{72}H_{104}B_4K_2$ (M_r = 1091.06): C 79.26, H 9.61; found C 78.81, H 9.45.

Preparation of [{K^{+}(Cy$_2$-18-crown-6)(THF)$_2$}$_2$(**1**$^{2-}$)] (**1A**^{cro}) and [{K^{+}([2.2.2]cryptand)}$_2$(**1**$^{2-}$)] (**1A**^{cry})

Compound **1A**^{cro} and **1A**^{cry} were prepared as described for **1A**^{THF} except that 18-crown-6 or [2.2.2]cryptand was added after filtration.

1A^{cro} (21 mg, 11 µmol, 85%) from **1** (13 mg, 13 µmol, 1.0 eq.), KC_8 (4.0 mg, 30 µmol, 2.3 eq.), THF (0.5 mL), Cy$_2$-18-crown-6 (11 mg, 30 µmol, 2.3 eq.).
^{1}H NMR (500 MHz, THF-d_8) δ = 8.18 (d, $^{3}J_{H,H}$ = 16.7 Hz, 4H, **H**^{5}), 8.07 (s, 4H, **H**^{3}), 6.82 (s, 4H, **H**^{1}), 6.50 (d, $^{3}J_{H,H}$ = 16.7 Hz, 4H, **H**^{6}), 3.36–3.15 (m, 88H, **H**^{cro}/ex. Cy$_2$-18-crown-6), 1.67 (s, 28H, **H**^{cro}/**H**^{Cy}/ex Cy$_2$-18-crown-6), 1.56–0.96 (m, 168H, **H**^{cro}/**H**^{Cy}/ex Cy$_2$-18-crown-6) ppm. – ^{1}H NMR (500 MHz, C_6D_6) δ = 8.92 (d, $^{3}J_{H,H}$ = 16.9 Hz, 4H, **H**^{5}), 8.77 (s, 4H, **H**^{3}), 7.44 (s, 4H, **H**^{1}), 7.23 (d, $^{3}J_{H,H}$ = 16.9 Hz, 4H, **H**^{6}), 3.31–2.70 (m, 48H, **H**^{cro}), 2.09–1.92 (m, 44H, **H**^{cro}/**H**^{Cy}), 1.63–1.46 (m, 44H, **H**^{cro}/**H**^{Cy}), 1.46–0.98 (m, 24H, **H**^{Cy}) ppm. – ^{11}B NMR (160 MHz, THF-d_8) δ = −13.8 ppm. – ^{11}B NMR (160 MHz, C_6D_6) δ = −13.6 ppm. – $^{13}C\{^{1}H\}$ NMR (126 MHz, C_6D_6) δ = 150.2 (**C**^{5}), 139.5 (**C**^{4}), 120.0 (**C**^{2}), 115.9 (**C**^{3}), 110.9 (**C**^{1}), 77.7 (**C**^{cro}), 70.2 (**C**^{cro}), 69.9 (**C**^{cro}), 66.0 (**C**^{cro}), 34.0 (**C**^{Cy}), 29.7 (**C**^{Cy}), 28.3 (**C**^{cro}), 27.7 (**C**^{Cy}), 25.5 (**C**^{cro}), 21.5 (**C**^{Cy}) ppm. – UV-vis (THF, λ_{max}, nm): 1030, 610, 570, 530, 485. – Elemental analysis calcd (%) for $C_{112}H_{176}B_4K_2O_{12}$ (M_r = 1836.06): C 73.27, H 9.66; found C 72.91, H 9.42.

1A^{cry} (27 mg, 15 µmol, 91%) from **1** (16 mg, 16 µmol, 1.0 eq.), KC_8 (5.0 mg, 37 µmol, 2.2 eq.), THF (0.5 mL), [2.2.2]cryptand (14 mg, 37 µmol, 2.2 eq.).
^{1}H NMR (500 MHz, THF-d_8) δ = 8.43–8.03 (m, 4H, **H**^{5}/**H**^{3}), 6.75–6.20 (m, 4H, **H**^{6}/**H**^{1}), 3.62 (s, 24H, **H**^{cry}), 3.56 (t, $^{3}J_{H,H}$ = 5.4 Hz, 24H, **H**^{cry}), 2.60 (t, $^{3}J_{H,H}$ = 5.4 Hz, 24H, **H**^{cry}), 1.86–1.79 (m, 20H, **H**^{Cy}), 1.66 (m, 24H, **H**^{Cy}), 1.45 (m, 44H, **H**^{Cy}) ppm.– No interpretable ^{11}B and $^{13}C\{^{1}H\}$ NMR spectra could be obtained. – UV-vis (THF, λ_{max}, nm): 1040, 620, 580, 537, 485.

Preparation of [{K^+(THF)$_3$}$_2${**2**$^{2-}$ (THF)$_4$}] (**2A**^{THF})

KC_8 (5.4 mg, 40 µmol, 2.2 eq.) was added to a solution of compound **2** (31 mg, 18 µmol, 1.0 eq.) in THF (0.5 mL). The solution immediately turned to a deep dark green colour. After 5 min the suspension was filtered to remove any graphite and the residue was washed with THF (0.3 mL). The resulting dark green solution was gradually concentrated under reduced pressure to give **2A**^{THF} as a dark green needle-shaped crystalline solid. The resulting single crystals were suitable for determining the solid-state structure using X-ray diffraction experiments. As can be seen from the elemental analysis, the THF free species [{K^+}$_2${**2**$^{2-}$}] (25 mg, 14 µmol, 70%) is obtained as a dark green solid after extensive drying in a vacuum.
^{1}H NMR (500 MHz, THF-d_8) δ = 8.21 (s, 4H, **H**^{3}), 7.51 (d, $^3J_{H,H}$ = 19.7 Hz, 4H, **H**^{5}), 7.19 (s, 4H, **H**^{1}), 6.44 (d, $^3J_{H,H}$ = 19.8 Hz, 4H, **H**^{6}), 0.23 (s, 144H, Si(C**H**$_3$)$_3$), −0.98 (s, 8H, AlC**H**) ppm. ^{13}C{^{1}H} NMR (126 MHz, THF-d_8) δ = 145.0 (**C**^{5}), 132.3 (**C**^{3}), 130.8 (**C**^{6}), 122.5 (**C**^{4}), 109.5 (**C**^{2}), 98.4 (**C**^{1}), 4.6 (Si**C**H$_3$), 1.5 (Al**C**H) ppm. – ^{29}Si{^{1}H} NMR (99 MHz, THF-d_8) δ = −2.3 ppm. – UV-vis (THF, λ_{max}, nm): 590, 440. – Elemental analysis calcd (%) for $C_{120}H_{248}Al_4K_2O_{10}Si_{16}$ (with 10 THF) (M_r = 2486.77): C 58.15, H9.76; calcd (%) for $C_{80}H_{168}Al_4K_2Si_{16}$ (without THF) (M_r = 1765.71): C 54.42, H 9.59; found C 54.48 H 9.63.

Preparation of [{K^+}$_2${**2**$^{2-}$}] (**2A**)

KC_8 (5.4 mg, 40 µmol, 2.2 eq.) was added to a solution of compound **2** (31 mg, 18 µmol, 1.0 eq.) in benzene (0.5 mL). The solution immediately turned slightly green. After 1 h the suspension was filtered to remove any graphite and the residue was washed with benzene (0.5 mL). The resulting dark green solution was gradually concentrated under reduced pressure to give **2A** (29 mg, 16 µmol, 90%) as a dark green solid.
^{1}H NMR (500 MHz, C_6D_6) δ = 8.68 (s, 4H, **H**^{3}), 8.34 (d, $^3J_{H,H}$ = 19.8 Hz, 4H, **H**^{5}), 7.26 (s, 4H, **H**^{1}), 7.11 (d, $^3J_{H,H}$ = 19.8 Hz, 4H, **H**^{6}), 0.45 (s, 144H, Si(C**H**$_3$)$_3$), −0.07 (s, 8H, AlC**H**) ppm. – ^{13}C{^{1}H} NMR (126 MHz, C_6D_6) δ = 151.2 (**C**^{5}), 135.1 (**C**^{3}), 129.7 (**C**^{6}), 122.9 (**C**^{4}), 109.1 (**C**^{2}), 99.3 (**C**^{1}), 10.0 (Al**C**H), 4.3 (Si**C**H$_3$) ppm. – ^{29}Si{^{1}H} NMR (99 MHz, C_6D_6) δ = −3.42 ppm. – UV-vis (C_6H_6, λ_{max}, nm): 668, 463. – Elemental analysis calcd (%) for $C_{80}H_{168}Al_4K_2Si_{16}$ (M_r = 1765.71): C 54.42, H 9.59; found C 54.61 H 9.39. The elemental analysis induces that all THF molecules can be remove in a vacuum.

Preparation of [{K^+(THF)$_3$}$_2${**3**$^{2-}$(THF)$_4$}] (**3A**^{THF})

KC_8 (4.5 mg, 33 µmol, 2.2 eq.) was added to a solution of compound **3** (25 mg, 15 µmol, 1.0 eq.) in THF (0.5 mL). The solution immediately turned to a deep dark purple. After 5 min the suspension was filtered to remove any graphite and the residue was washed with THF (0.3 mL). The resulting dark purple solution was gradually concentrated under reduced pressure to give [{K^+(THF)$_3$}$_2${**3**$^{2-}$(THF)$_4$}] as a dark purple needle-shaped crystalline solid. The resulting single crystals were suitable for determining the solid-state structure using X-ray diffraction experiments. As can be seen from the elemental analysis, the species [{K^+}$_2${**3**$^{2-}$(THF)$_4$}] (29 mg, 14 µmol, 93%) is obtained as a dark blue-purple solid after extensive drying in a vacuum.
^{1}H NMR (500 MHz, THF-d_8) δ = 8.17 (s, 4H, **H**^{3}), 7.05 (s, 4H, **H**^{1}), 0.23 (s, 144H, Si(C**H**$_3$)$_3$), −1.12 (s, 8H, AlC**H**) ppm. – ^{13}C{^{1}H} NMR (126 MHz, THF-d_8) δ = 143.2 (**C**^{3}), 115.3 (**C**^{5}), 108.8 (**C**^{2}), 104.3 (**C**^{4}), 97.7 (**C**^{1}), 4.4 (Si**C**H$_3$), 1.3 (Al**C**H) ppm, no signal for C**C**Al (**C**^{6}) was observed. – ^{29}Si{^{1}H} NMR (99 MHz, THF-d_8) δ = −1.91 ppm. – UV-vis (THF, λ_{max}, nm): 534, 500, 409, 382. – Elemental analysis calcd (%) for $C_{120}H_{240}Al_4K_2O_{10}Si_{16}$ (with 10 THF) (M_r = 2478.71): C 58.15, H 9.76; calcd (%) for $C_{96}H_{192}Al_4K_2Si_{16}$ (with 4 THF) (M_r = 2046.07): C 56.35, H 9.46; calcd (%) for $C_{80}H_{160}Al_4K_2Si_{16}$ (without THF) (M_r = 1757.64): C 54.67, H 9.18; found C 55.00, H 9.44. Elemental analysis suggests removal of the THF molecules complexed to potassium under reduced pressure, whereas those complexed by the CCAlBis$_2$ unit remain bound; this is consistent with previous studies.[1]

Preparation of [{K^+}$_2${**3**$^{2-}$}] (**3A**)

KC_8 (4.5 mg, 33 µmol, 2.2 eq.) was added to a solution of compound **3** (25 mg, 15 µmol, 1.0 eq.) in benzene (0.5 mL). The solution immediately turned slightly green to purple. After 1 h the suspension was filtered to remove any graphite and the residue was washed with benzene (0.5 mL). The resulting dark purple solution was gradually concentrated under reduced pressure to give **3A** (22 mg, 13 µmol, 83%) as a dark green solid.
^{1}H NMR (500 MHz, C_6D_6) δ = 8.71 (s, 4H, **H**^{3}), 7.26 (s, 4H, **H**^{1}), 0.62 (s, 144H, Si(C**H**$_3$)$_3$), −0.12 (s, 8H, AlC**H**) ppm. – ^{13}C{^{1}H} NMR (126 MHz, C_6D_6) δ = 143.5 (**C**^{3}), 117.1 (**C**^{5}), 108.1 (**C**^{2}), 104.9 (**C**^{4}), 102.1 (**C**^{6}), 97.9 (**C**^{1}), 10.8 (Al**C**H), 4.2 (Si**C**H$_3$) ppm. – ^{29}Si{^{1}H} NMR (99 MHz, C_6D_6) δ = −2.9 ppm. – UV-vis (C_6H_6, λ_{max}, nm): 555, 416. – Elemental analysis calcd (%) for $C_{80}H_{160}Al_4K_2Si_{16}$ (M_r = 1757.64): C 54.67, H 9.18; found C 54.68, H 9.10.

Preparation of $[\{K^+(Cy_2\text{-}18\text{-crown-}6)\}_2(\mathbf{2}^{2-})]$ (**2A**^{cro}), $[\{K^+([2.2.2]cryptand)\}_2(\mathbf{2}^{2-})]$ (**2A**^{cry}), $[\{K^+(Cy_2\text{-}18\text{-crown-}6)\}_2(\mathbf{3}^{2-})]$ (**3A**^{cro}) and $[\{K^+([2.2.2]cryptand)\}_2(\mathbf{3}^{2-})]$ (**3A**^{cry})

Compounds **2A**^{cro} and **2A**^{cry} were prepared as described for **2A** and **3A** except that Cy_2-18-crown-6 or [2.2.2]cryptand was added after filtration. Single crystals of all four compounds suitable for X-ray diffraction experiments were obtained from concentrated benzene solutions at room temperature.

2A^{cro} (14 mg, 5.5 µmol, 87%) from **2** (11 mg, 6.3 µmol, 1.0 eq.), KC_8 (2.0 mg, 15 µmol, 2.4 eq.), benzene (0.5 mL), Cy_2-18-crown-6 (5.6 mg, 15 µmol, 2.4 eq.).
^{1}H NMR (500 MHz, C_6D_6) δ = 8.61 (s, 4H, **H**^{3}), 8.17 (d, $^3J_{H,H}$ = 19.4 Hz, 4H, **H**^{5}), 7.52 (s, 4H, **H**^{1}), 6.79 (d, $^3J_{H,H}$ = 19.5 Hz, 4H, **H**^{6}), 3.79–2.74 (m, 72H, **H**^{cro} + ex. Cy_2-18-crown-6), 2.00–1.12 (m, 72H, **H**^{cro} + ex. Cy_2-18-crown-6), 0.50 (s, 144H, $Si(C\mathbf{H}_3)_3$), −0.05 (s, 8H, AlC**H**) ppm – Due to the low solubility of **2A**^{cro} in C_6D_6, no meaningful $^{13}C\{^1H\}$ and $^{29}Si\{^1H\}$ NMR could be recorded. – UV-vis (C_6H_6, λ_{max}, nm): 821, 522. – Elemental analysis calcd (%) for $C_{120}H_{240}Al_4K_2O_{12}Si_{16}$ (M_r = 2510.71): C 57.41, H 9.64; found C 57.98, H 9.50.

2A^{cry} (15 mg, 6.0 µmol, 95%) from **2** (11 mg, 6.3 µmol, 1.0 eq), KC_8 (2.0 mg, 15 µmol, 2.4 eq.), benzene (0.5 mL), [2.2.2]cryptand (5.6 mg, 15 µmol, 2.4 eq.).
^{1}H NMR (500 MHz, C_6D_6) δ = 8.61 (s, 4H, **H**^{3}), 8.27 (d, $^3J_{H,H}$ = 19.2 Hz, 4H, **H**^{5}), 7.47 (s, 4H, **H**^{1}), 6.68 (d, $^3J_{H,H}$ = 19.2 Hz, 4H, **H**^{6}), 3.06 (s, 24H, **H**^{cry}), 2.95 (s, 24H, **H**^{cry}), 2.00 (s, 24H, **H**^{cry}), 0.53 (s, 144H, $Si(C\mathbf{H}_3)_3$), −0.06 (s, 8H, AlC**H**) ppm. – $^{29}Si\{^1H\}$ NMR (99 MHz, C_6D_6) δ = −3.4 ppm. – In the $^{13}C\{^1H\}$ NMR spectrum, the signals of the $AlBis_2$ unit were observed, but not those of the dbCOT system, see Figure S32 – UV-vis (C_6H_6, λ_{max}, nm): 963, 556, 523.

3A^{cro} (14 mg, 5.5 µmol, 82 %) from **3** (11 mg, 6.7 µmol, 1.0 eq.), KC_8 (2.0 mg, 15 µmol, 2.2 eq.), benzene (0.5 mL), Cy_2-18-crown-6 (5.6 mg, 15 µmol, 2.4 eq.).
^{1}H NMR (500 MHz, C_6D_6) δ = 8.52 (s, 4H, **H**^{3}), 7.44 (s, 4H, **H**^{1}), 3.62–2.92 (m, 36H, **H**^{cro}), 2.01–1.04 (m, 36H, **H**^{cro}), 0.55 (s, 144H, $Si(C\mathbf{H}_3)_3$), −0.19 (s, 8H, AlC**H**) ppm. $^{29}Si\{^1H\}$ NMR (99 MHz, C_6D_6) δ = −3.6 ppm. – No interpretable ^{13}C{1H} NMR spectra could be obtained. – UV-vis (C_6H_6, λ_{max}, nm): 673, 465.

3A^{cry} (16 mg, 6.3 µmol, 94 %) from **3** (11 mg, 6.7 µmol, 1.0 eq.), KC_8 (2.0 mg, 15 µmol, 2.2 eq.), benzene (0.5 mL), [2.2.2]cryptand (5.6 mg, 15 µmol, 2.2 eq).
^{1}H NMR (500 MHz, C_6D_6) δ = 8.62 (s, 4H, **H**^{3}), 7.42 (s, 4H, **H**^{1}), 3.04 (s, 24H, **H**^{cry}), 2.93 (s, 24H, **H**^{cry}), 1.97 (s, 24H, **H**^{cry}), 0.57 (s, 144H, $Si(C\mathbf{H}_3)_3$), −0.20 (s, 2H, AlC**H**) ppm. – $^{29}Si\{^1H\}$ NMR (99 MHz, C_6D_6) δ = −2.9. – In the $^{13}C\{^1H\}$ NMR spectrum, the signals of the $AlBis_2$ unit were observed, but not those of the dbCOT system, see Figure S44 – UV-vis (C_6H_6, λ_{max}, nm): 785, 490, 395. – Elemental analysis calcd (%) for $C_{116}H_{232}Al_4K_2N_4O_{12}Si_{16}$ (M_r = 2510.63): C 55.50, H 9.31, N 2.23; found C 55.23, H 9.55, N 1.97.

Preparation of $[\{K^+([2.2.2]cryptand)\}_2(\mathbf{4}^{2-})]$ (**4A**^{cry})

KC_8 (12 mg, 89 µmol, 2.5 eq.) was added to a solution of compound **4** (34 mg, 36 µmol, 1.0 eq.) in THF (0.5 mL). The solution immediately took on a deep dark purple colour. After 2 min, the suspension was filtered to remove graphite and the residue was washed with THF (0.3 mL). The resulting solution which contained **4A**^{THF} was analysed by NMR spectroscopy before adding [2.2.2]cryptand (37 mg, 97 µmol, 2.7 eq.). Immediately after addition, **4A**^{cry} (55 mg, 31 µmol, 86%) began to crystallise, so that no meaningful NMR spectroscopic analysis could be performed. The resulting single crystals were suitable for the determination of a solid-state structure by X-ray diffraction experiments.

4A^{THF}: ^{1}H NMR (500 MHz, THF-d_8) δ = 8.07 (s, 4H, **H**^{3}), 7.11 (s, 4H, **H**^{1}), 0.29 (s, 36H, $Sn(C\mathbf{H}_3)_3$) ppm. – $^{13}C\{^1H\}$ NMR (126 MHz, THF-d_8) δ = 141.1 (**C**^{3}), 114.4 (**C**^{5}), 108.5 (**C**^{2}), 103.4 (**C**^{4}), 99.0 (**C**^{1}), 86.3 (**C**^{6}), −8.5 ($Sn(\mathbf{C}H_3)_3$). – $^{119}Sn\{^1H\}$ NMR (187 MHz, THF-d_8) δ = −74.0 ppm.

4A^{cry}: UV-vis (C_6H_6, λ_{max}, nm): 974, 560, 530.
Elemental analysis calcd (%) for $C_{72}H_{116}K_2N_4O_{12}Sn_4$ (M_r = 1782.77): C 48.51, H 6.56, N 3.14; found C 48.48. H 6.51, N 3.10.

Investigations into the reduction of fluorinated PLAs $[(C_6F_5)_2BCHCH]_4$–, $[(C_2F_5)_2SbCC]_4$–, $[F_3CSeCC]_4$–dbCOT

The fluorinated PLAs were combined with KC_8 in THF or benzene in the same way as described above. Furthermore, the reduction was carried out with KC_8 with the prior addition of [2.2.2]cryptand. In all cases, there was no colour change and the characterizations still showed only the presence of the neutral systems.

NMR-Investigations

NMR-investigation of redox switchability with *o*-chloranil

To investigate the ability to oxidise the reduced compounds with *o*-chloranil, a stoichiometric amount of *o*-chloranil was added to NMR samples of the reduced systems; the addition caused the solutions to lighten up and orange-coloured solutions were obtained. The colour can be partly attributed to the dipotassium alcoholate formed. The solutions obtained were analysed in more detail using NMR spectroscopy. **1A**^{THF} and **4A**^{THF} could be completely oxidised, **2**K_2 and **3**K_2 showed decomposition.

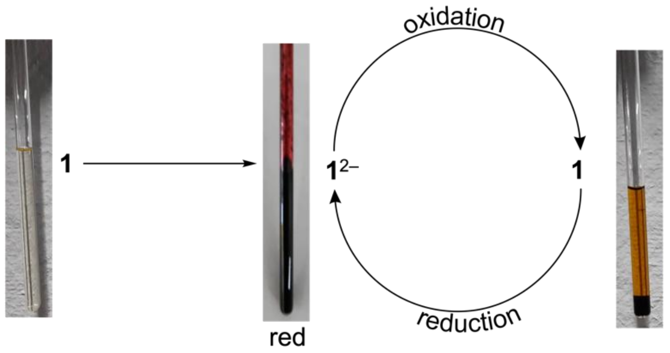

Figure S1. Illustration of the colour change during the reduction of $[Cy_2BCHCH]_4$-dbCOT **1** with KC_8 and the subsequent oxidation with *o*-chloranil.

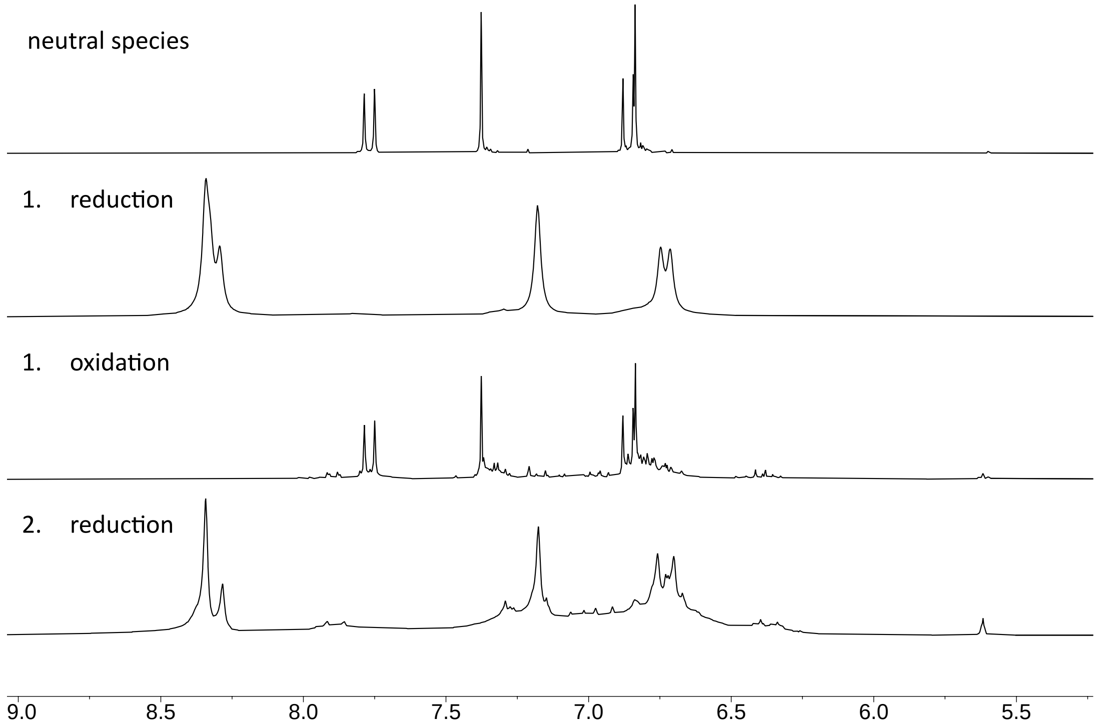

Figure S2. NMR spectra of the reduction of $[Cy_2BCHCH]_4$-dbCOT **1** with KC_8 and the subsequent oxidation with *o*-chloranil, in THF-d_8 at 298 K, 500 MHz. However, some additionally observed signals indicate a partial decomposition of the compound by the strong oxidising agent *o*-chloranil, whose reduced form could also react as a potent poly Lewis base with the neutral PLA.

NMR-investigation of redox switchability with iodine

To investigate the ability to oxidise the reduced compounds using iodine, a stoichiometric amount of an iodine-benzene (for **2A** and **3A**) or iodine-THF (for **1A**^{THF} and **4A**^{THF}) solution was added to NMR samples of the reduced systems; the addition caused the solutions to decolourise and colourless to pale yellow suspensions were obtained. After a few minutes, the colourless solid separated and the resulting supernatant solutions could be examined in more detail using NMR spectroscopy. All reduced compounds could be oxidised in this way.

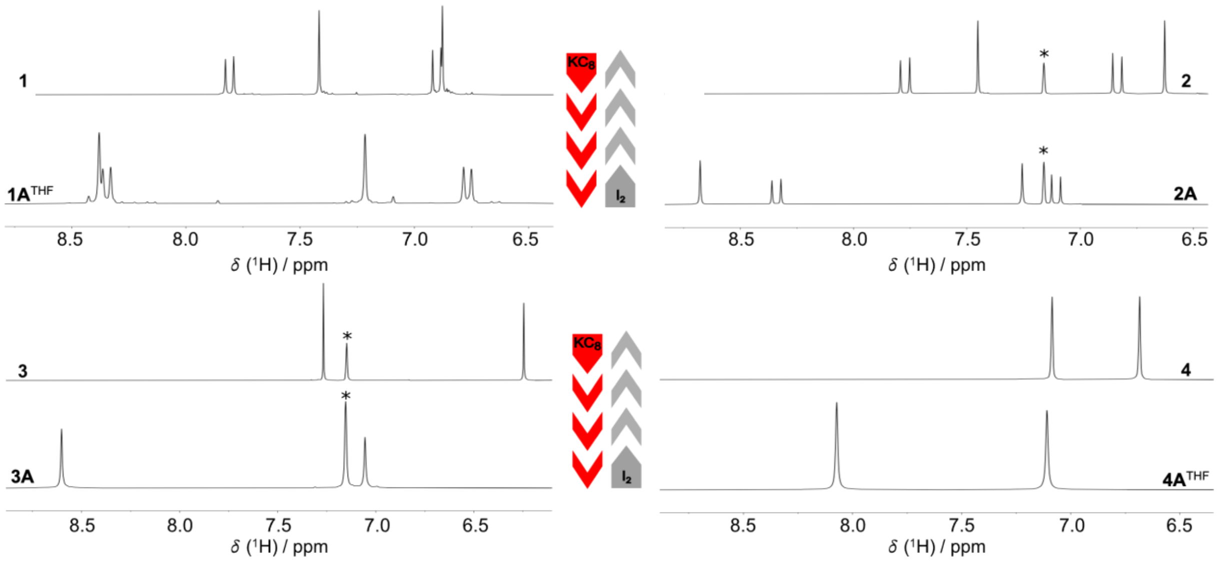

Figure S3. NMR spectra of the reduction **1**, **2**, **3** and **4** with KC_8 and the subsequent oxidation with iodine, in THF-d_8 (**1**, **3**) or C_6D_6 (**2**, **3**) at 298 K, 500 MHz.

NMR-investigations into the switchability of PMe_3 adducts

PLA **1** and **3** were mixed with an excess of PMe_3 in a Young-NMR tube before being held for 1 h at 80 °C and 0.003 mbar. After dissolving (**1**·4PMe_3 in THF-d_8 and **3**·4PMe_3 in C_6D_6) the residue, NMR spectroscopy was used to determine the presence of the 1:4 adduct without excess PMe_3. KC_8 (2.2 eq.) was added to the resulting solutions, causing them to turn from red to purple. After examination by NMR spectroscopy, the solvents were freed of all volatile components at room temperature and after further drying of the residue for 15 min at room temperature at 0.003 mbar, the residues were dissolved again and examined by NMR spectroscopy. A stoichiometric amount of iodine was added to the solutions obtained and the resulting solutions were analysed by NMR spectroscopy. To investigate the effects of the potassium cations on the shift of PMe_3, [2.2.2]cryptand was added to the reduced adduct in a separate experiment and the mixture was analysed.

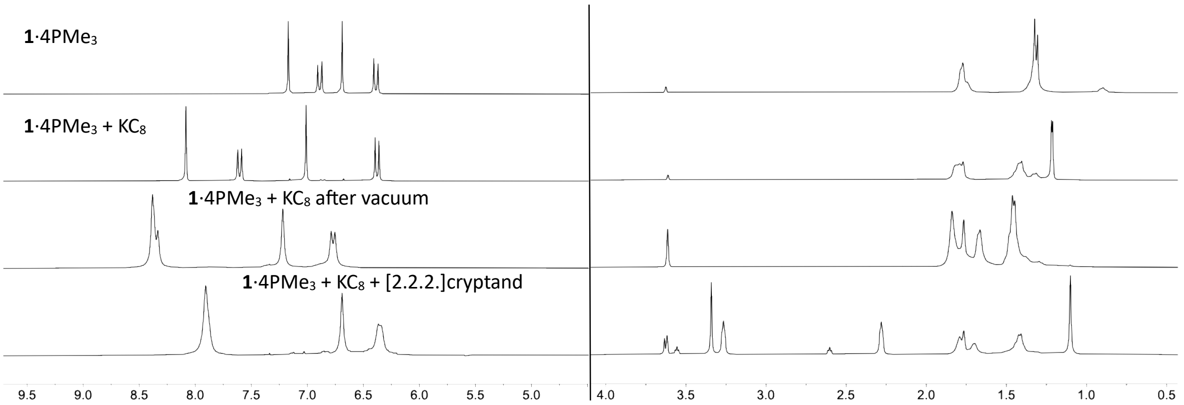

Figure S4. ^{1}H NMR spectra of the redox switching experiments with **1**·4PMe_3, after addition of KC_8, after addition of KC_8 and drying in vacuum and after reduction with KC_8 and addition of [2.2.2]cryptand in THF-d_8 at 298 K, 500 MHz.

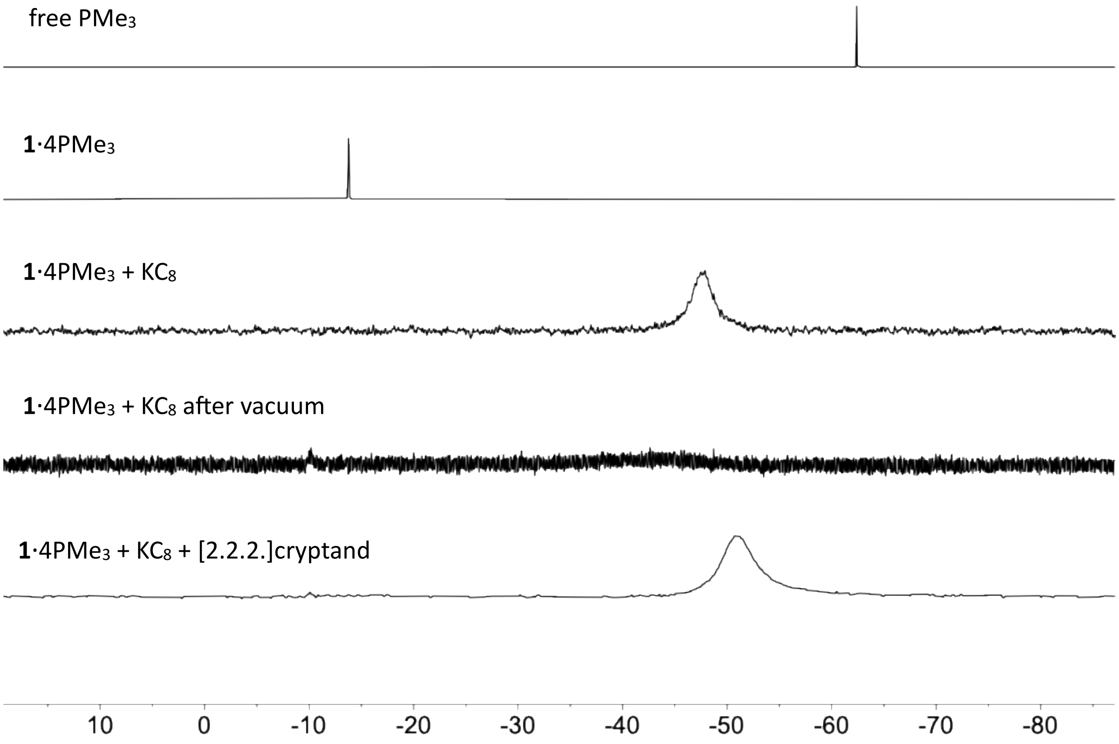

Figure S5. ^{31}P NMR spectra of the redox switching experiments with **1**·4PMe$_3$, after addition of KC$_8$, after addition of KC$_8$ and drying in vacuum and after reduction with KC$_8$ and addition of [2.2.2]cryptand in THF-d_8 at 298 K, 202 MHz.

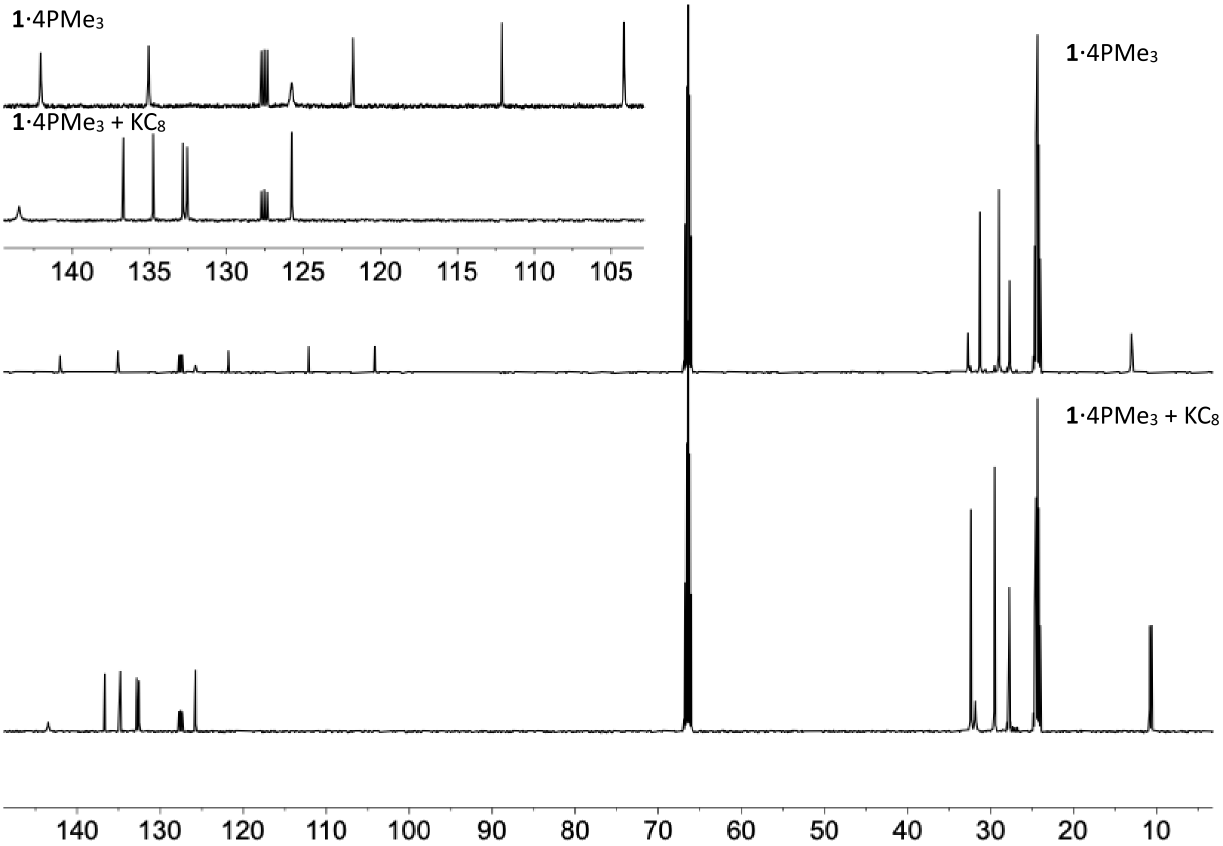

Figure S6. $^{13}C\{^1H\}$ NMR spectra of the redox switching experiments with **1**·4PMe$_3$, in THF-d_8 at 298 K, 125 MHz.

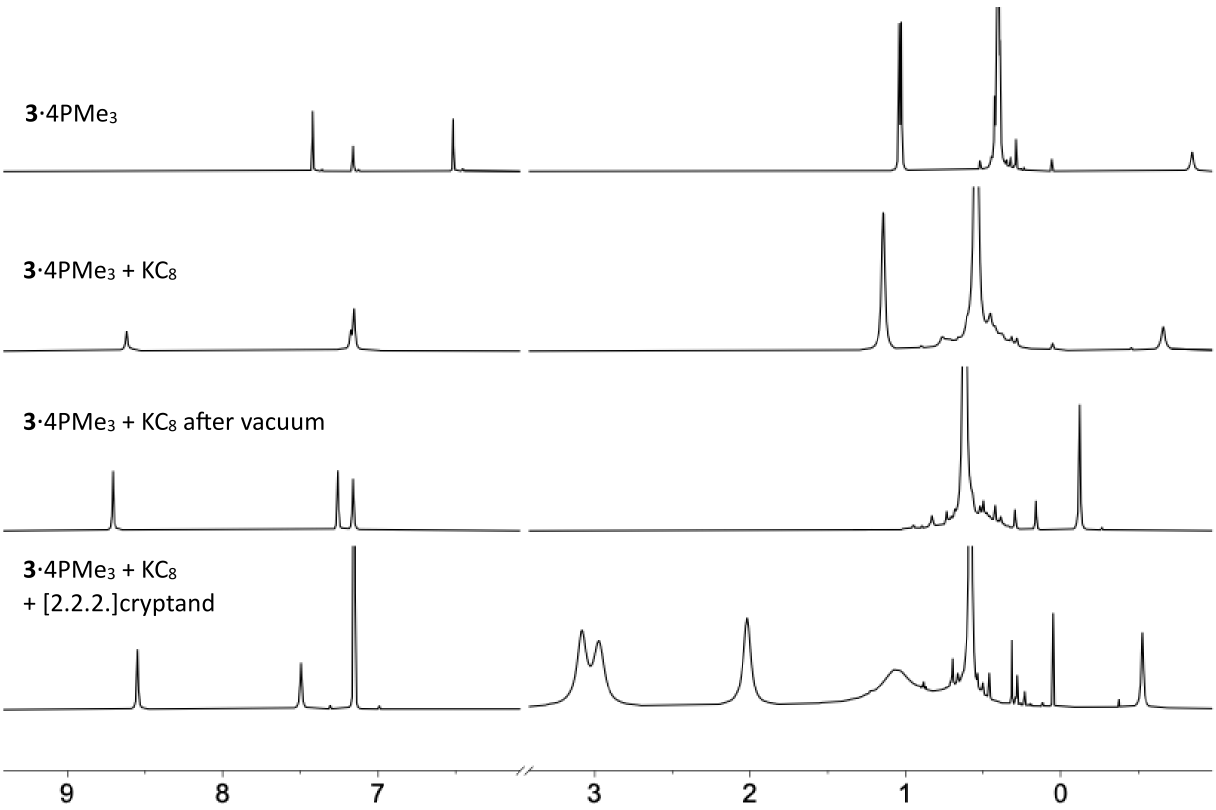

Figure S7. ^{1}H NMR spectra of the redox switching experiments with **3**·4PMe$_3$, in C_6D_6 at 298 K, 500 MHz.

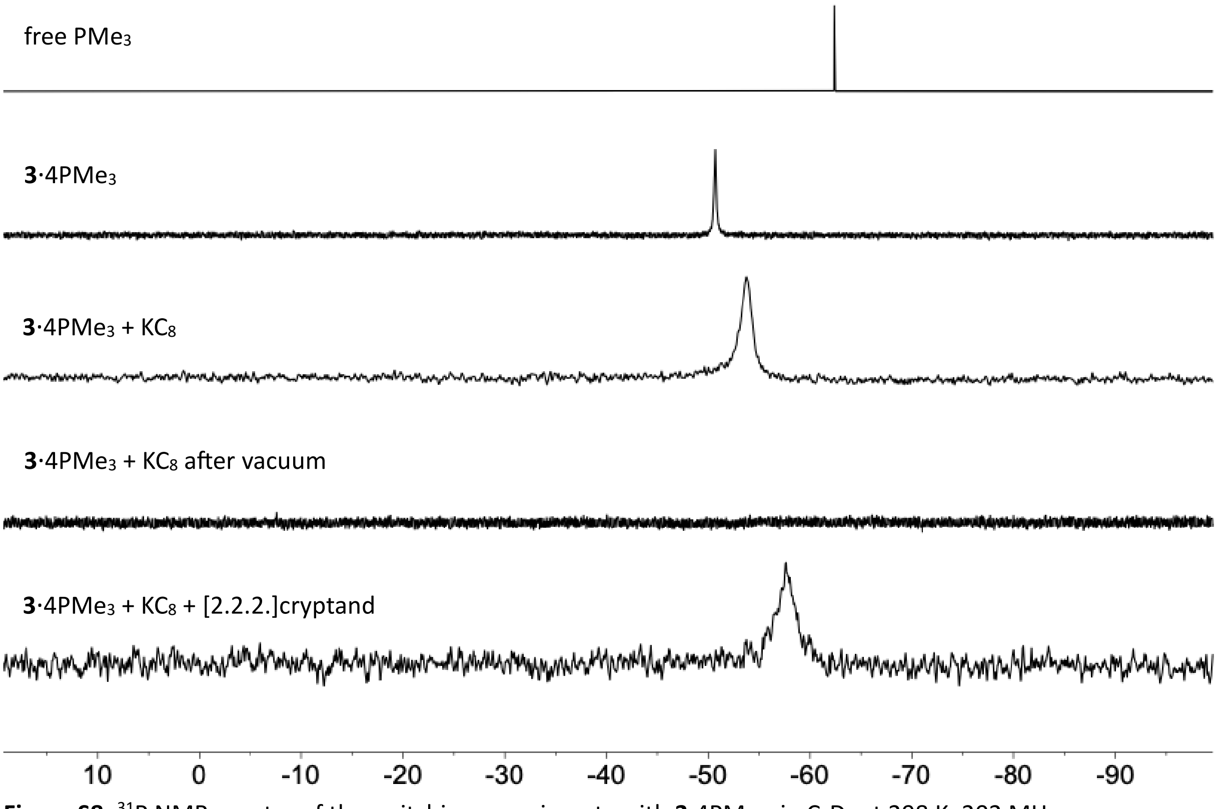

Figure S8. ^{31}P NMR spectra of the switching experiments with **3**·4PMe$_3$, in C_6D_6 at 298 K, 202 MHz.

NMR investigations on the switchability of [**1**·2P[2]]$_2$

In a Young NMR tube, bis((dimethylphosphanyl)methyl)dimethylsilane was added to **1** in THF-*d*$_8$ and subsequently analysed by NMR spectroscopy before KC_8 was added and the now deep red mixture was analysed again by NMR spectroscopy. The addition of iodine led to decolouration of the mixture and a further analysis using NMR spectroscopy was carried out.

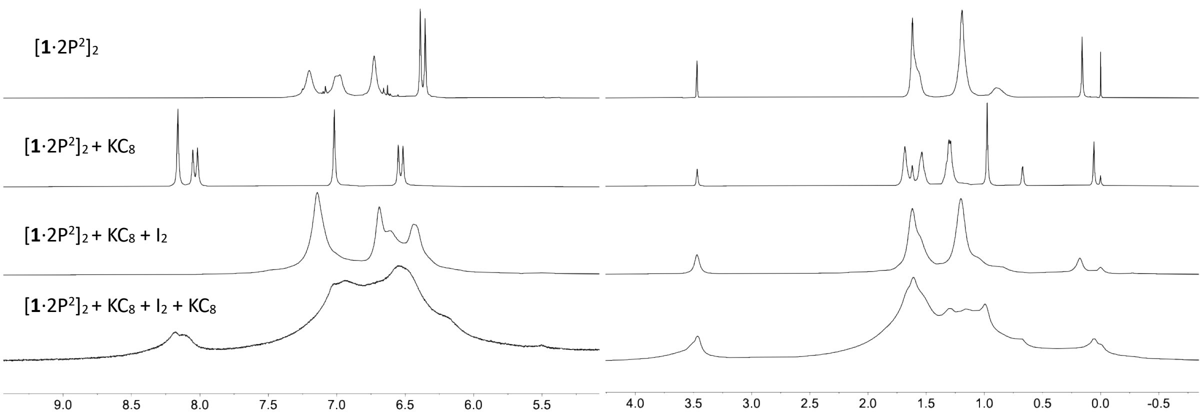

Figure S9. ^{1}H NMR spectra of the redox switching experiments with [**1**·2P[2]]$_2$, in THF-*d*$_8$ at 298 K, 500 MHz.

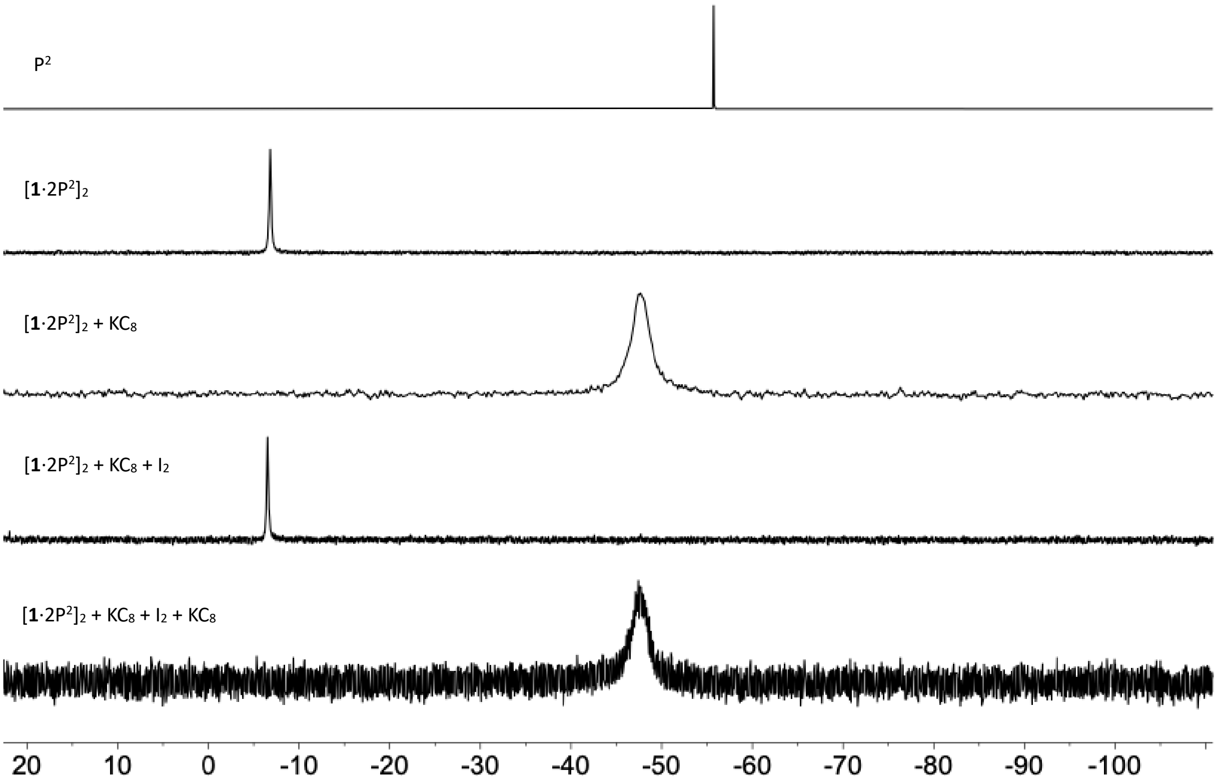

Figure S10. ^{31}P NMR spectra of the switching experiments with [**1**·2P[2]]$_2$, in THF-*d*$_8$ at 298 K, 202 MHz.

NMR investigations on the reduction of [**3**·P[4]]$_n$

In an Young NMR tube 4,7-bis((dimethylphosphanyl)methyl)-2,4,7,9-tetramethyl-2,9-diphospha-4,7-disiladecane (P[4]) was added to $[Bis_2AlCC]_4$-dbCOT **1** in C_6D_6 and the resulting mixture was analysed by NMR spectroscopy before KC_8 was added to the mixture and the resulting suspension was filtered into a fresh Young NMR tube after heating to reflux for 1 h. The resulting purple-coloured solution was analysed by NMR spectroscopy. An attempt was made to obtain suitable single crystals from the solution by concentrating it, but as this was unsuccessful, the residue was dissolved in C_6D_6 and Cy_2-18-crown-6 was added. The resulting mixture was analysed by NMR

spectroscopy before single crystals suitable for X-ray scattering experiments were obtained within 9 weeks without further concentration.
In a separate experiment, the reduced species was oxidised by adding a stoichiometric amount of iodine.

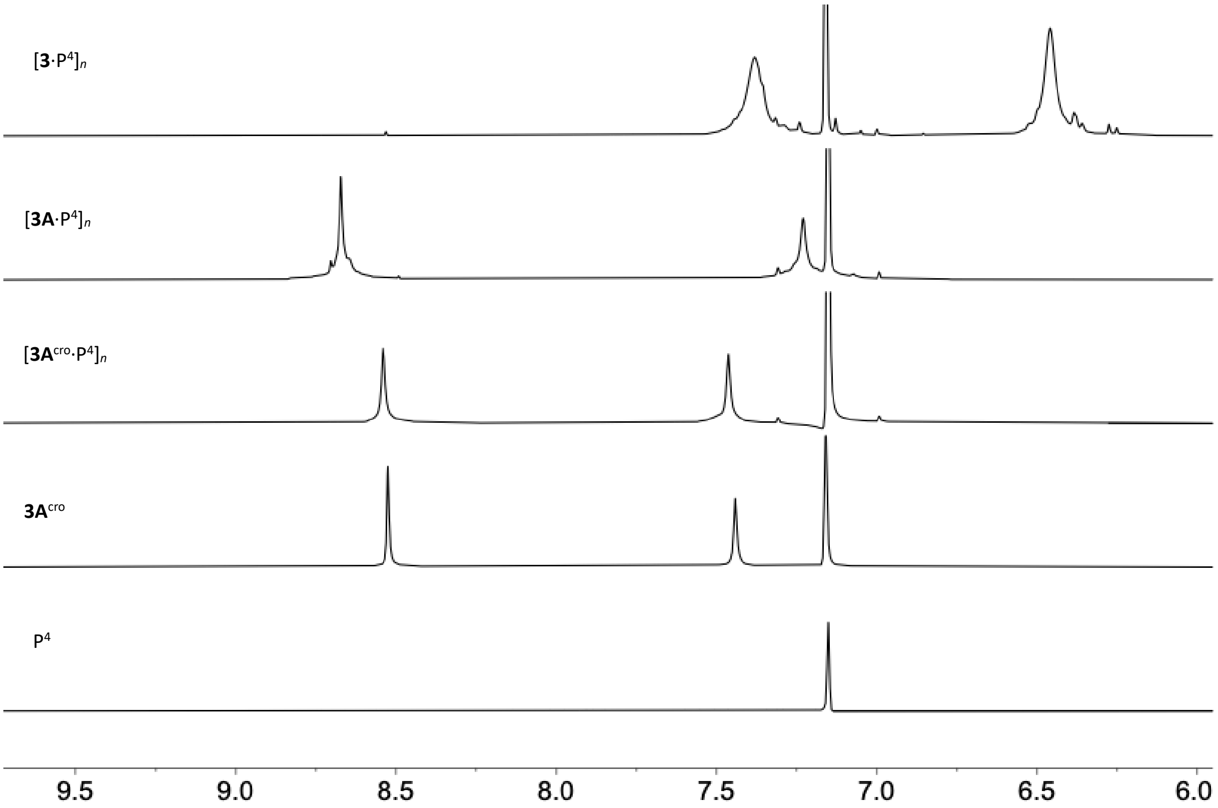

Figure S11. ^{1}H NMR spectra of the reduction experiments of [**3**·P4]*n*, in C_6D_6 at 298 K, 500 MHz.

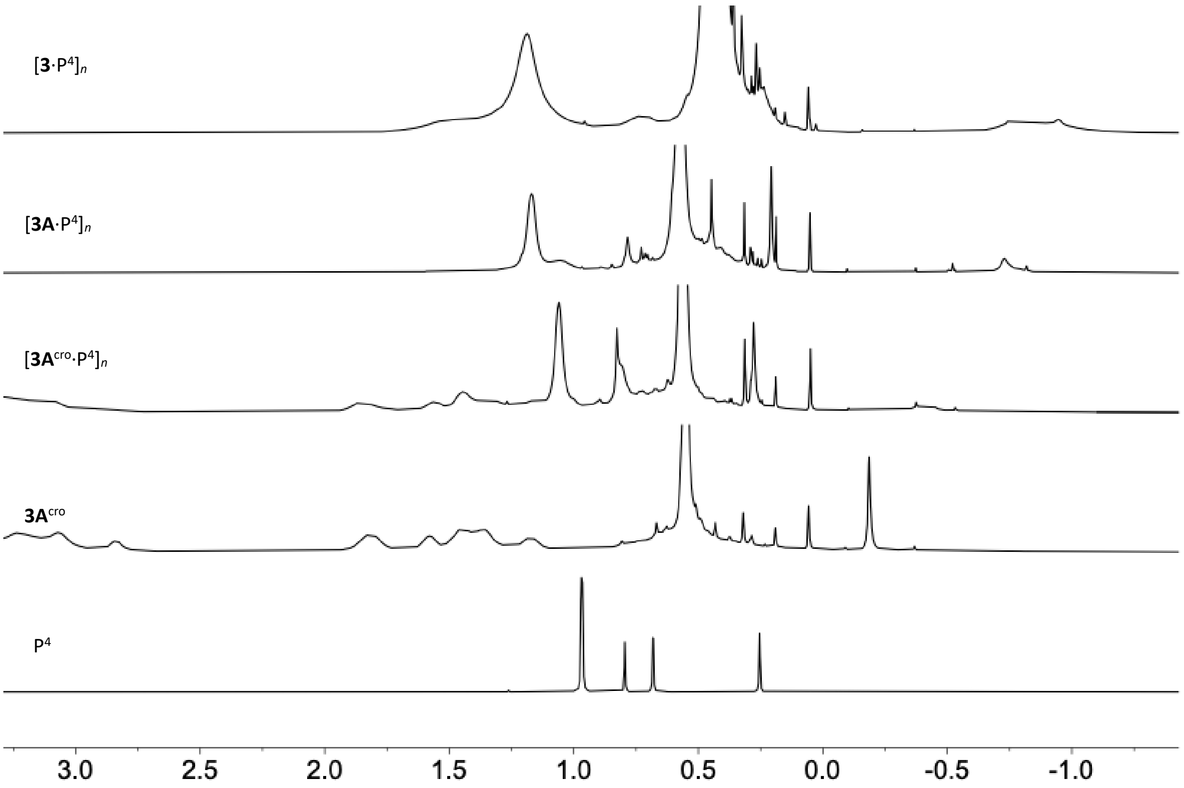

Figure S12. ^{1}H NMR spectra of the reduction experiments of [**3**·P4]*n*, in C_6D_6 at 298 K, 500 MHz.

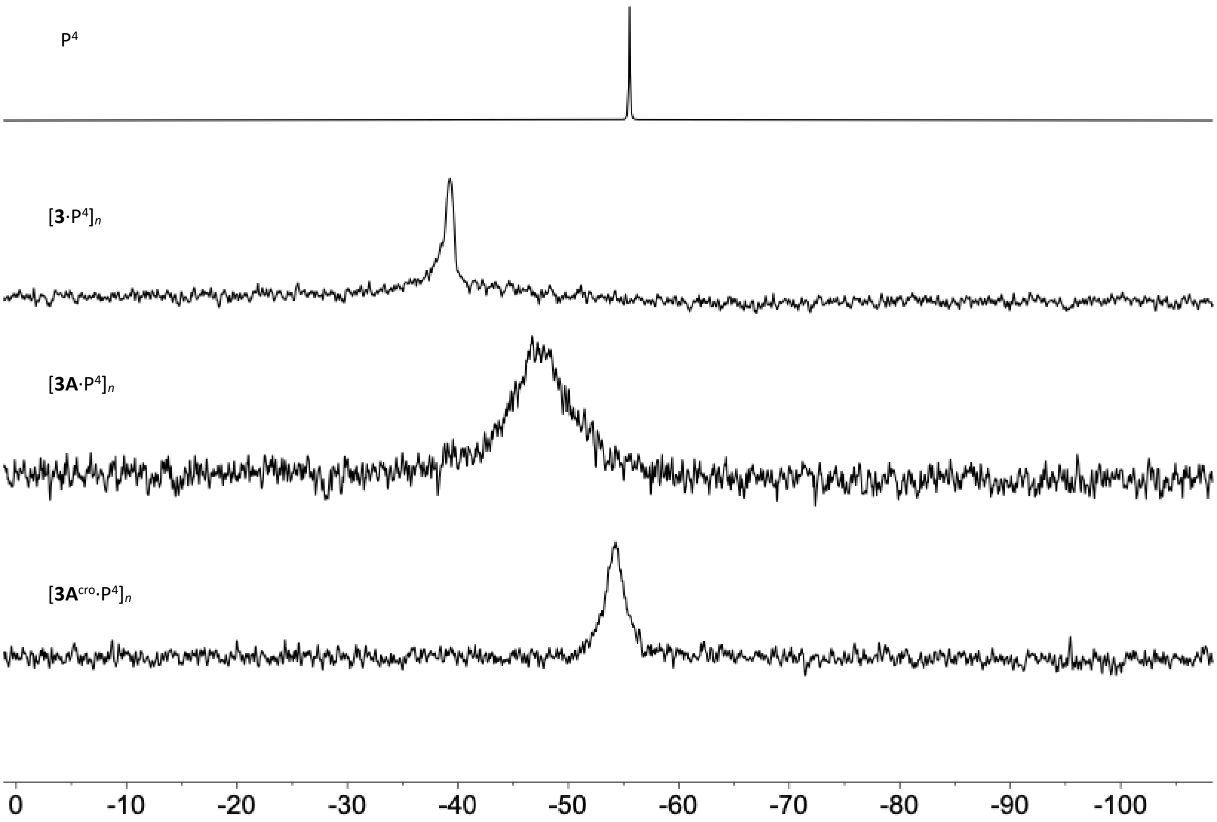

Figure S13. ^{31}P NMR spectra of the reduction experiments of [**3**·P^4]$_n$, in C_6D_6 at 298 K, 202 MHz.

NMR Spectra

Compound $\mathbf{1A^{THF}}$

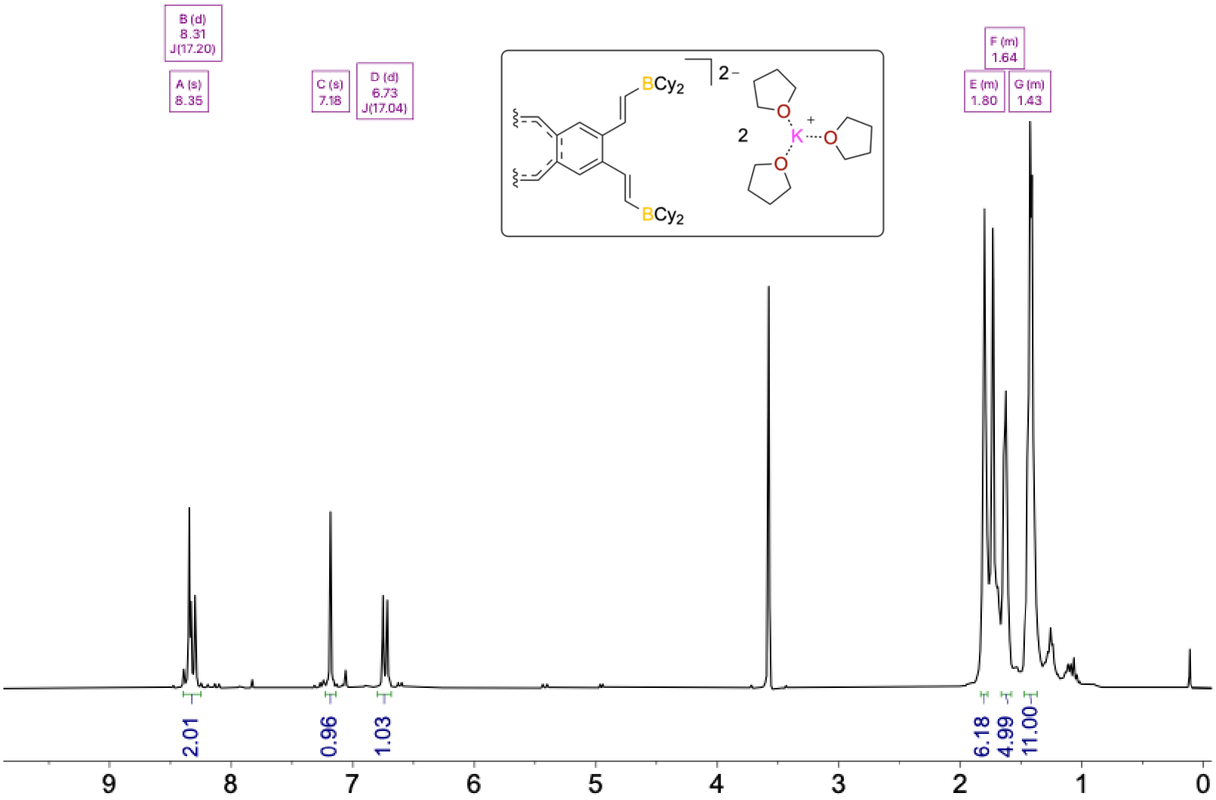

Figure S14. ^{1}H NMR spectrum of $\mathbf{1A}^{THF}$ in THF-d_8 at 298 K, 500 MHz.

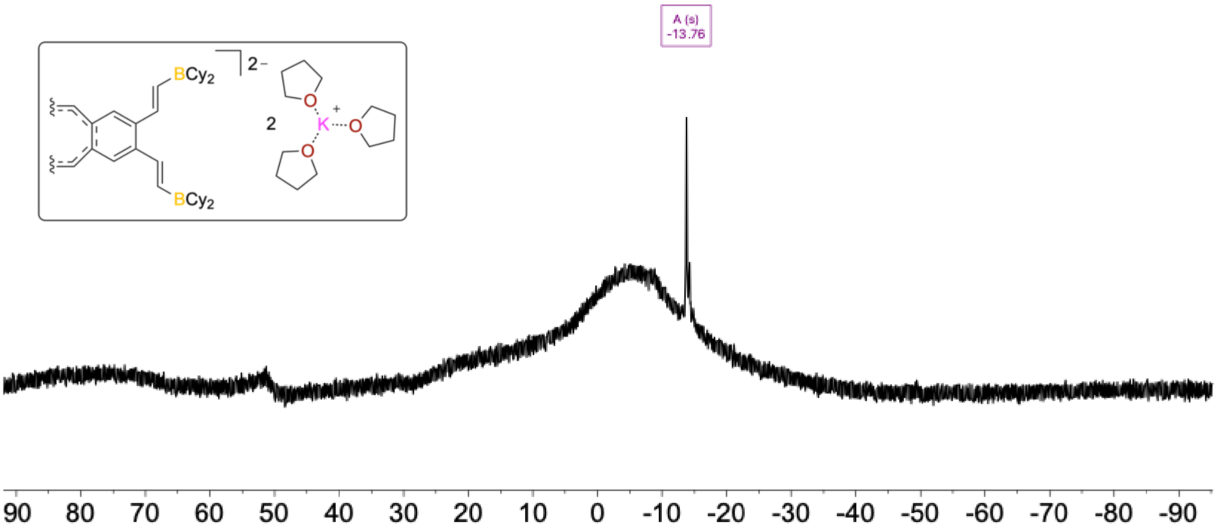

Figure S15. ^{11}B NMR spectrum of $\mathbf{1A}^{THF}$ in THF-d_8 at 298 K, 160 MHz.

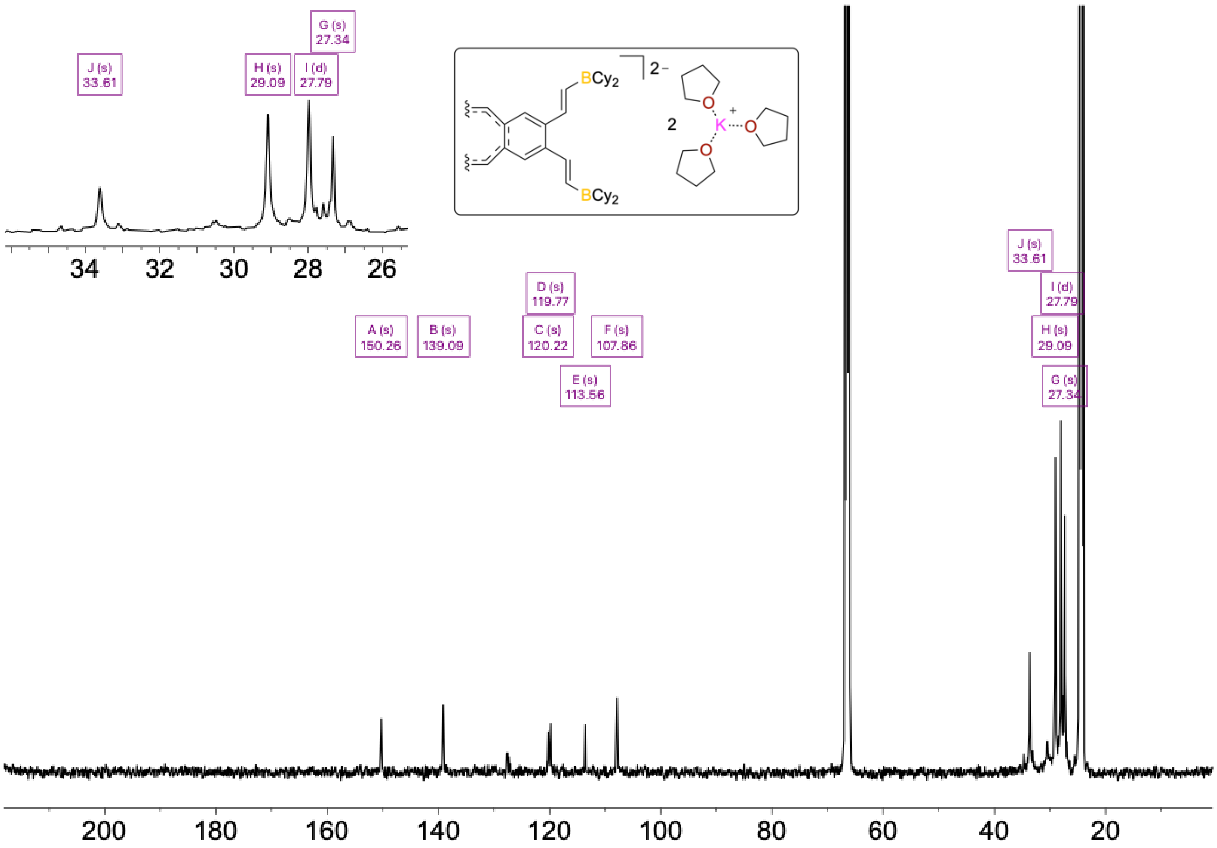

Figure S16. $^{13}C\{^1H\}$ NMR spectrum of **1A**^{THF} in THF-d_8 at 298 K, 125 MHz.

Compound **1A**^{cro}

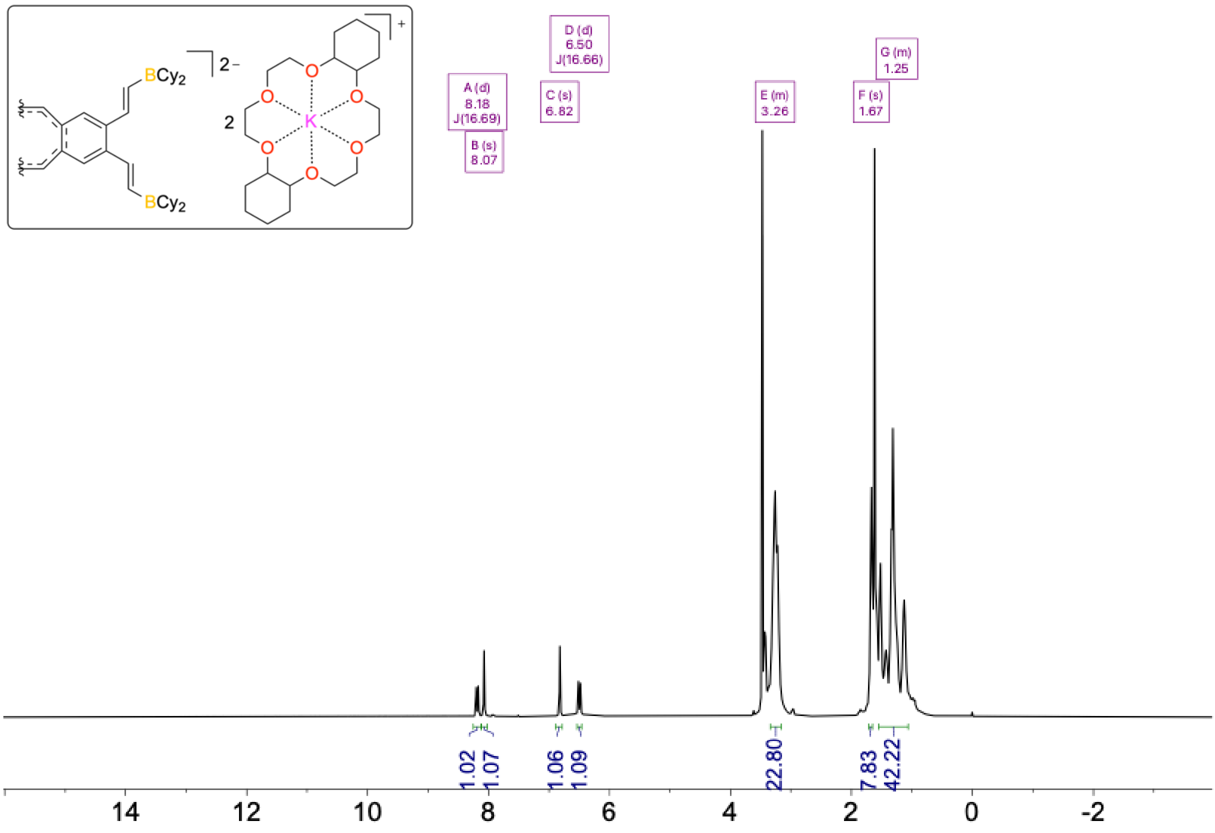

Figure S17. ^1H NMR spectrum of **1A**^{cro} in THF-d_8 at 298 K, 500 MHz.

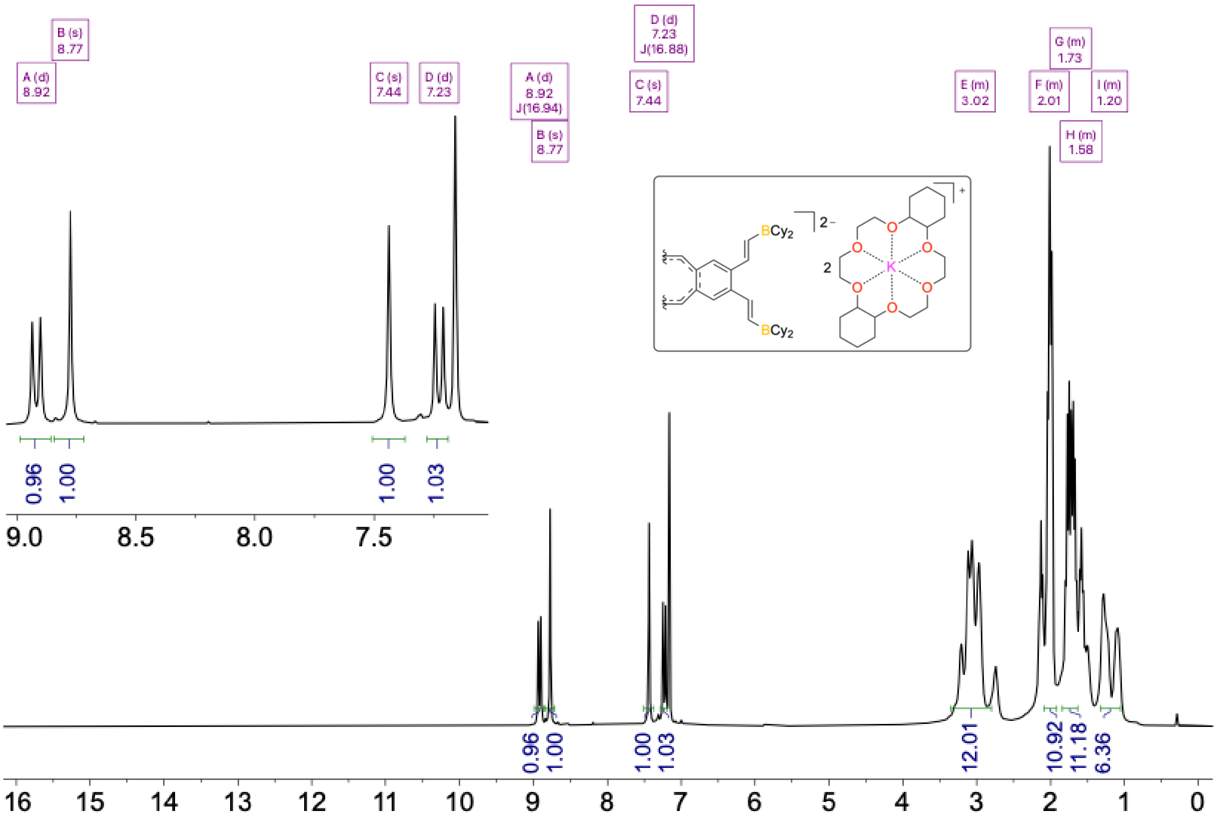

Figure S18. ^{1}H NMR spectrum of **1A**^{cro} in C_6D_6 at 298 K, 500 MHz.

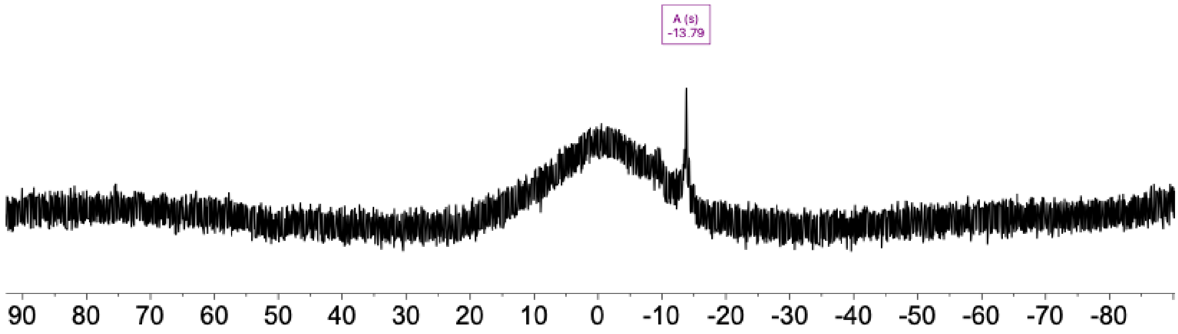

Figure S19a. ^{11}B NMR spectrum of **1A**^{cro} in THF-d_8 at 298 K, 160 MHz.

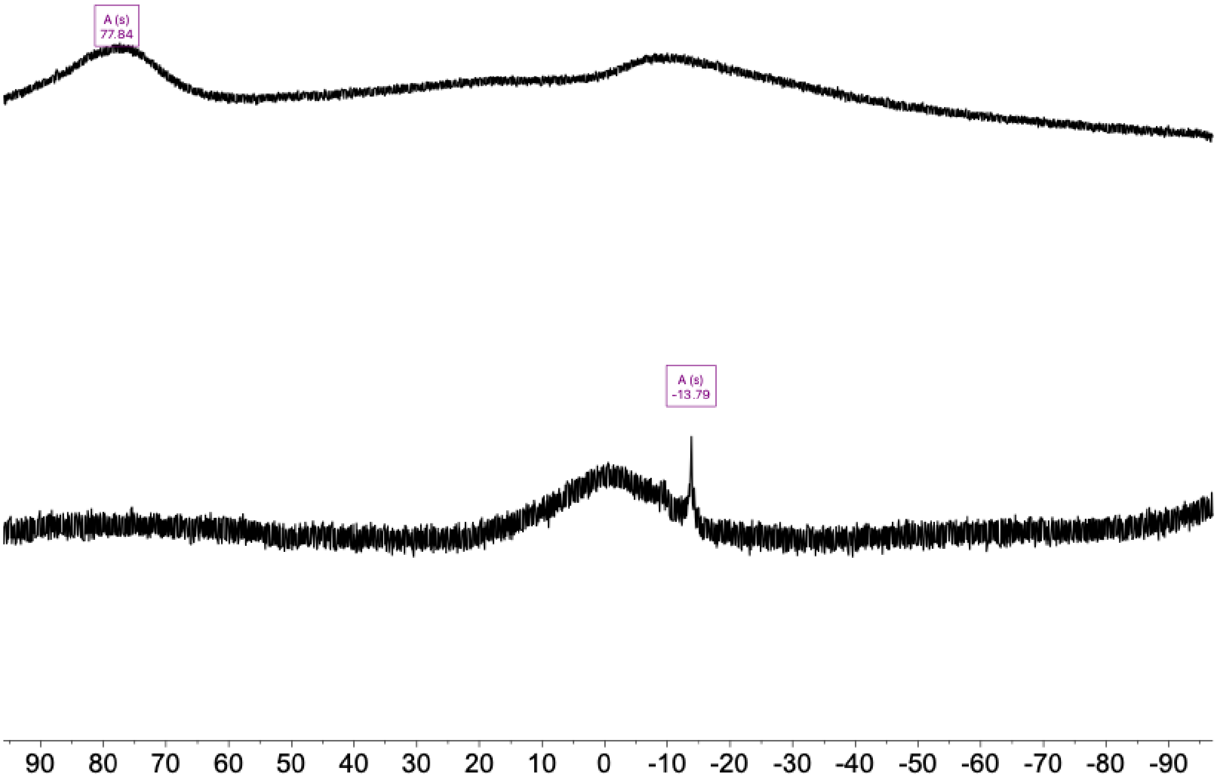

Figure S19b. ^{11}B NMR spectra of **1** (above) and **1A**^{cro} (below) in THF-d_8 at 298 K, 160 MHz.

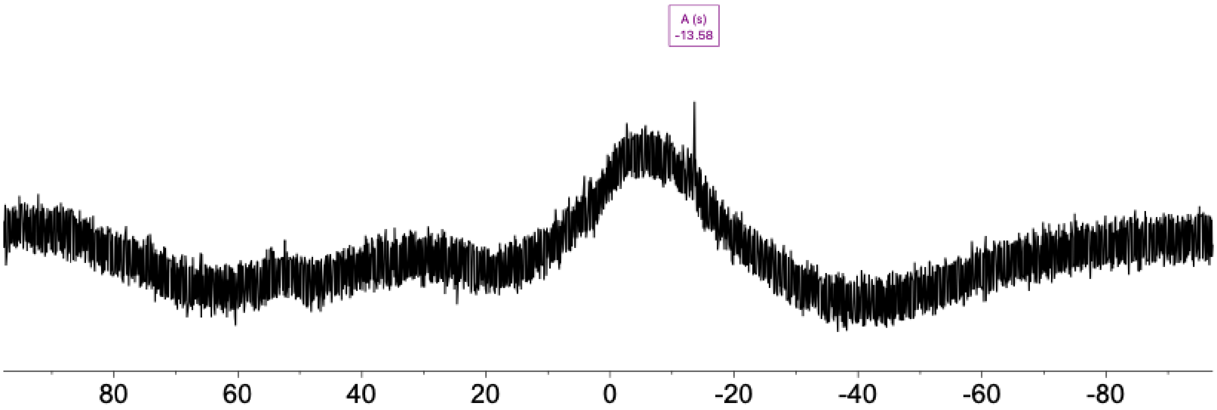

Figure S20. ^{11}B NMR spectrum of **1A**^{cro} in C_6D_6 at 298 K, 160 MHz.

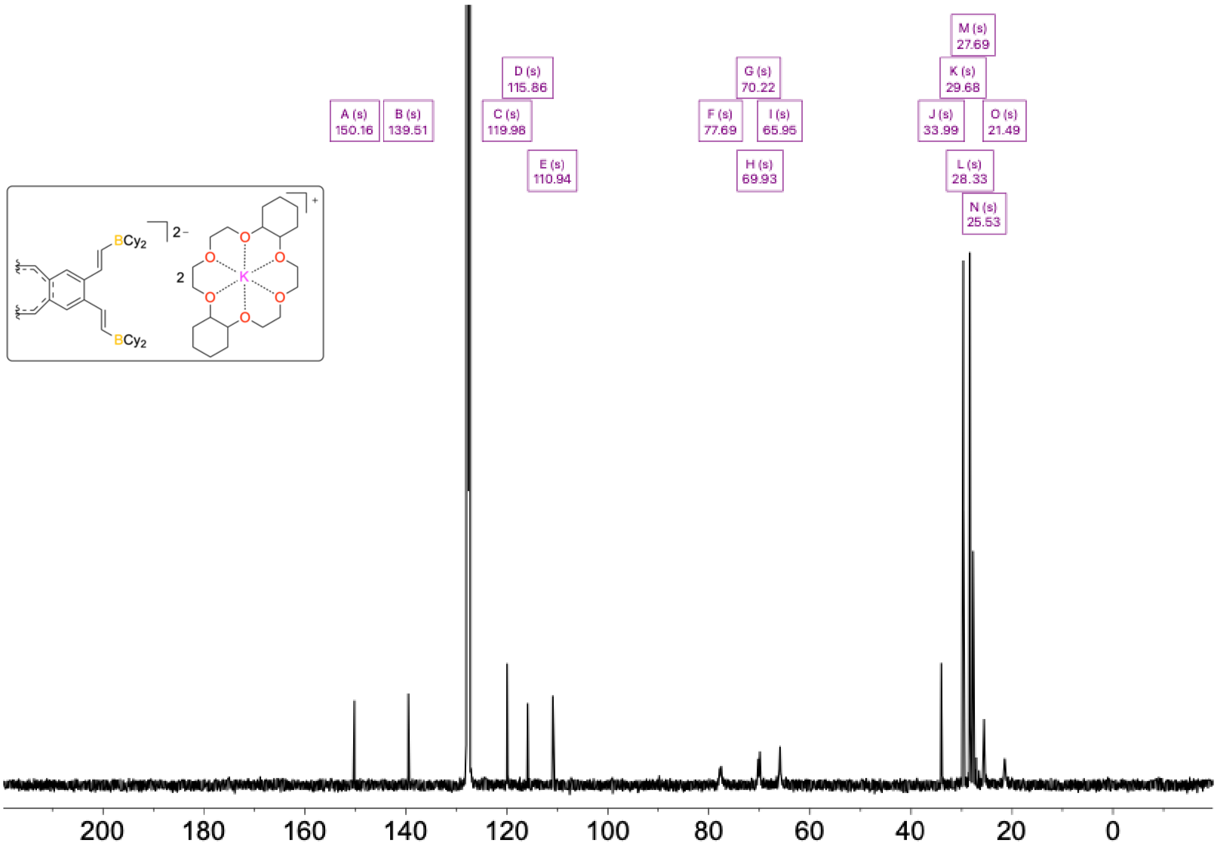

Figure S21. $^{13}C\{^1H\}$ NMR spectrum of **1A**^{cro} in C_6D_6at 298 K, 125 MHz.

Compound **1A**^{cry}

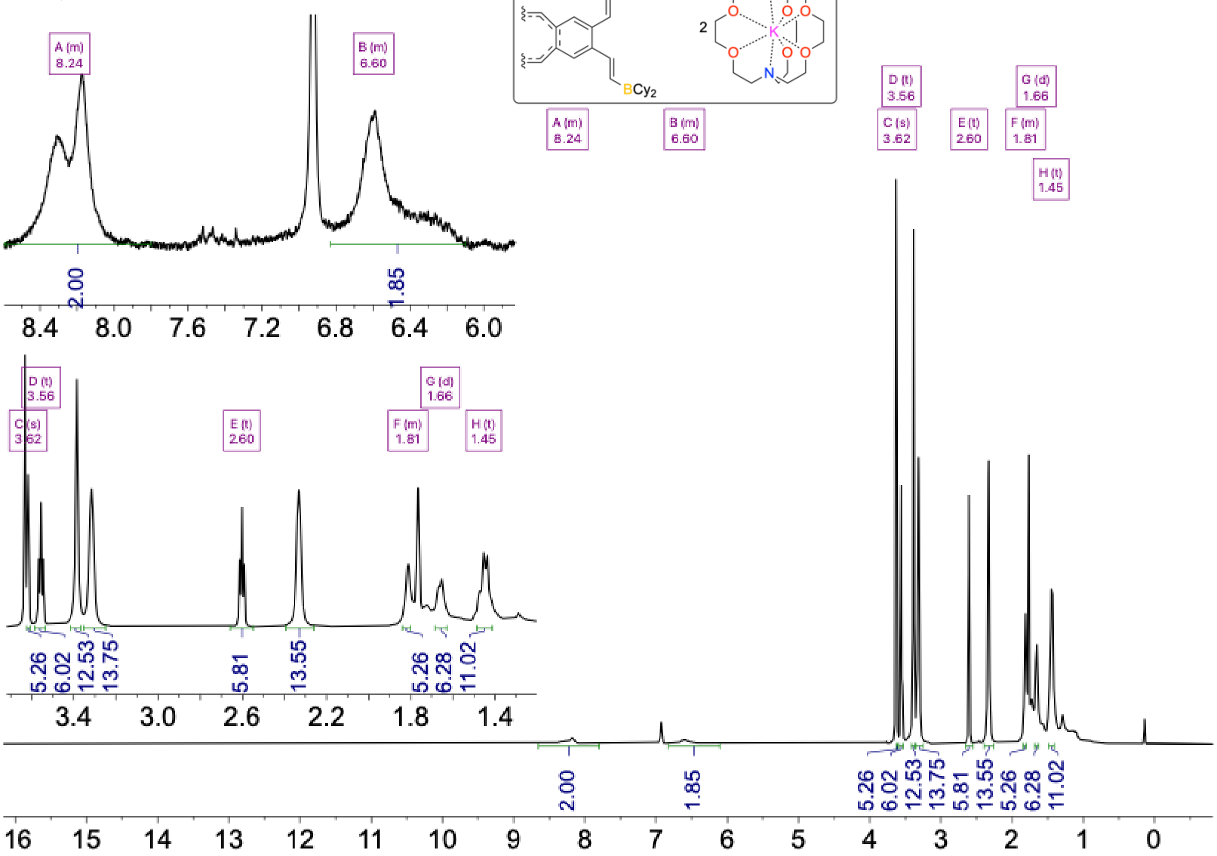

Figure S22. ^1H NMR spectrum of **1A**^{cry} with ex. [2.2.2]cryptand in THF-d_8 at 298 K, 500 MHz.

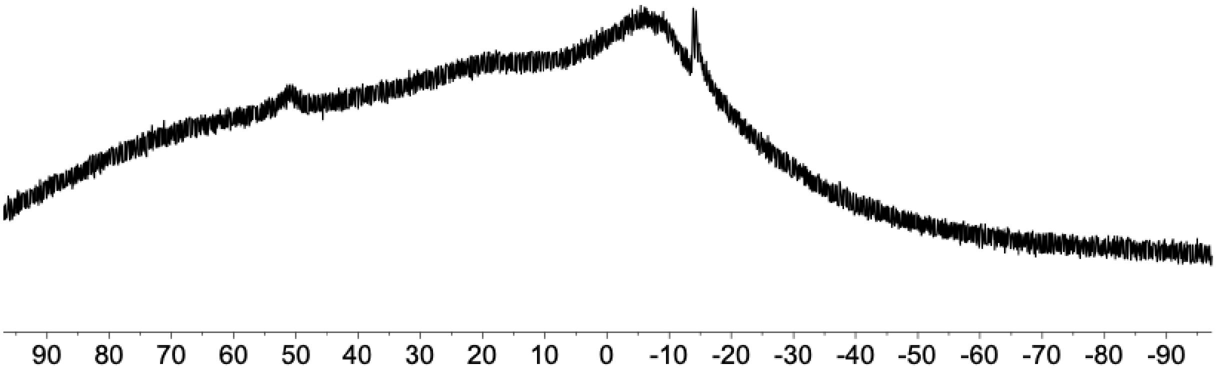

Figure S23. ^{11}B NMR spectrum of **1A**^{cro} in THF-d_8 at 298 K, 160 MHz.

Compound **2A**^{THF}

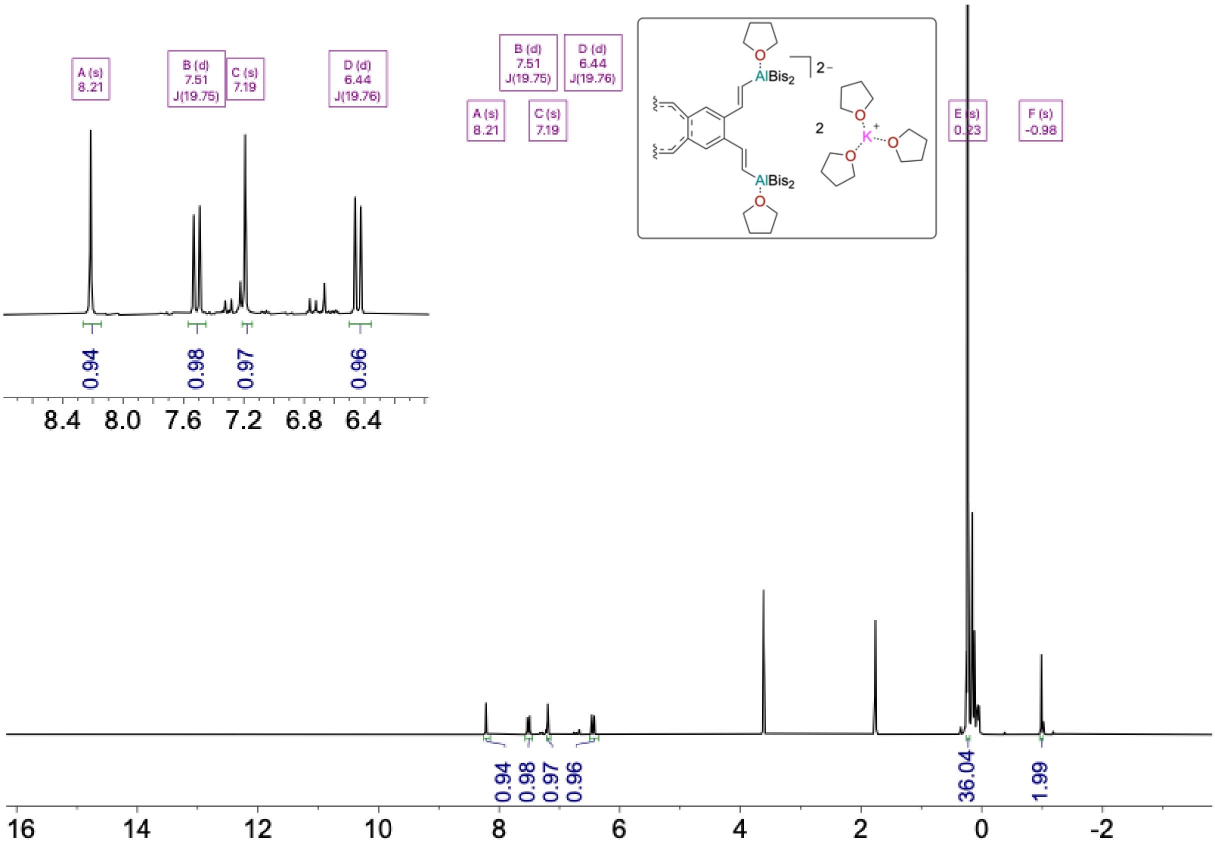

Figure S24. ^{1}H NMR spectrum of **2A**^{THF} in THF-d_8 at 298 K, 500 MHz.

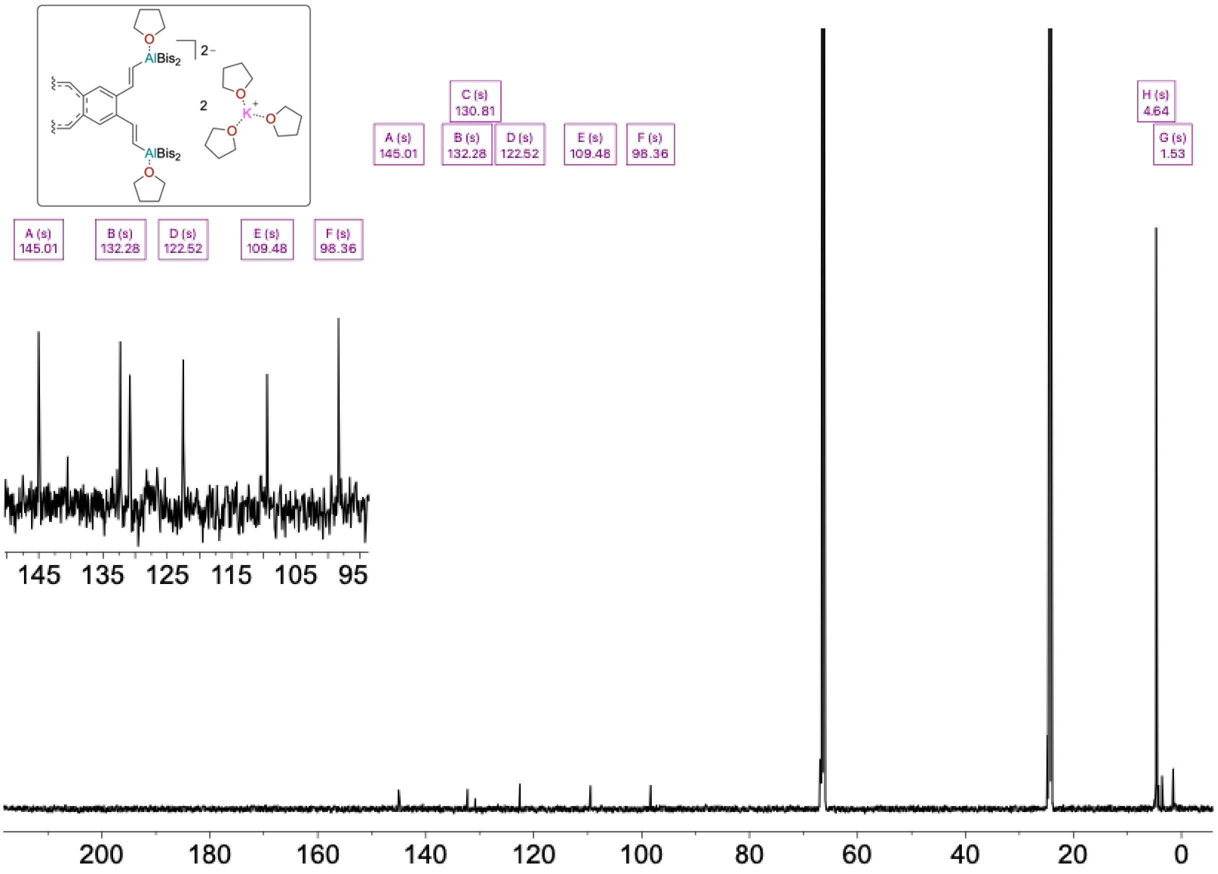

Figure S25. $^{13}C\{^1H\}$ NMR spectrum of **2A**^{THF} in THF-d_8 at 298 K, 125 MHz.

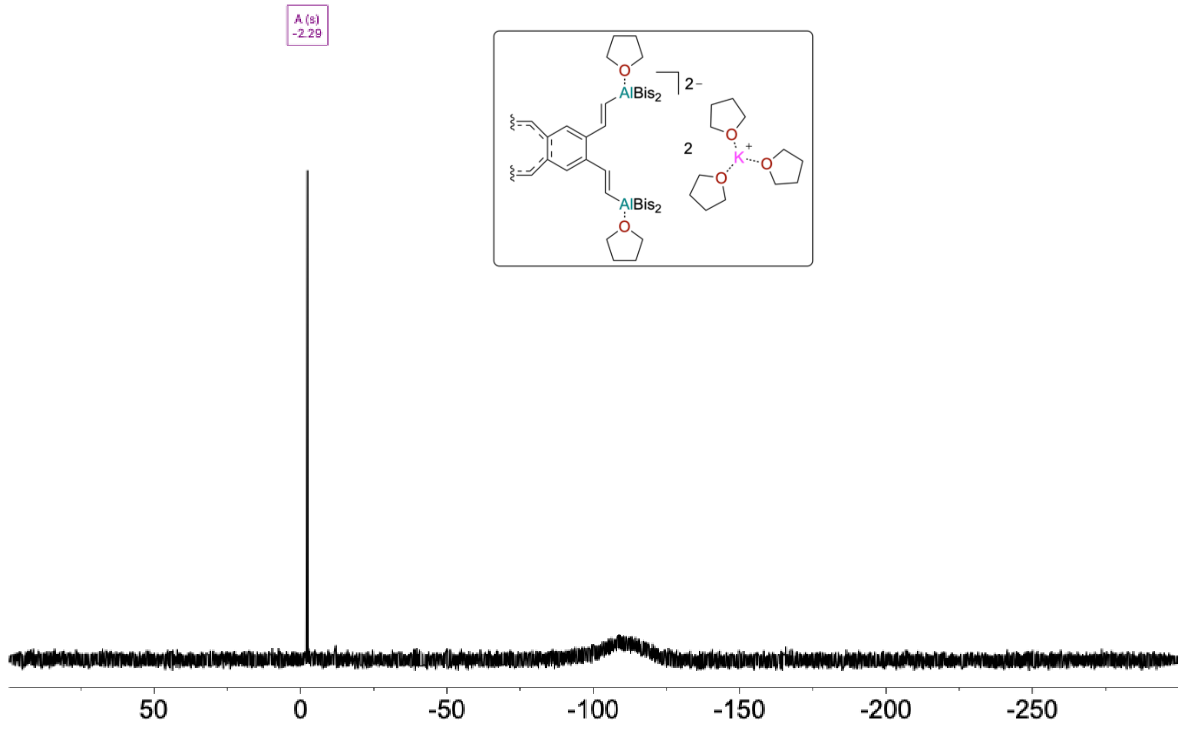

Figure S26. $^{29}Si\{^1H\}$ NMR spectrum of **2A**^{THF} in THF-d_8 at 298 K, 99 MHz.

Compound **2A**

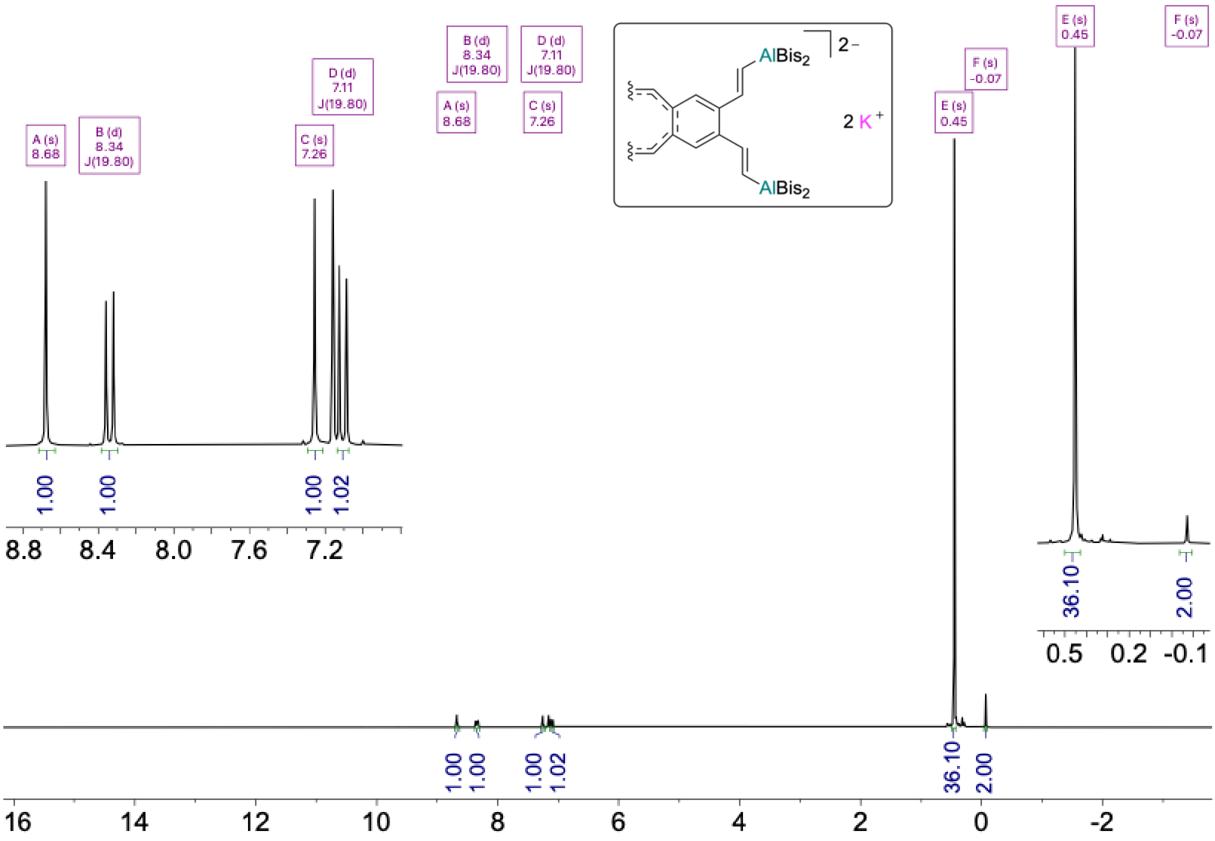

Figure S27. ^{1}H NMR spectrum of **2A** in C_6D_6 at 298 K, 500 MHz.

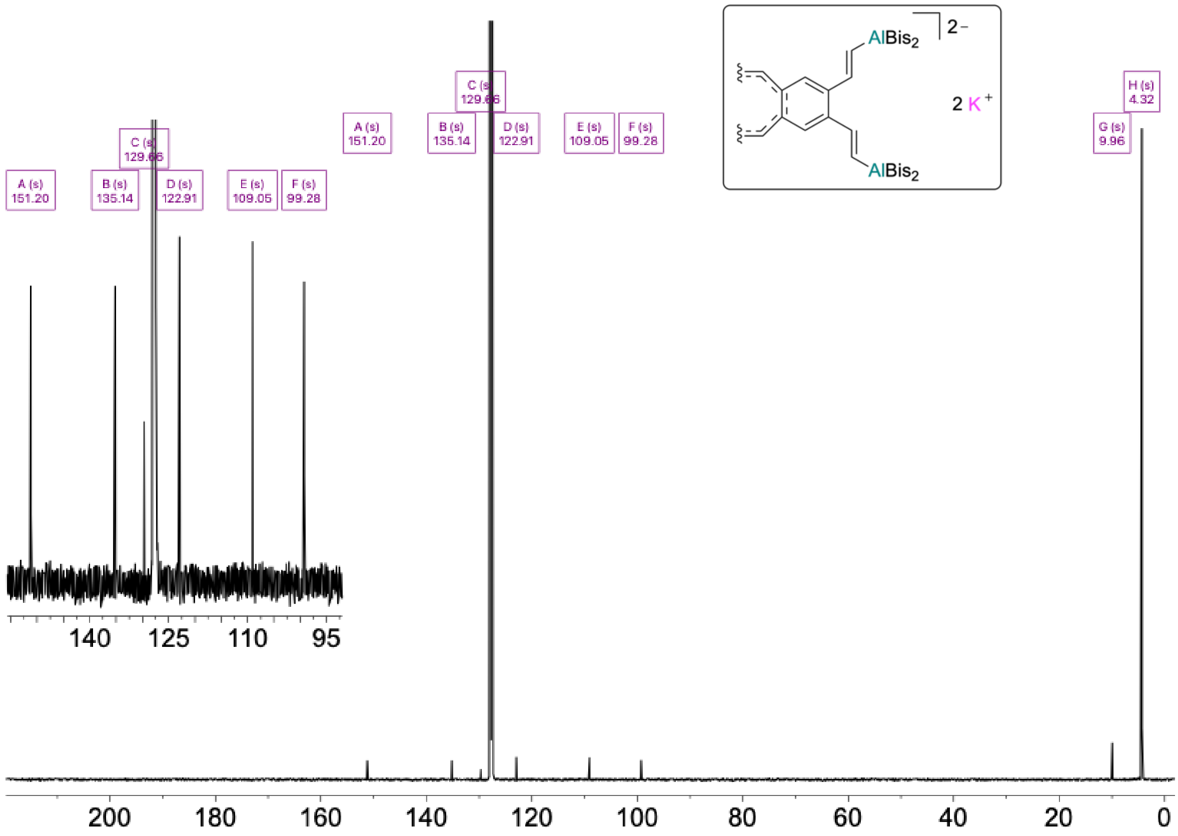

Figure S28. $^{13}C\{^1H\}$ NMR spectrum of **2A** in C_6D_6 at 298 K, 125 MHz.

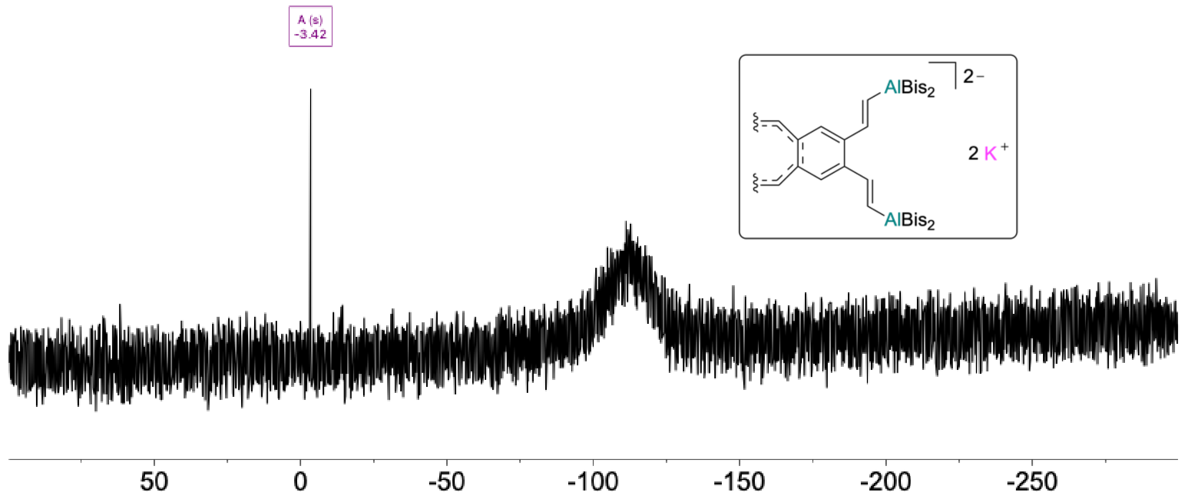

Figure S29a. $^{29}Si\{^{1}H\}$ NMR spectrum of **2A** in C_6D_6 at 298 K, 99 MHz.

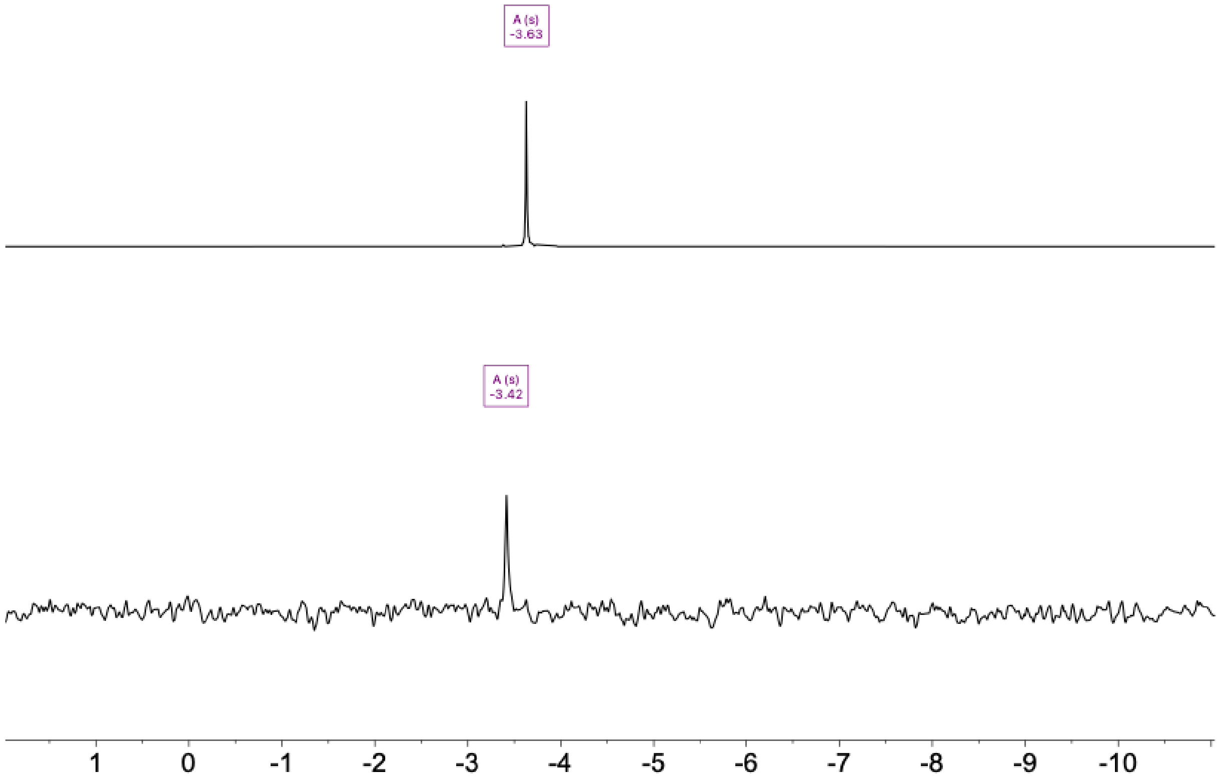

Figure S29b. $^{29}Si\{^{1}H\}$ NMR spectra of **2** (above) and **2A** (below) in C_6D_6 at 298 K, 99 MHz.

Compound **2A**^{cro}

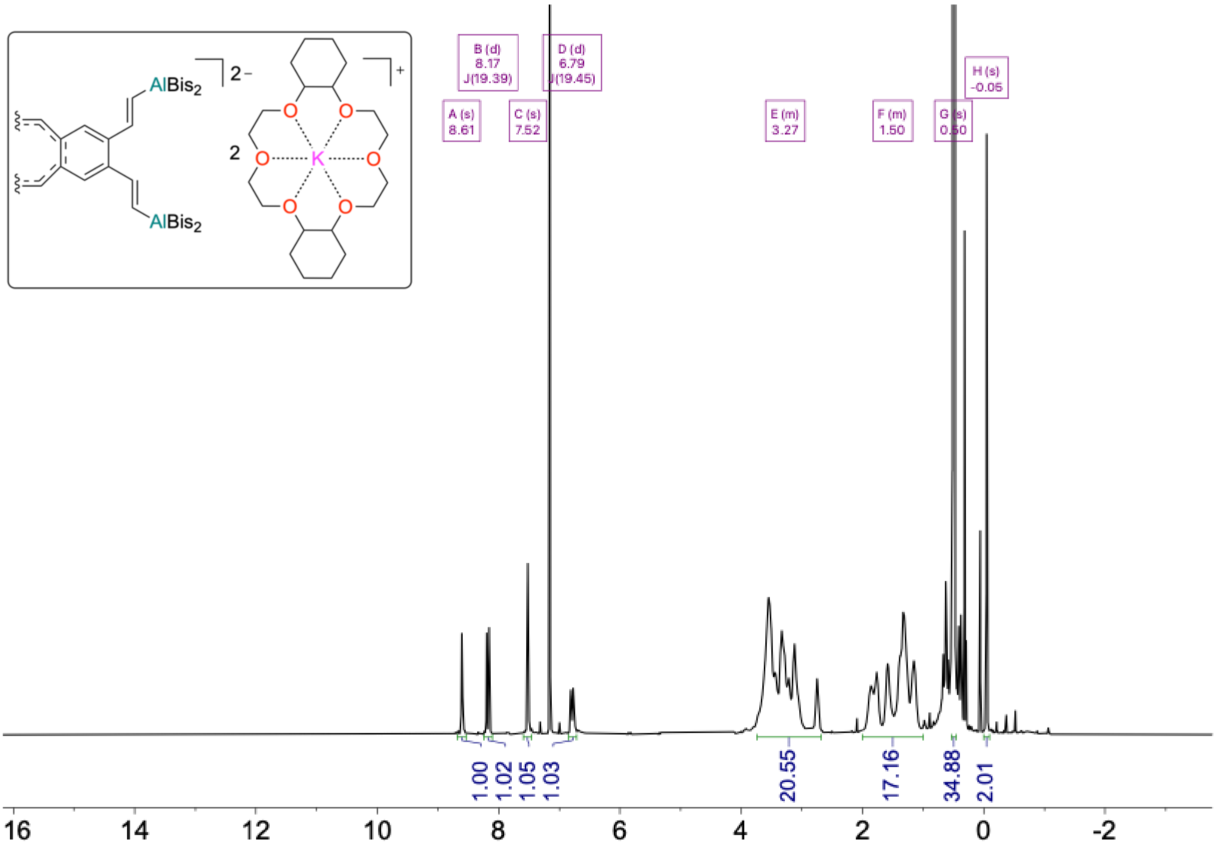

Figure S30. ^{1}H NMR spectrum of **2A**^{cro} in C_6D_6 at 298 K, 500 MHz.

Compound **2A**^{cry}

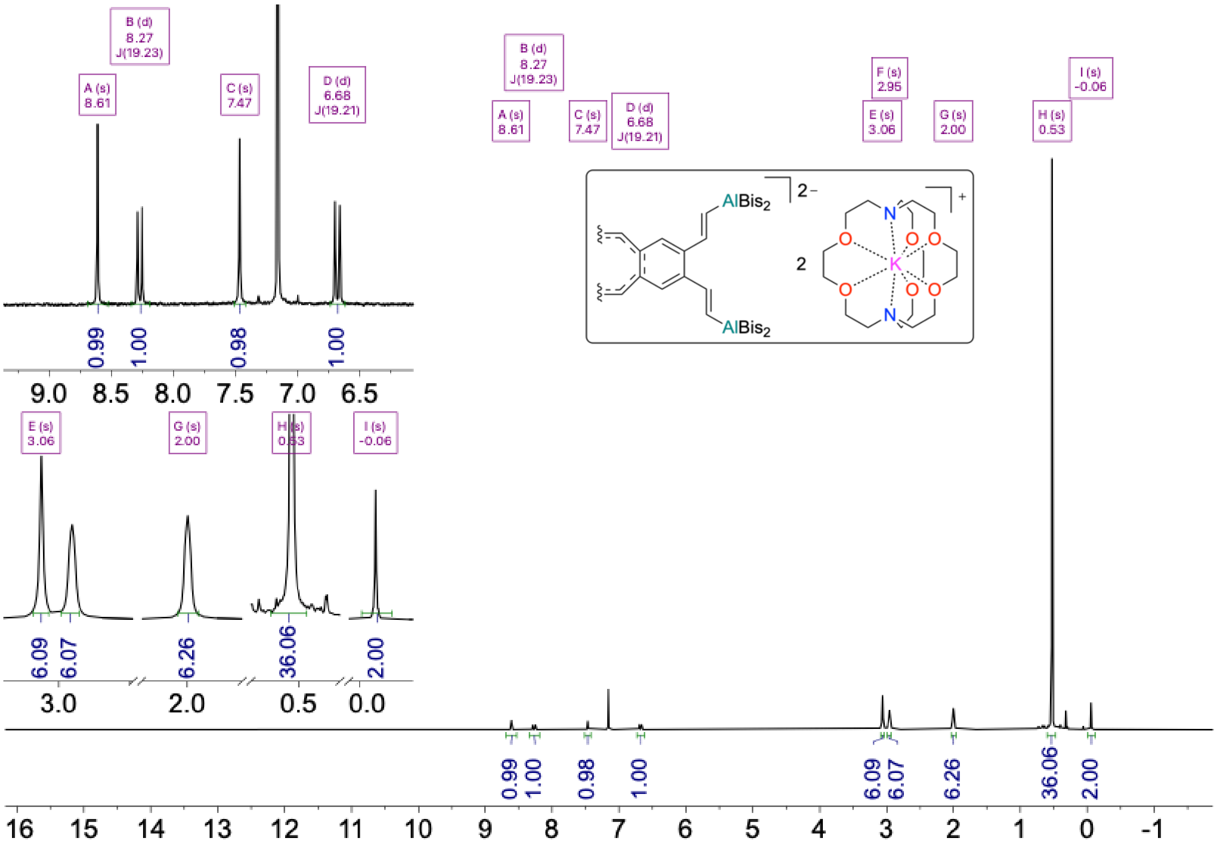

Figure S31. ^{1}H NMR spectrum of **2A**^{cry} in C_6D_6 at 298 K, 500 MHz.

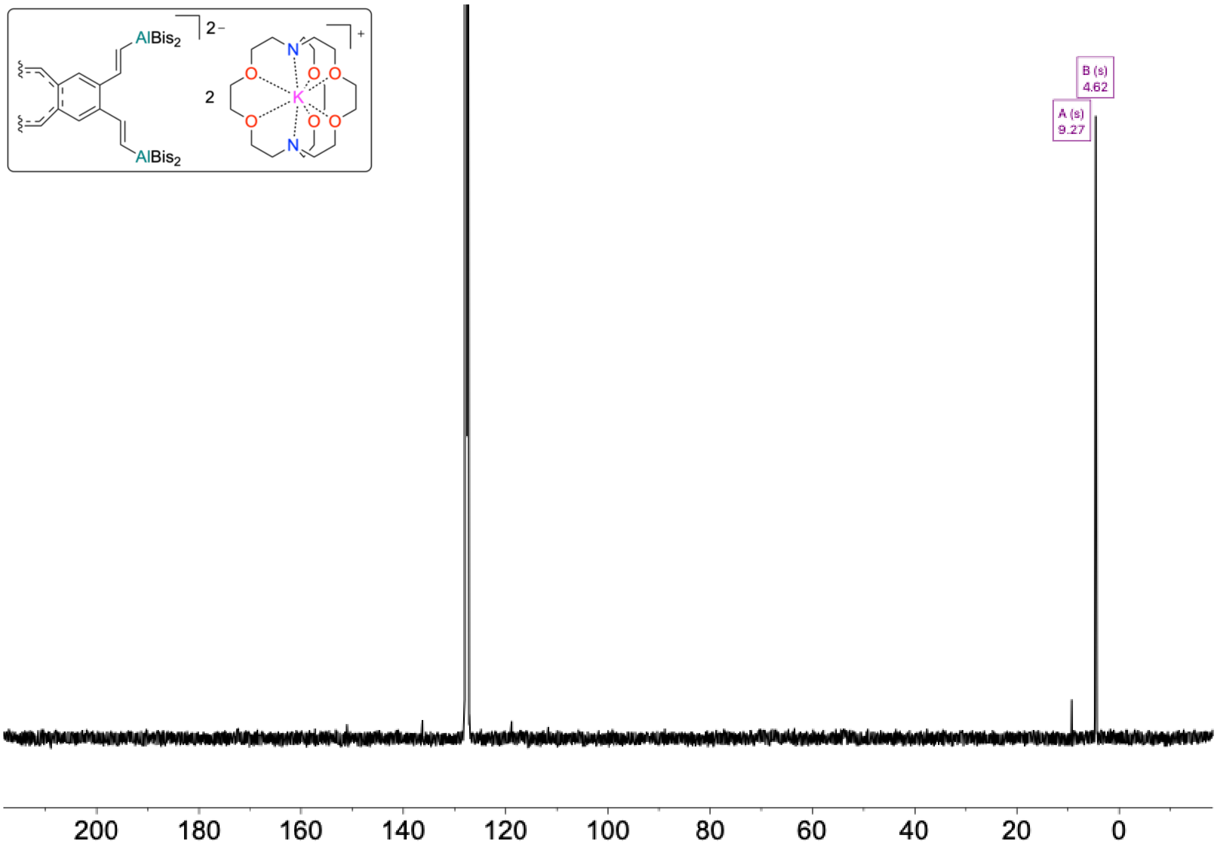

Figure S32. $^{13}C\{^{1}H\}$ NMR spectrum of **2A**^{cry} in C_6D_6 at 298 K, 125 MHz.

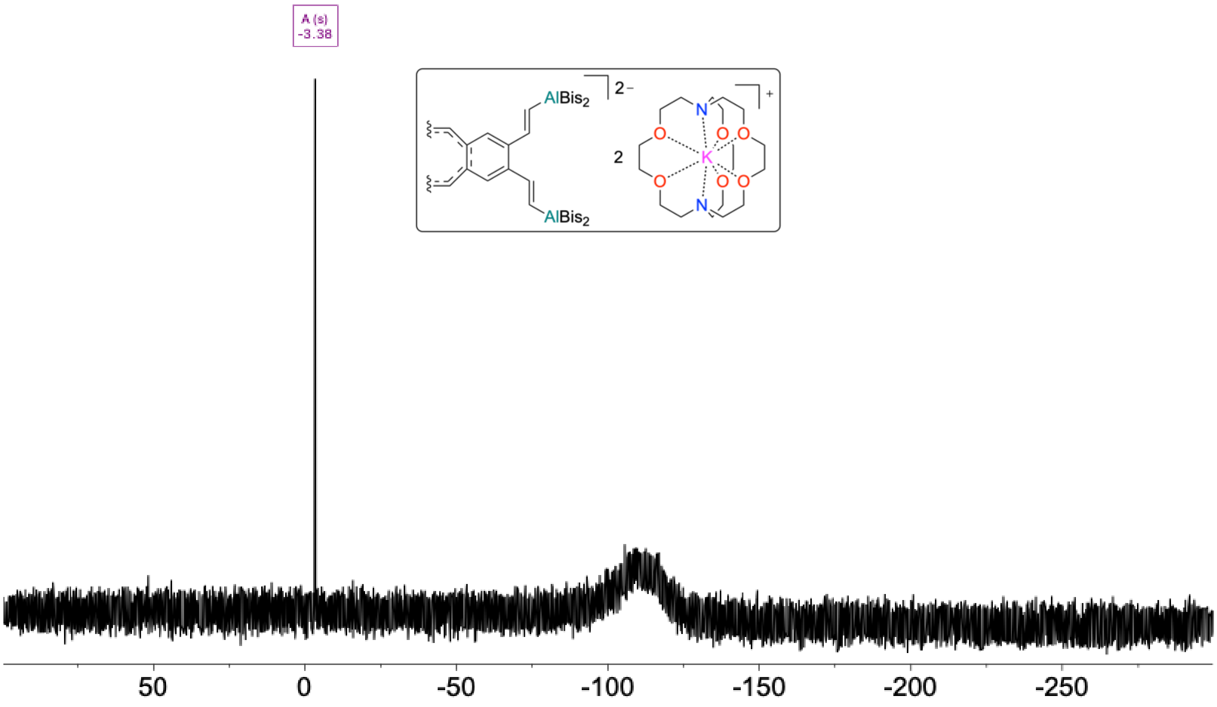

Figure S33. $^{29}Si\{^{1}H\}$ NMR spectrum of **2A**^{cry} in C_6D_6 at 298 K, 99 MHz.

Compound **3A**^{THF}

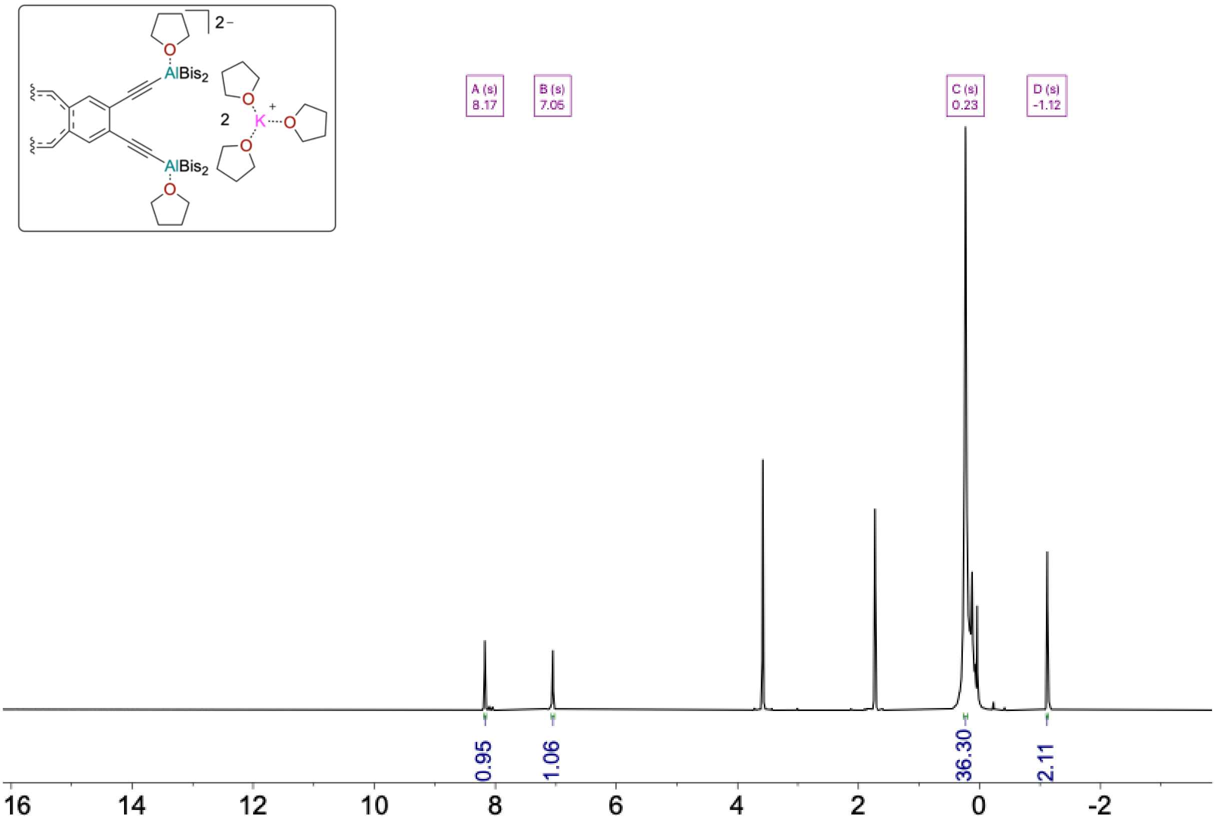

Figure S34. ^{1}H NMR spectrum of **3A**^{THF} in THF-d_8 at 298 K, 500 MHz.

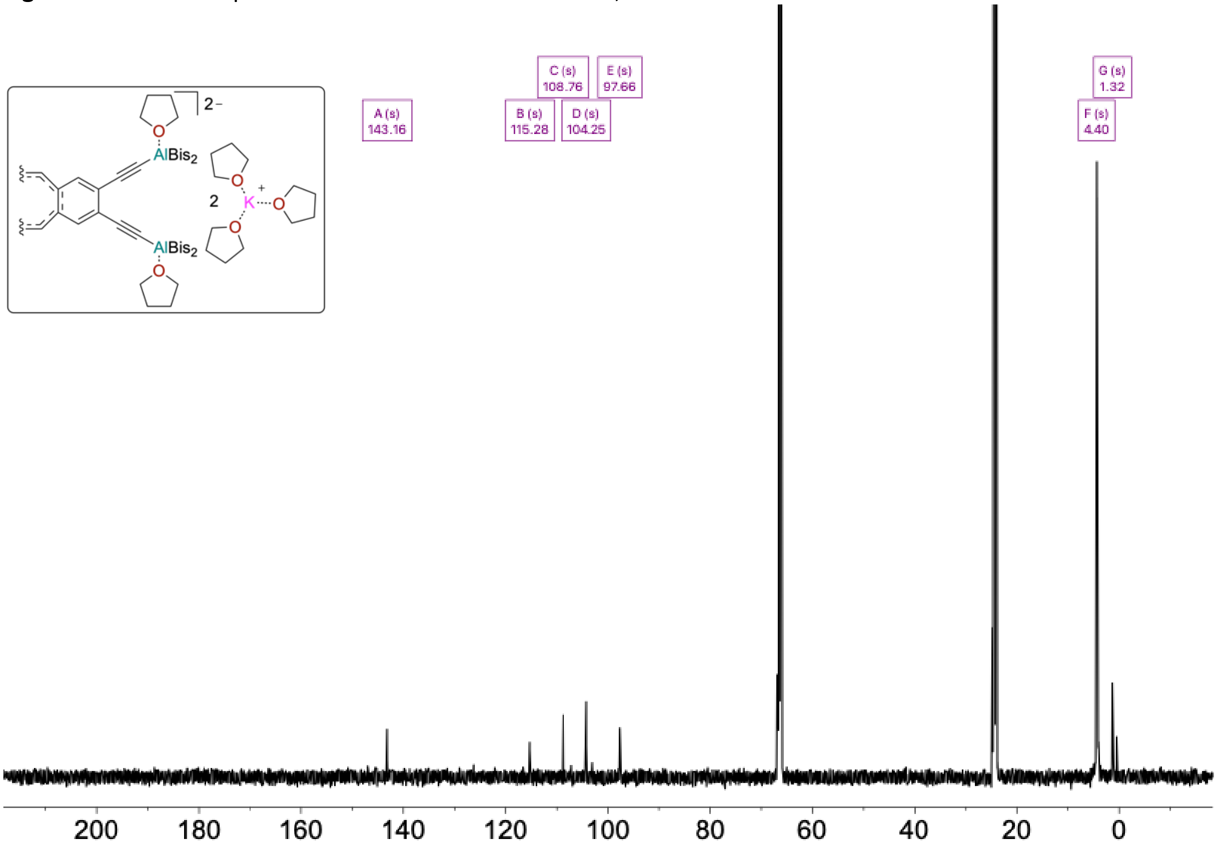

Figure S35. $^{13}C\{^{1}H\}$ NMR spectrum of **3A**^{THF} in THF-d_8 at 298 K, 125 MHz.

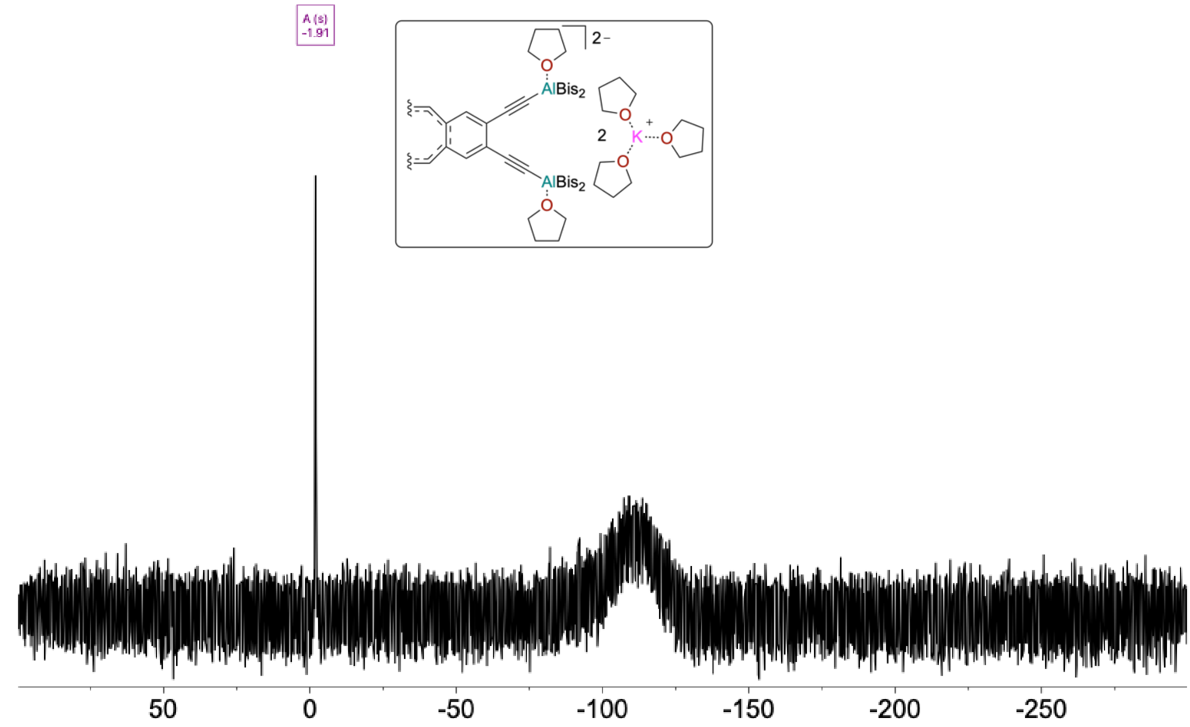

Figure S36. $^{29}Si\{^{1}H\}$ NMR spectrum of **3A**^{THF} in THF-d_8 at 298 K, 99 MHz.

Compound **3A**

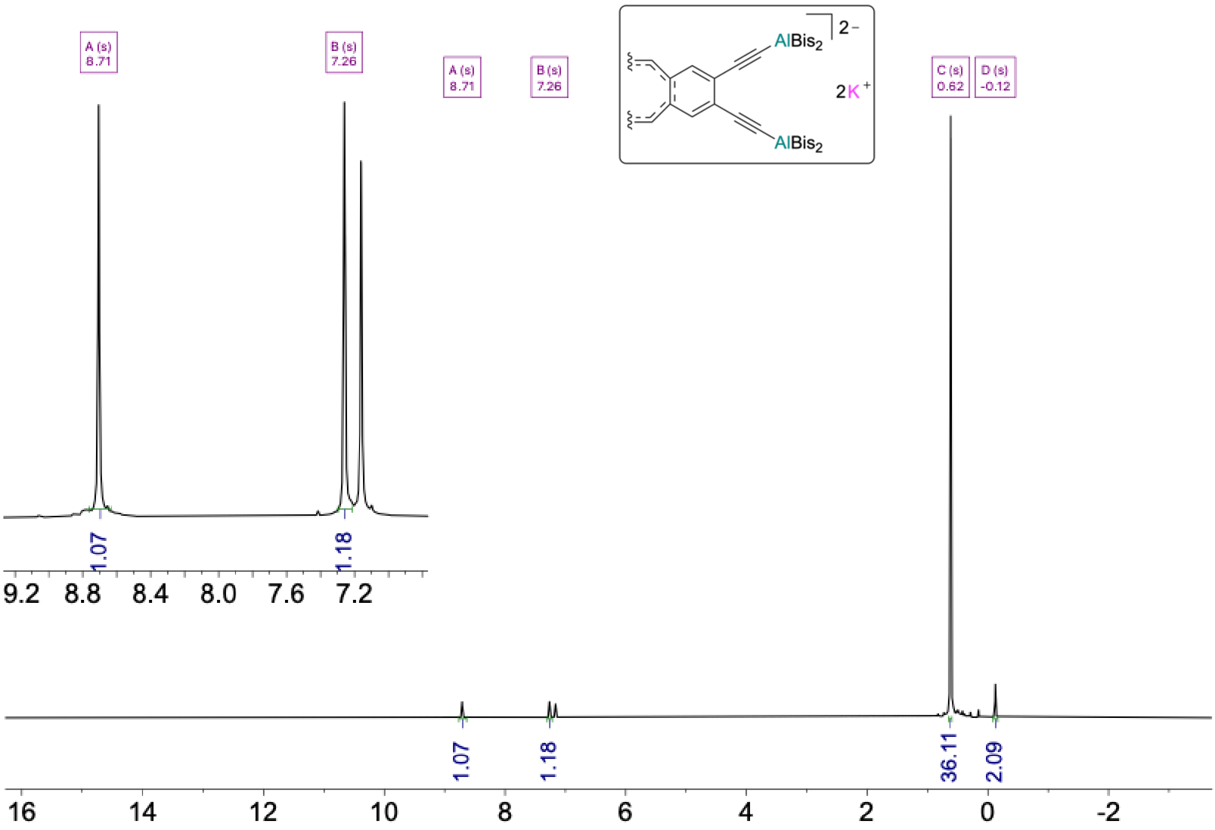

Figure S37. ^{1}H NMR spectrum of **3A** in C_6D_6 at 298 K, 500 MHz.

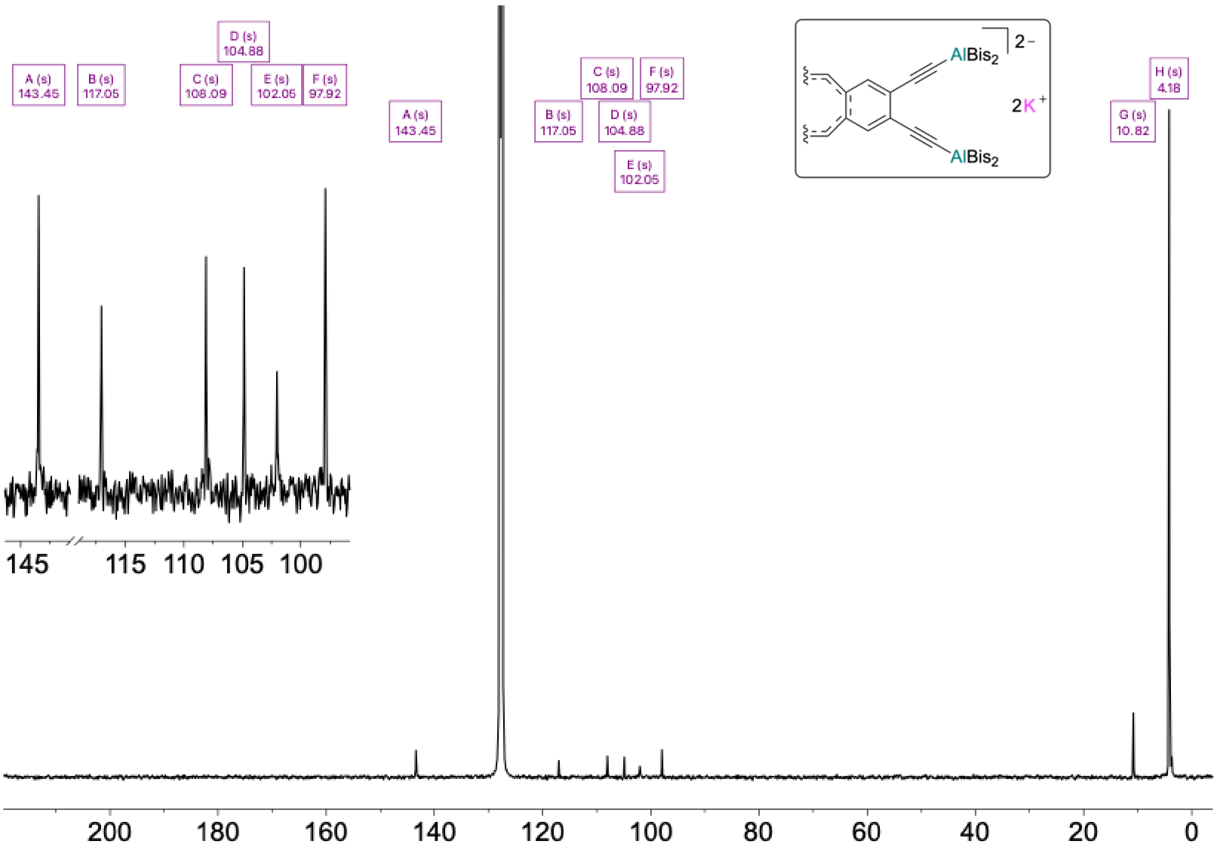

Figure S38. $^{13}C\{^1H\}$ NMR spectrum of **3A** in C_6D_6 at 298 K, 125 MHz.

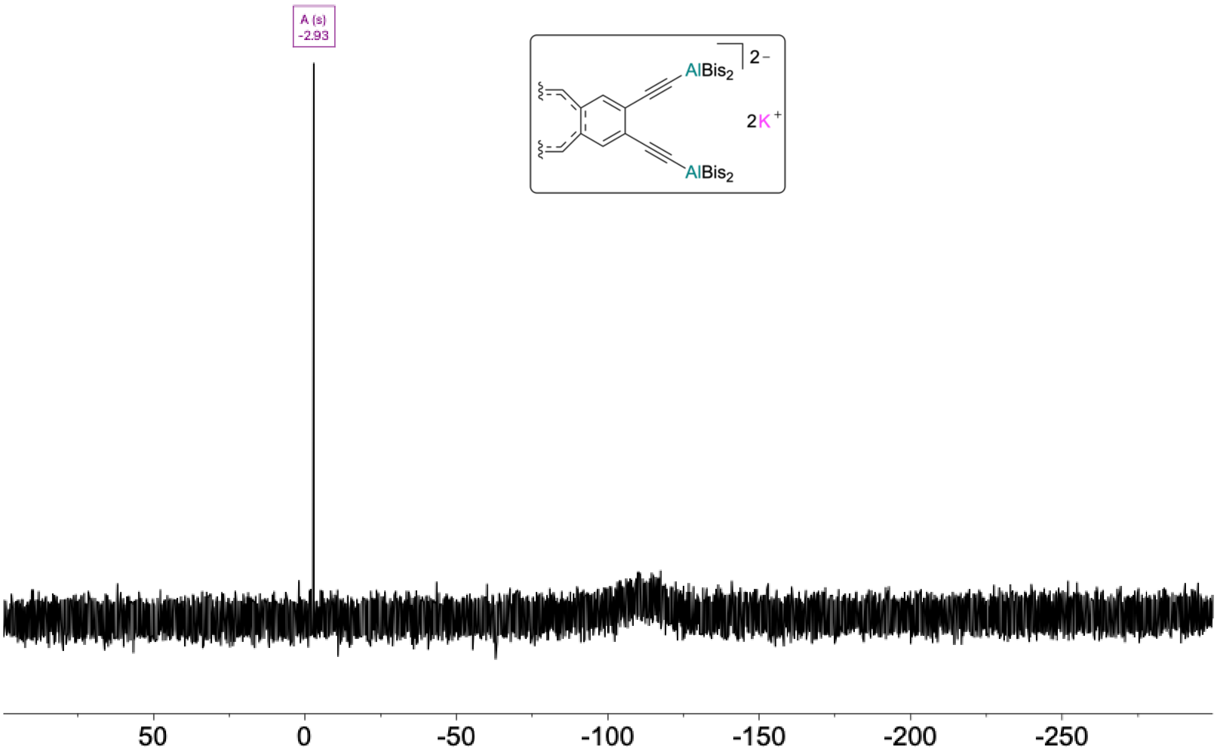

Figure S39a. $^{29}Si\{^1H\}$ NMR spectrum of **3A** in C_6D_6 at 298 K, 99 MHz.

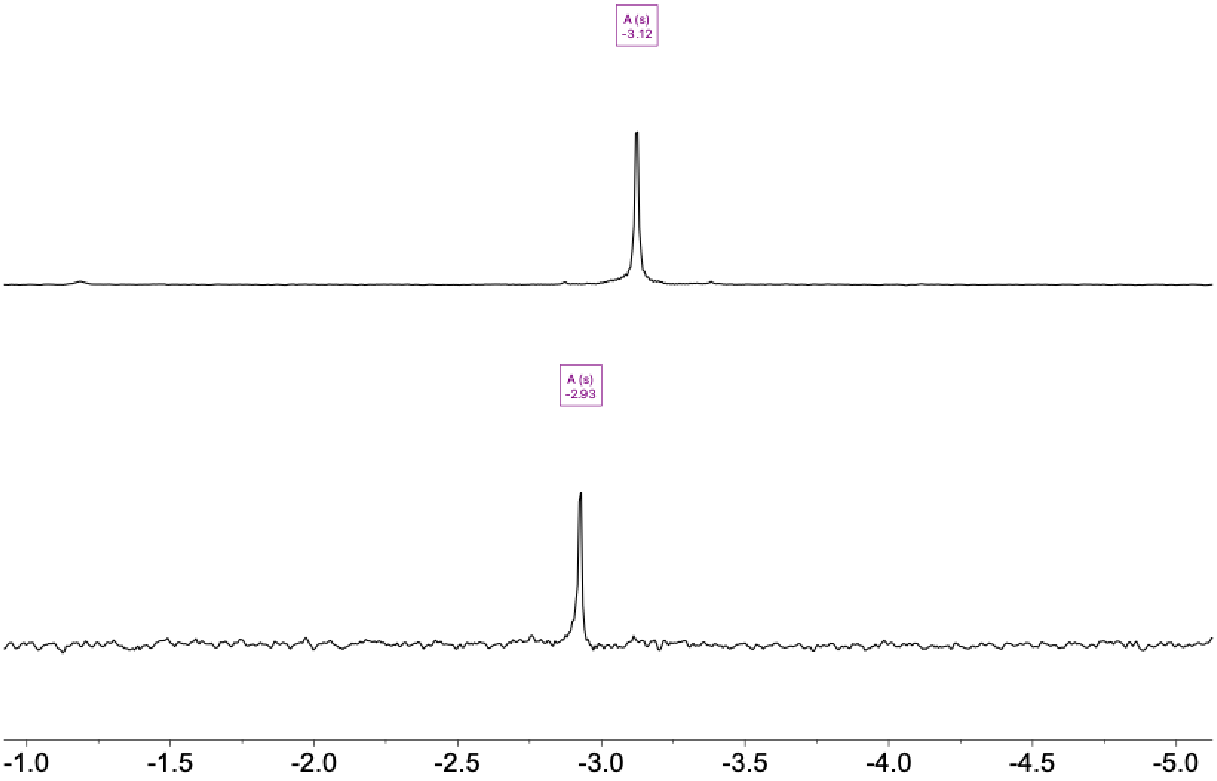

Figure S39b. $^{29}Si\{^{1}H\}$ NMR spectra of **3** (above) and **3A** (below) in C_6D_6 at 298 K, 99 MHz.

Compound **3A**^{cro}

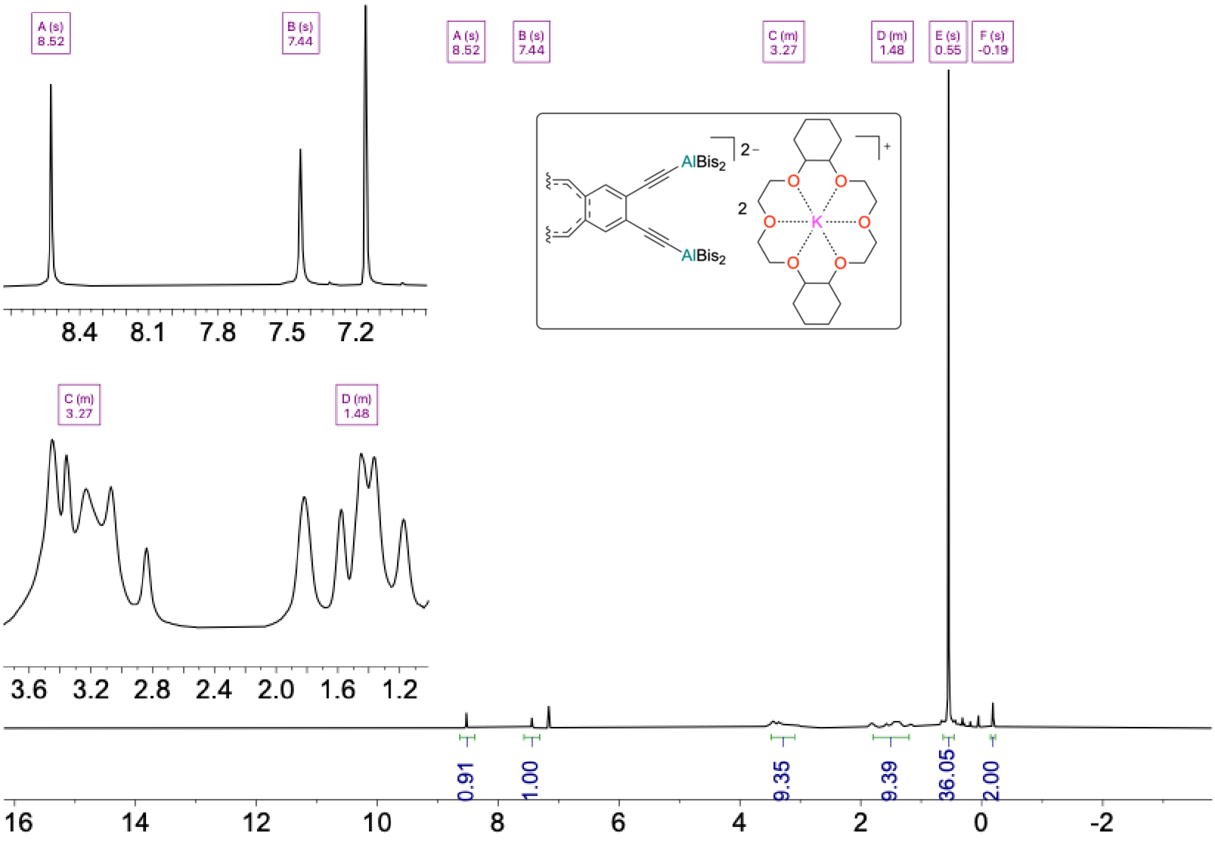

Figure S40. ^{1}H NMR spectrum of **3A**^{cro} in C_6D_6 at 298 K, 500 MHz.

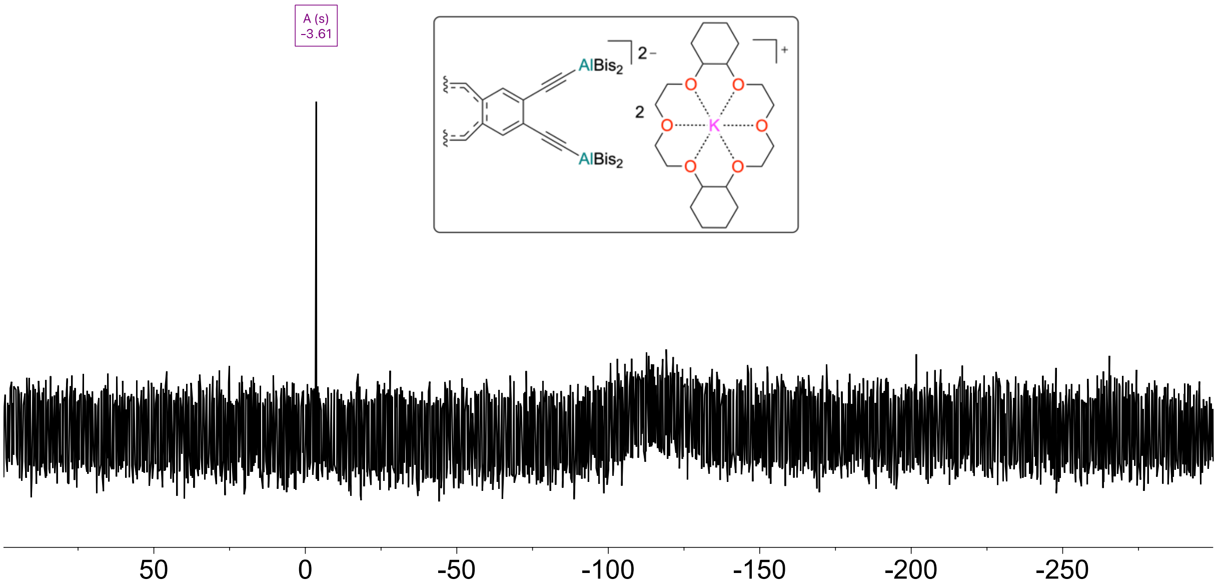

Figure S41. $^{29}Si\{^1H\}$ NMR spectrum of **3A**^{cro} in C_6D_6 at 298 K, 99 MHz.

Compound **3A**^{cry}

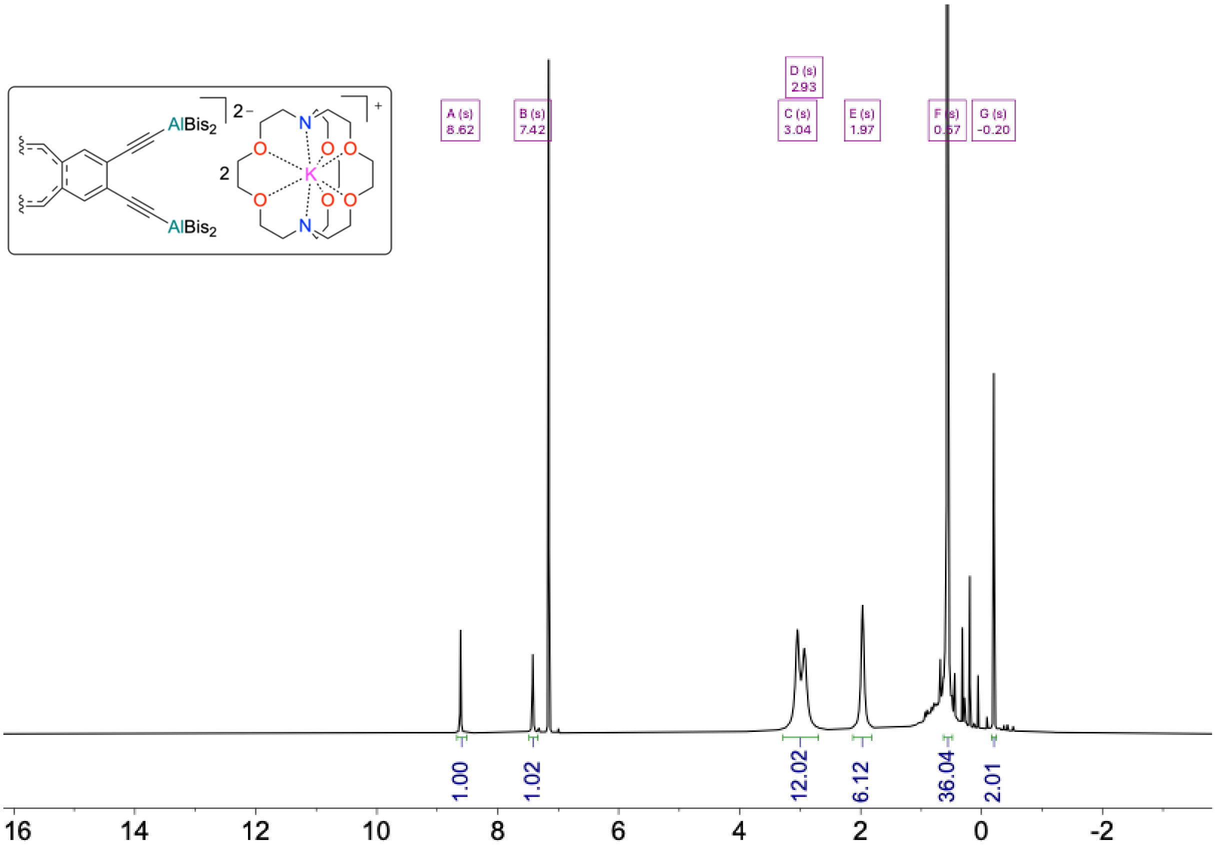

Figure S42. ^1H NMR spectrum of **3A**^{cry} in C_6D_6 at 298 K, 500 MHz.

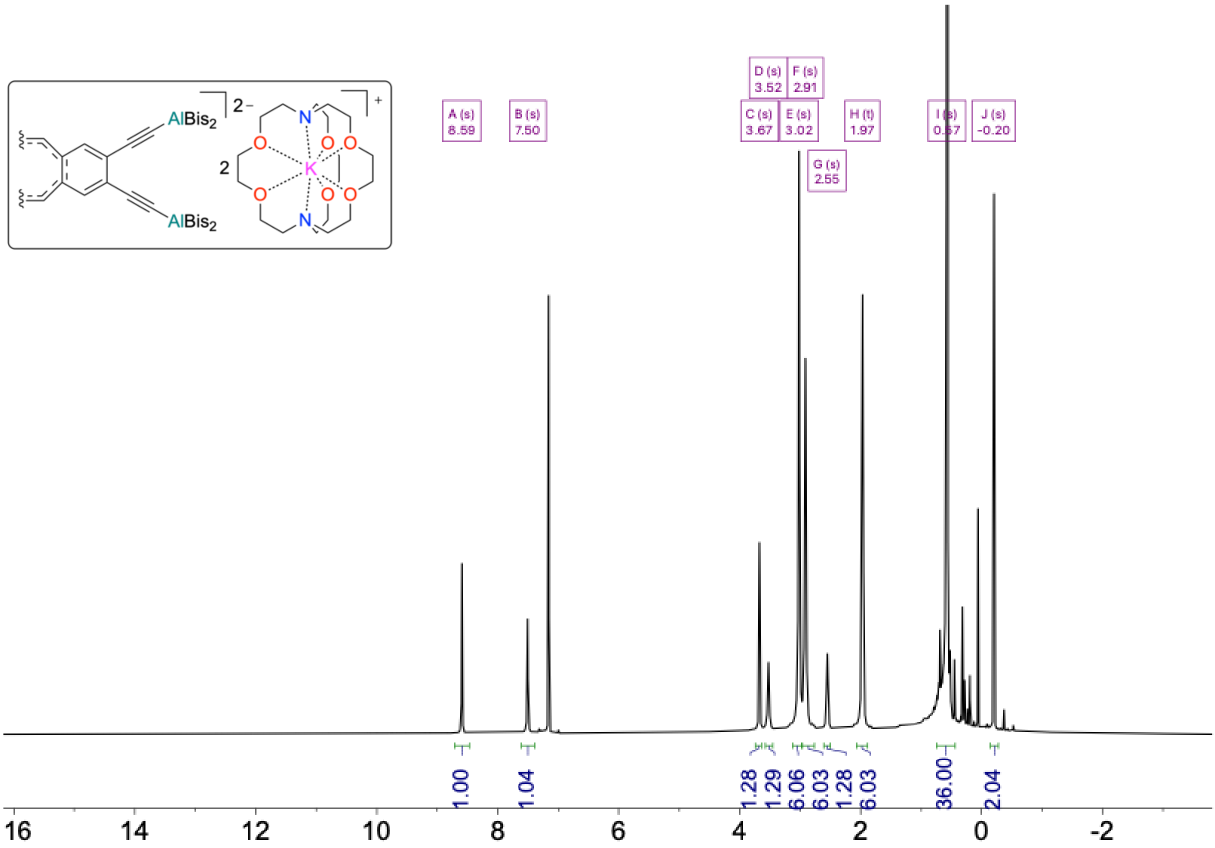

Figure S43. ^{1}H NMR spectrum of **3A**^{cry} with ex. [2.2.2]cryptand in C_6D_6 at 298 K, 500 MHz.

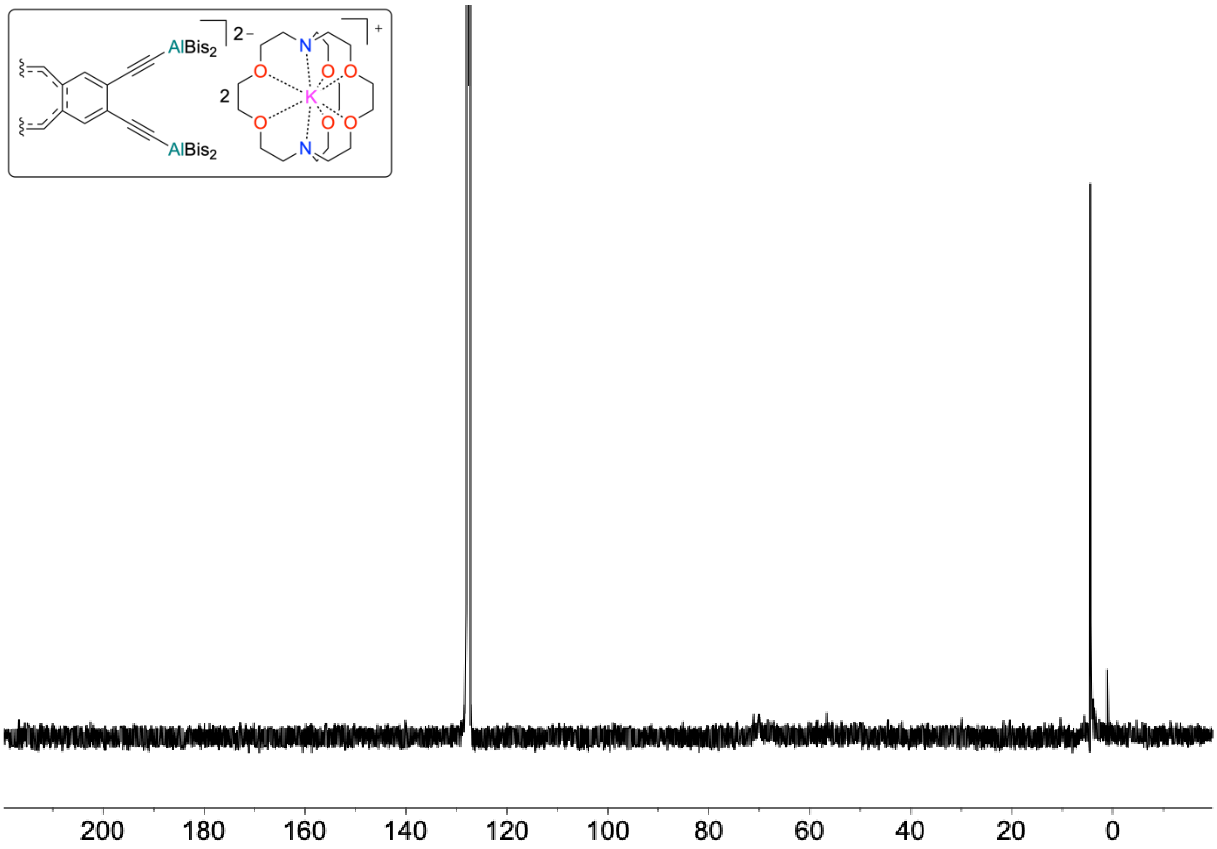

Figure S44. $^{13}C\{^{1}H\}$ NMR spectrum of **3A**^{cry} in C_6D_6 at 298 K, 125 MHz.

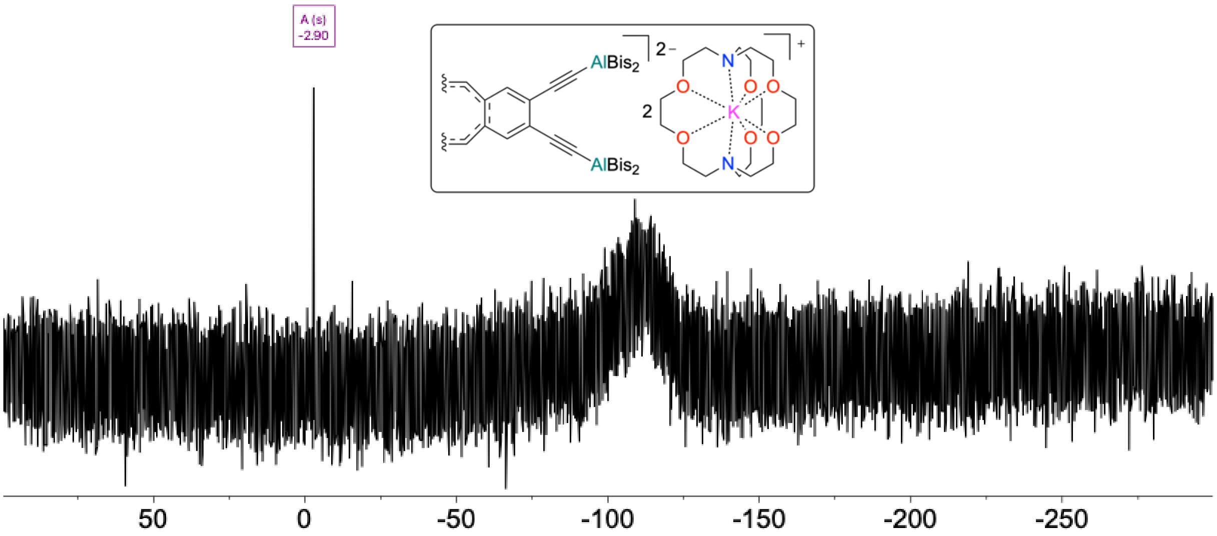

Figure S45. $^{29}Si\{^{1}H\}$ NMR spectrum of **3A**^{cry} in C_6D_6 at 298 K, 99 MHz.

Compound **4A**^{THF}

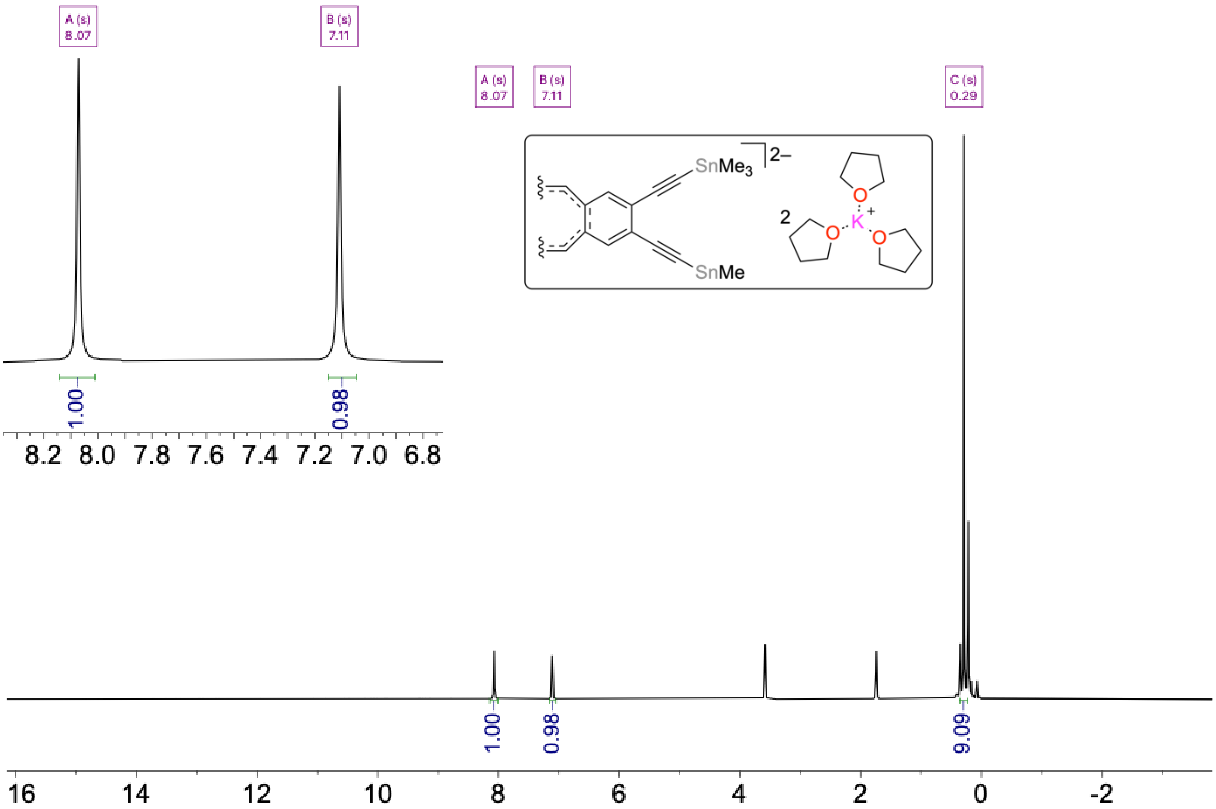

Figure S46. ^{1}H NMR spectrum of **4A**^{THF} in THF-d_8 at 298 K, 500 MHz.

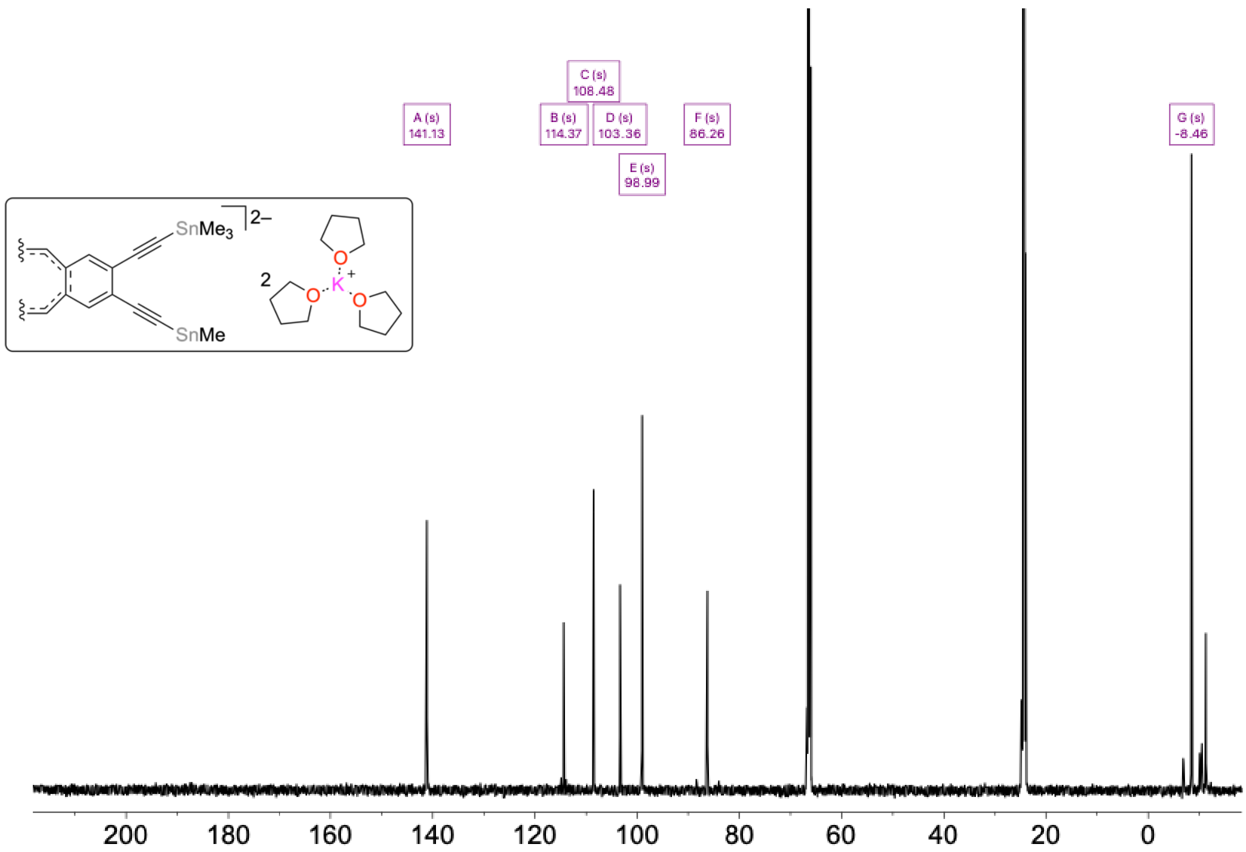

Figure S47. $^{13}C\{^{1}H\}$ NMR spectrum of **4A**^{THF} in THF-d_8 at 298 K, 125 MHz.

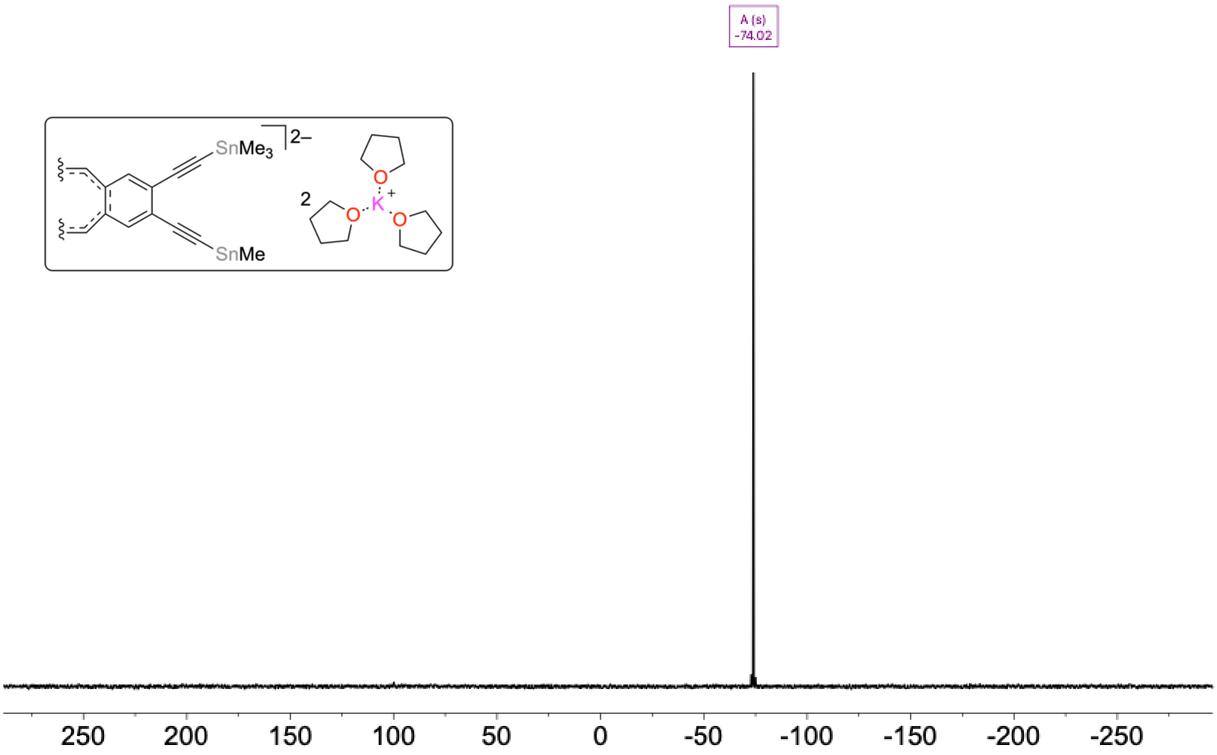

Figure S48a. $^{119}Sn\{^{1}H\}$ NMR spectrum of **4A**^{THF} in THF-d_8 at 298 K, 186 MHz.

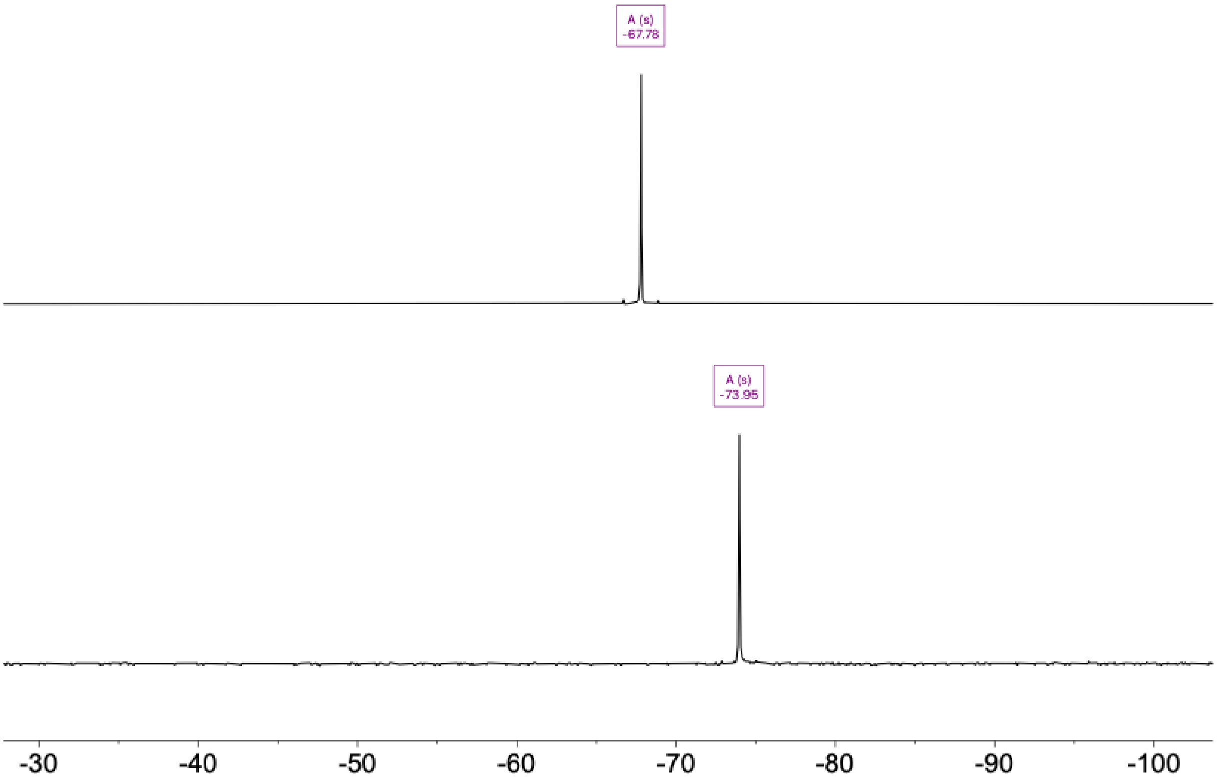

Figure S48b. $^{119}Sn\{^1H\}$ NMR spectra of **4** (above) and **4A**^{THF} (below) in THF-d_8 at 298 K, 186 MHz.

UV-Vis Spectra

The UV-Vis samples were prepared in a glove box and filled into a cuvette fitted with a PTFE stopper, which was additionally sealed with PTFE tape. The samples were removed from the glove box shortly before each measurement. When the UV-Vis spectra were recorded, rapid oxidation of the compounds was observed, accompanied by a decrease in colour intensity. This is illustrated by the examples in Figure S49. Due to the rapid oxidation, the concentration could not be validly determined and therefore no extinction coefficient can be given. To verify that the observed adsorption maxima were due to the reduced species, a second measurement was carried out on the samples after exposure to ambient air for 1 min and mixing.

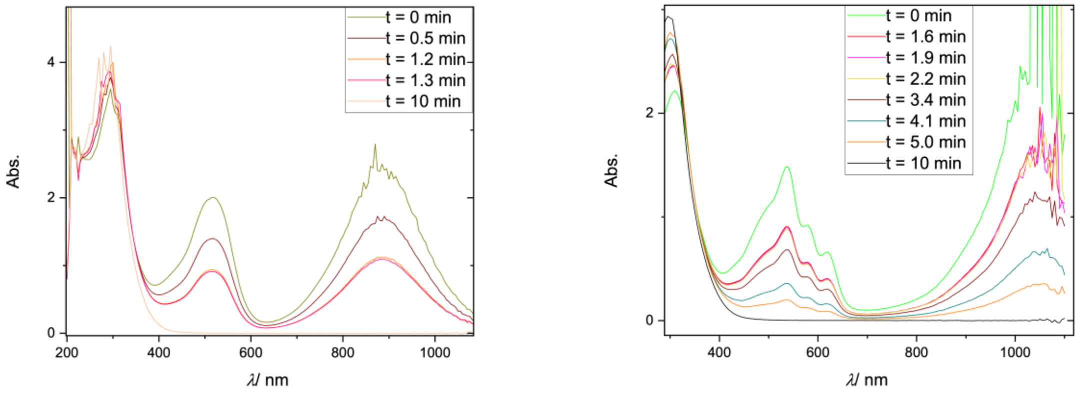

Figure S49. UV-Vis spectra stack of compound **1A**^{THF} (left) and **1A**^{cry} (right) in THF at 298 K at different times after removal from the glove box to illustrate the rapid oxidation of the compounds.

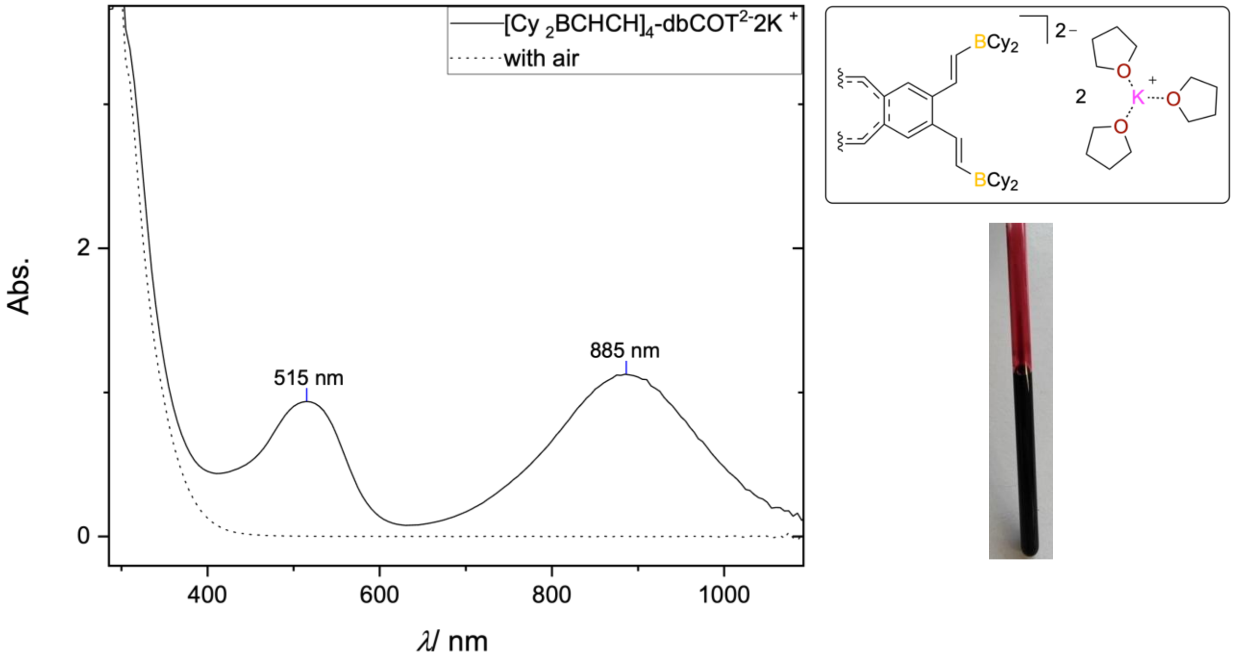

Figure S50. UV-Vis spectrum of compound **1A**^{THF} in THF at 298 K.

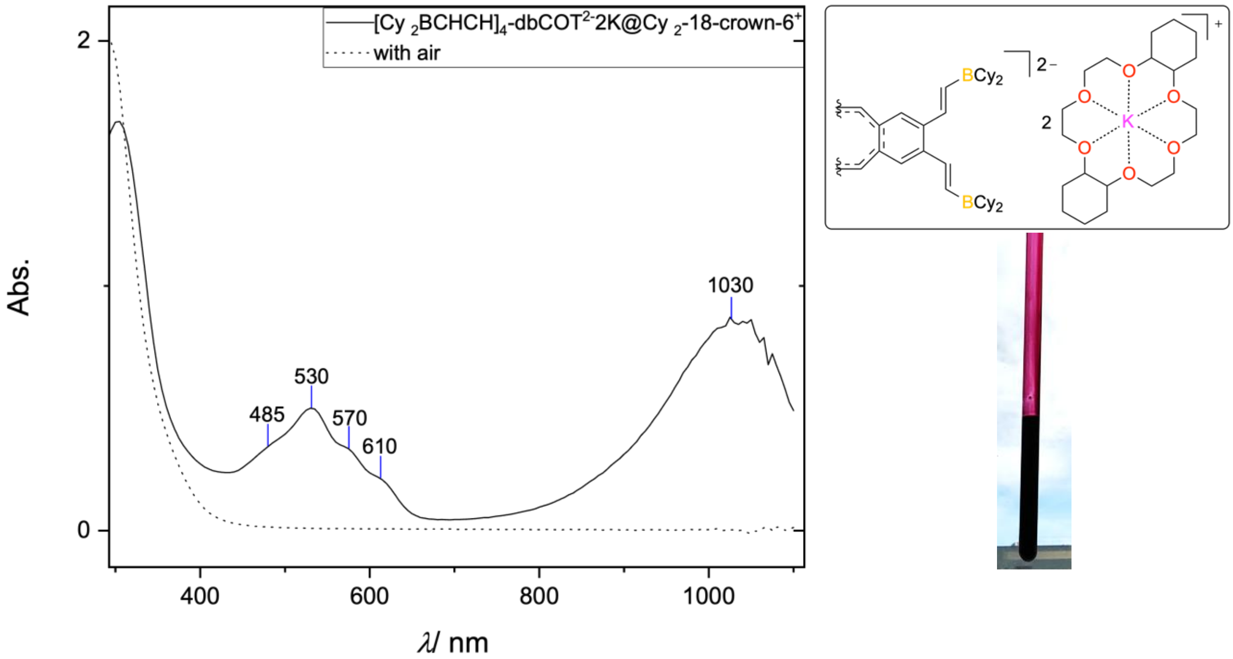

Figure S51. UV-Vis spectrum of compound **1A**^{cro} in THF at 298 K.

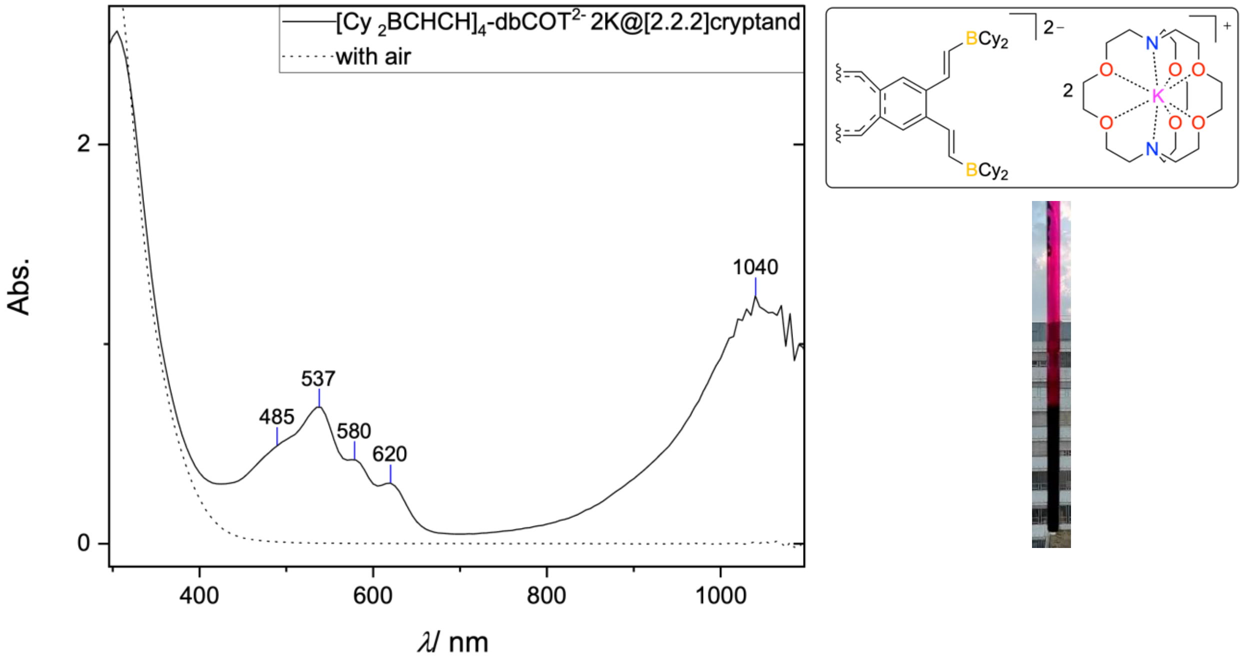

Figure S52. UV-Vis spectrum of compound **1A**^{cry} in THF at 298 K.

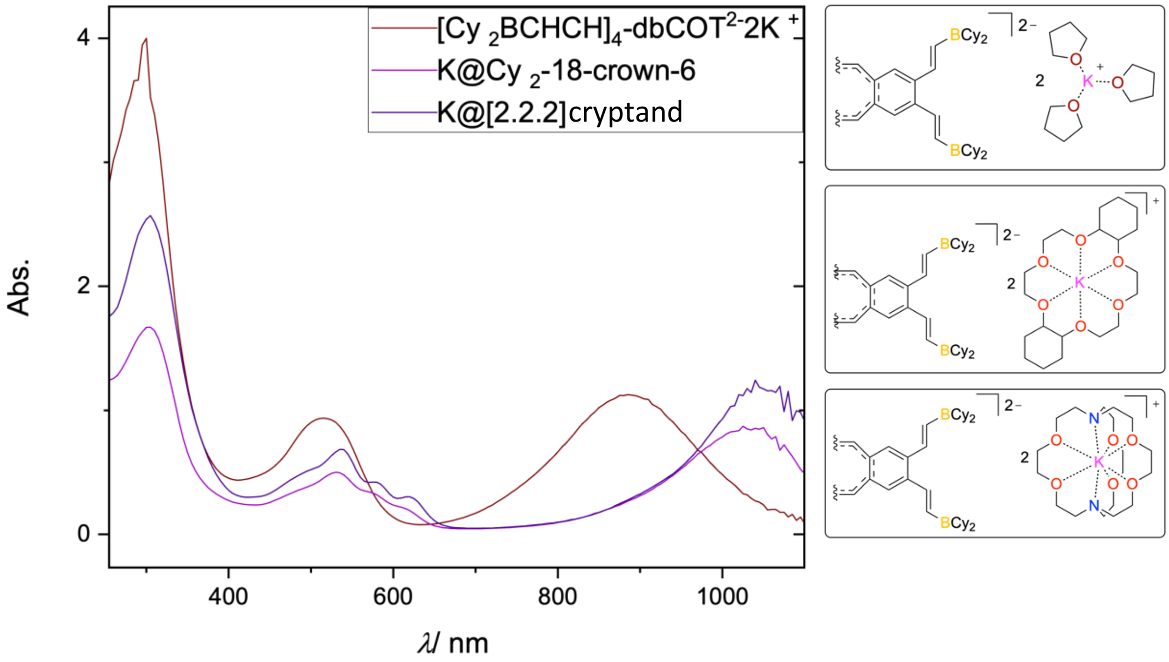

Figure S53. UV-Vis spectra stack of compound **1A**^{THF}, **1A**^{cro}, **1A**^{cry} in THF at 298 K.

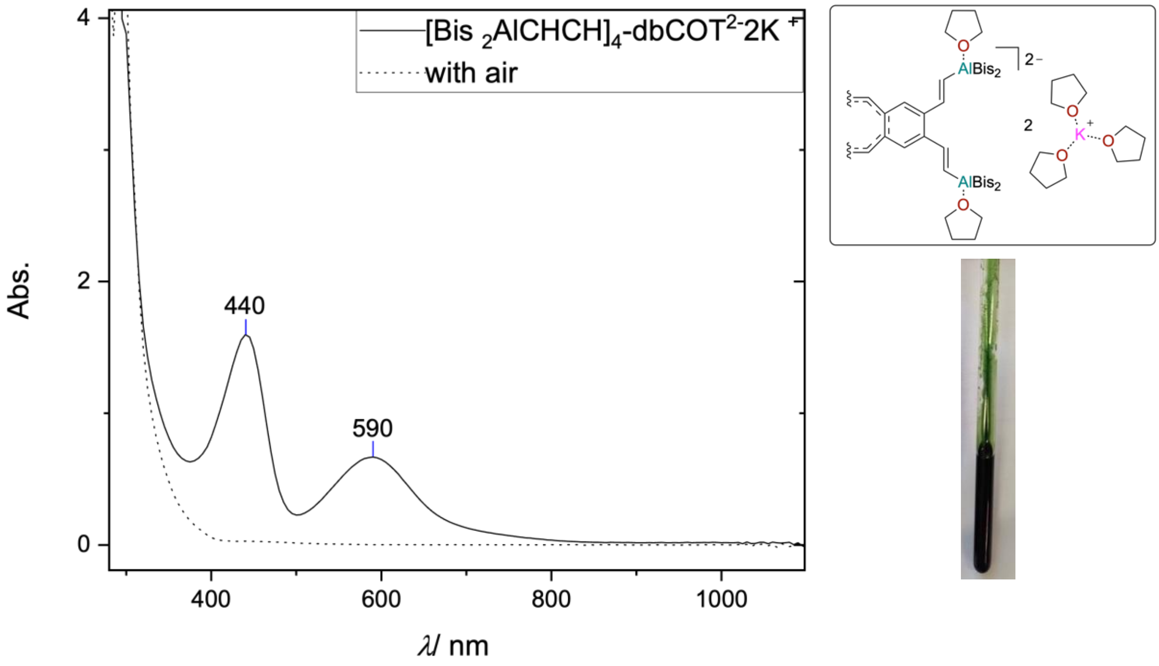

Figure S54. UV-Vis spectrum of compound **2A**^{THF} in THF at 298 K.

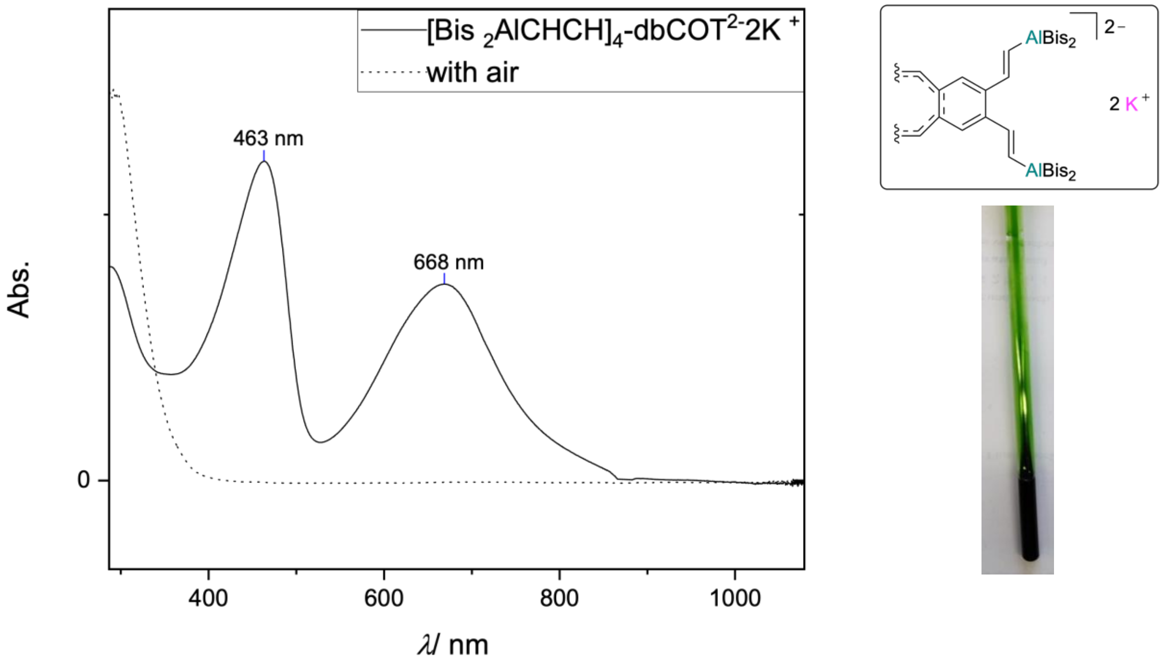

Figure S55. UV-Vis spectrum of compound **2A** in C_6H_6 at 298 K.

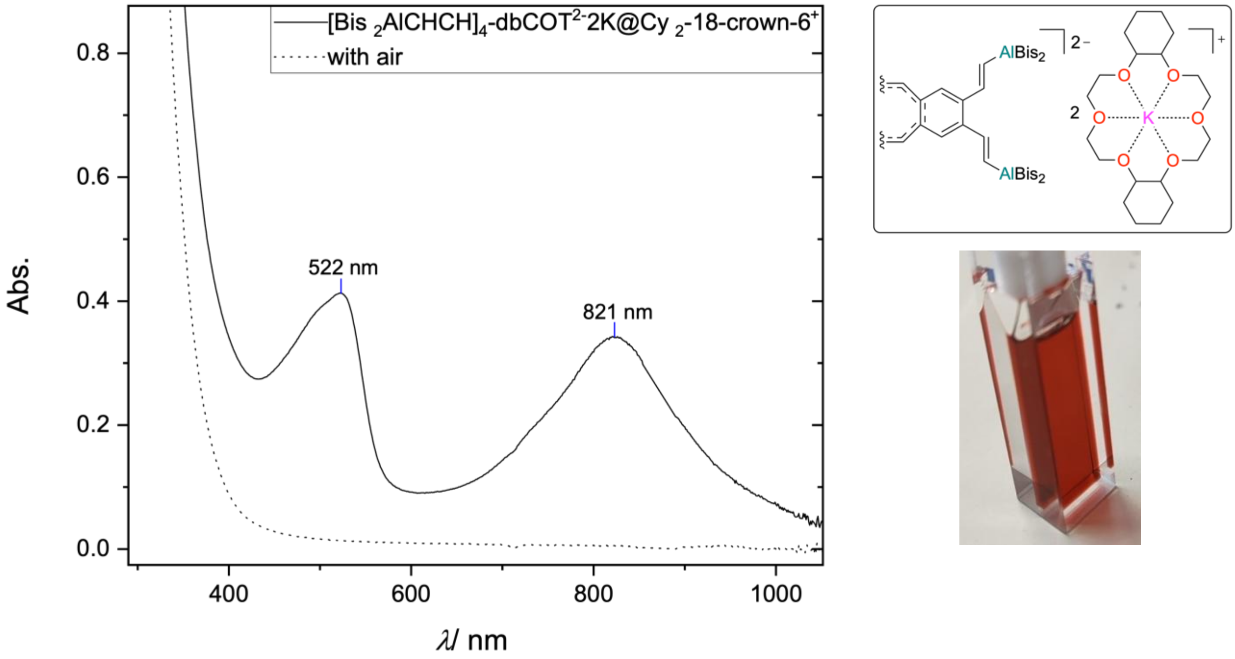

Figure S56. UV-Vis spectrum of compound **2A**^{cro} in C_6H_6 at 298 K.

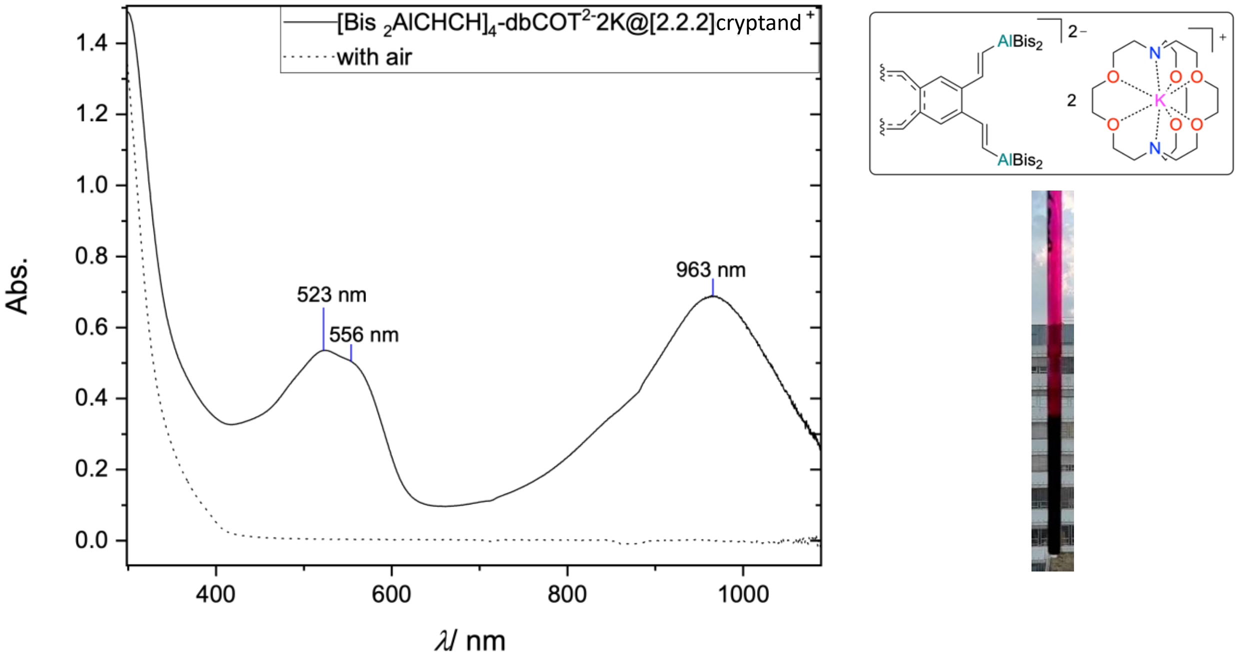

Figure S57. UV-Vis spectrum of compound **2A**^{cry} in C_6H_6 at 298 K.

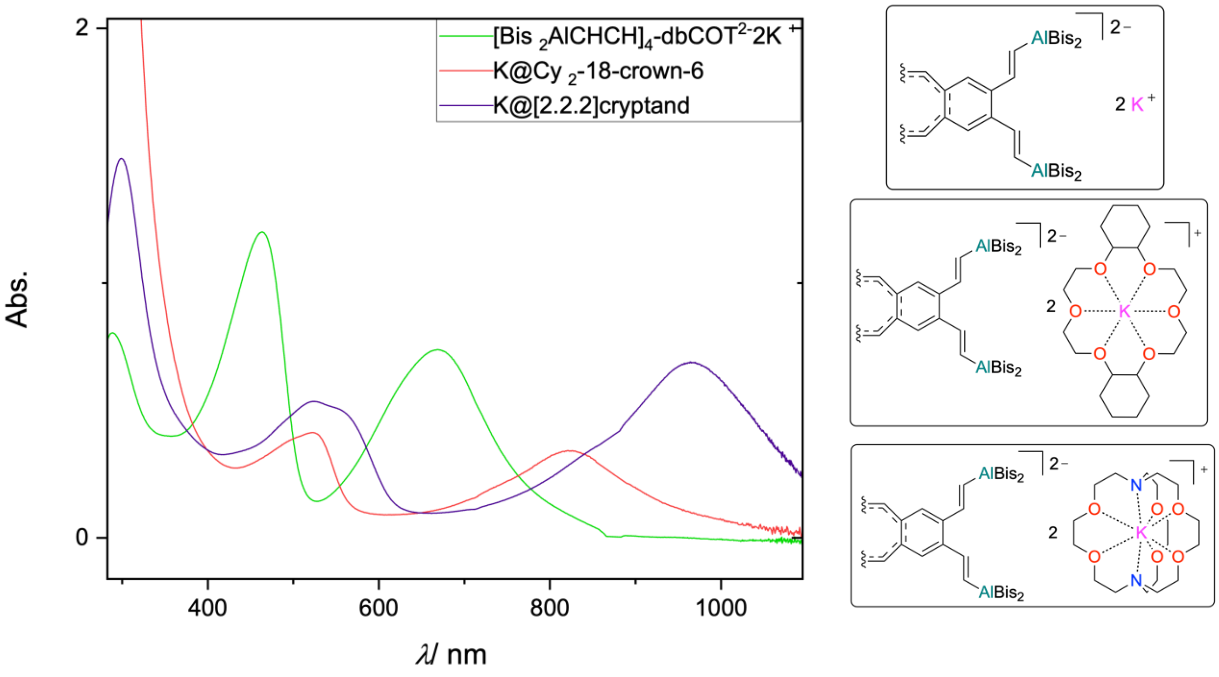

Figure S58. UV-Vis spectra stack of compound **2A**, **2A**^{cro}, **2A**^{cry} in C_6H_6 at 298 K.

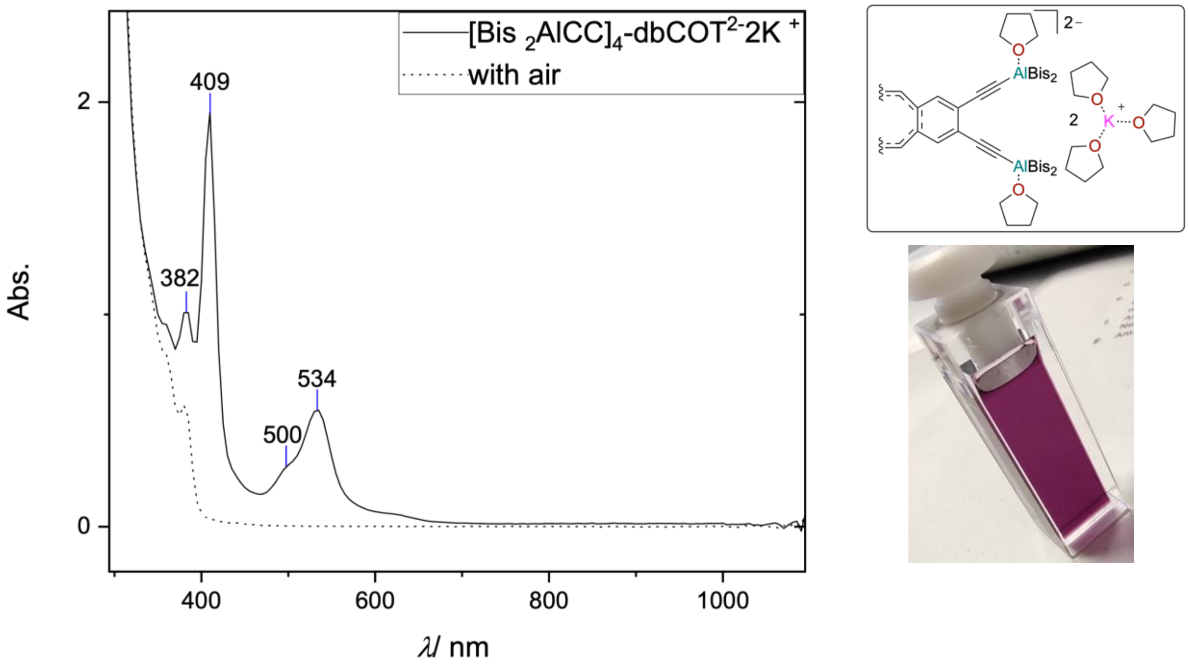

Figure S59. UV-Vis spectrum of compound **3A**^{THF} in THF at 298 K.

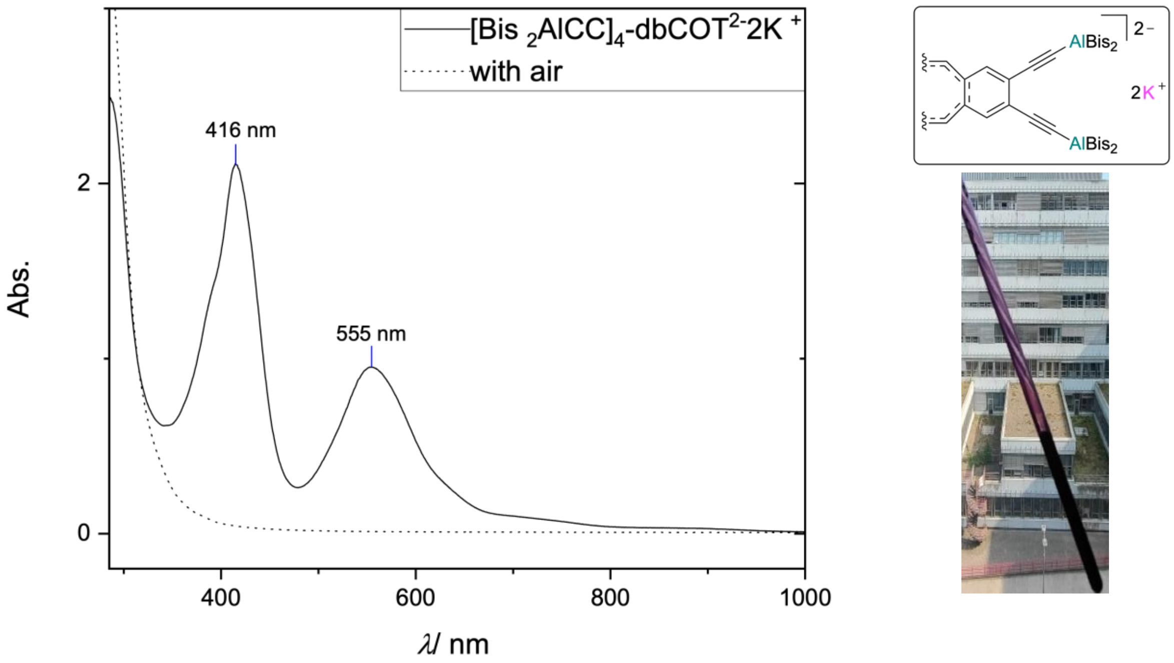

Figure S60. UV-Vis spectrum of compound **3A** in C_6H_6 at 298 K.

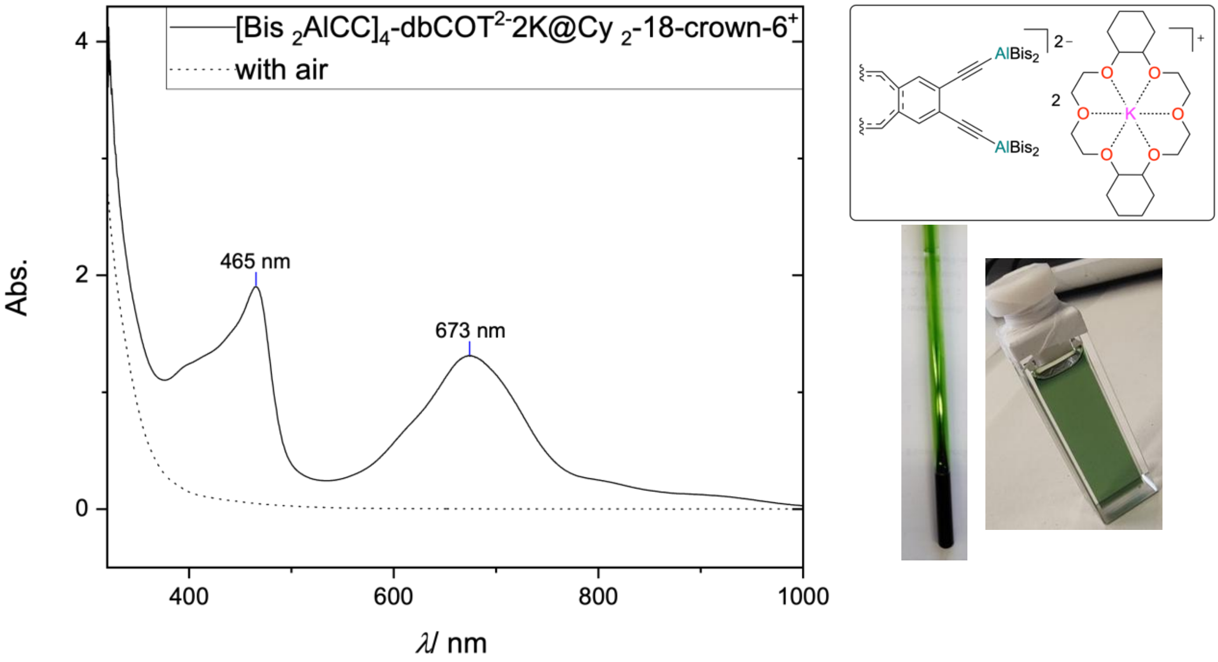

Figure S61. UV-Vis spectrum of compound **3A**^{cro} in C_6H_6 at 298 K.

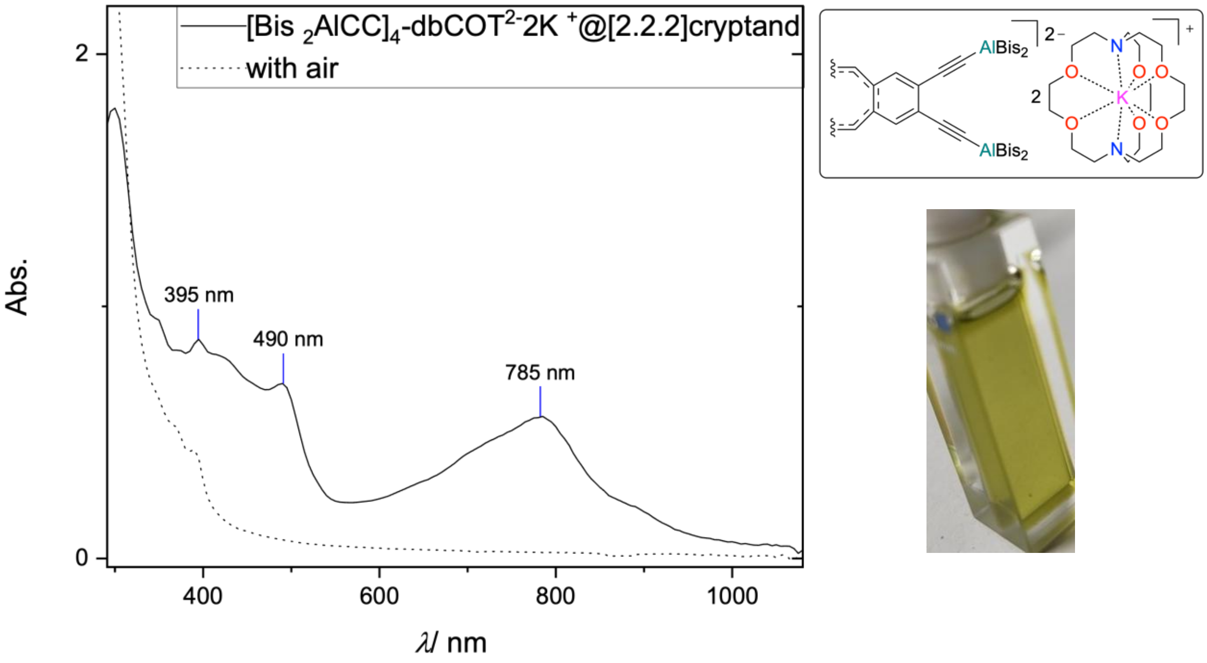

Figure S62. UV-Vis spectrum of compound **3A**^{cry} in C_6H_6 at 298 K.

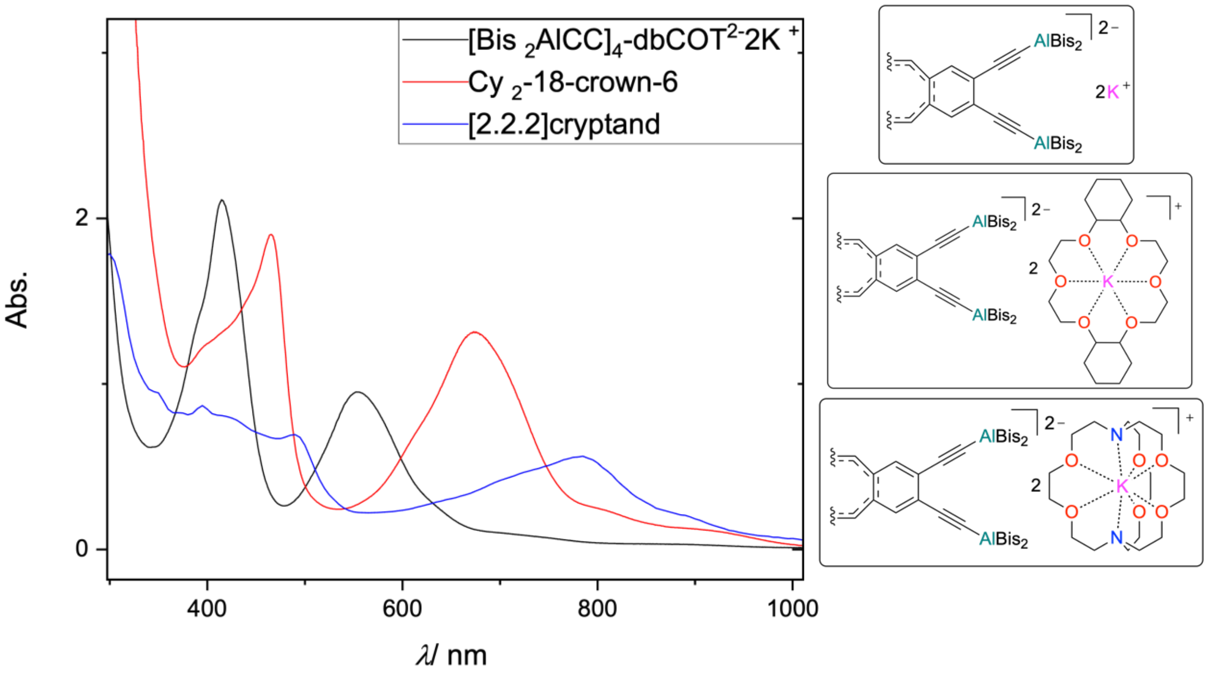

Figure S63. UV-Vis spectra stack of compound **3A**, **3A**^{cro}, **3A**^{cry} in C_6H_6 at 298 K.

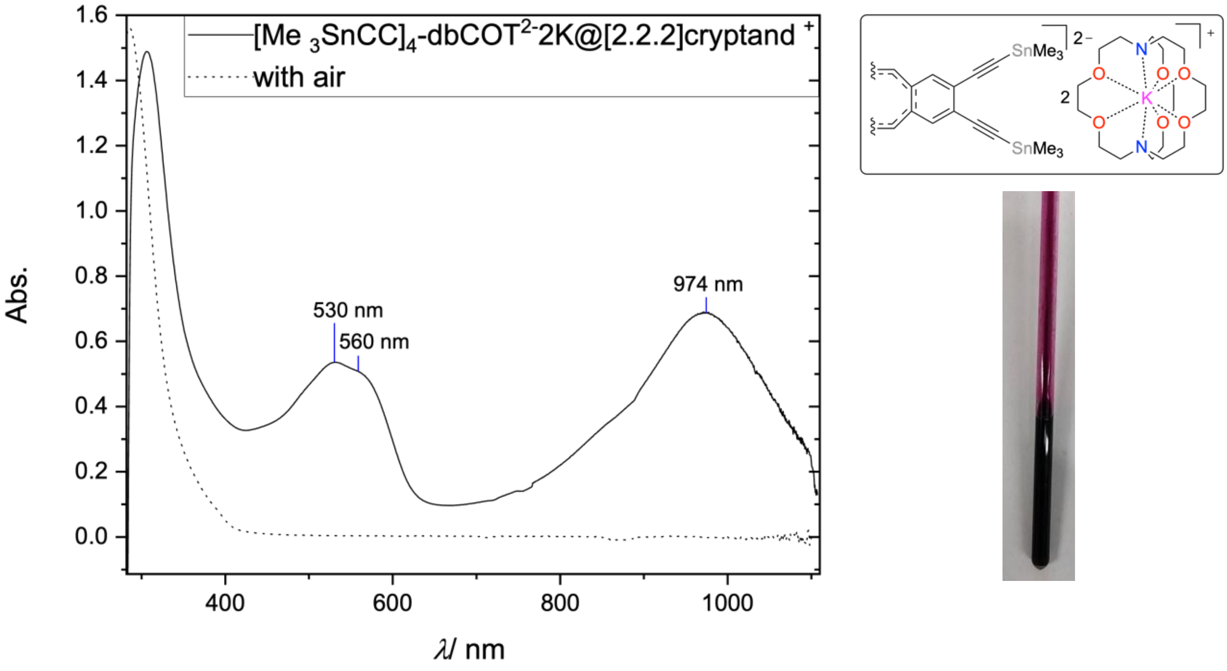

Figure S64. UV-Vis spectrum of compound **4A**^{cry} in THF at 298 K.

X-ray Crystallography

Suitable crystals were obtained from saturated solutions as mentioned in the experimental section for the corresponding compounds. The crystals were selected, coated with paratoneN oil, mounted on a glass fibre and transferred onto the goniometer of diffractometer. Using Olex2,[2] the structures were solved with the ShelXT[3] structure solution program using Intrinsic Phasing and refined with the ShelXL[4] refinement package using Least Squares minimization or refined with the olex2.refine[5] refinement package using Gauss-Newton minimization. All experimental data are listed in Tables S4–S5.

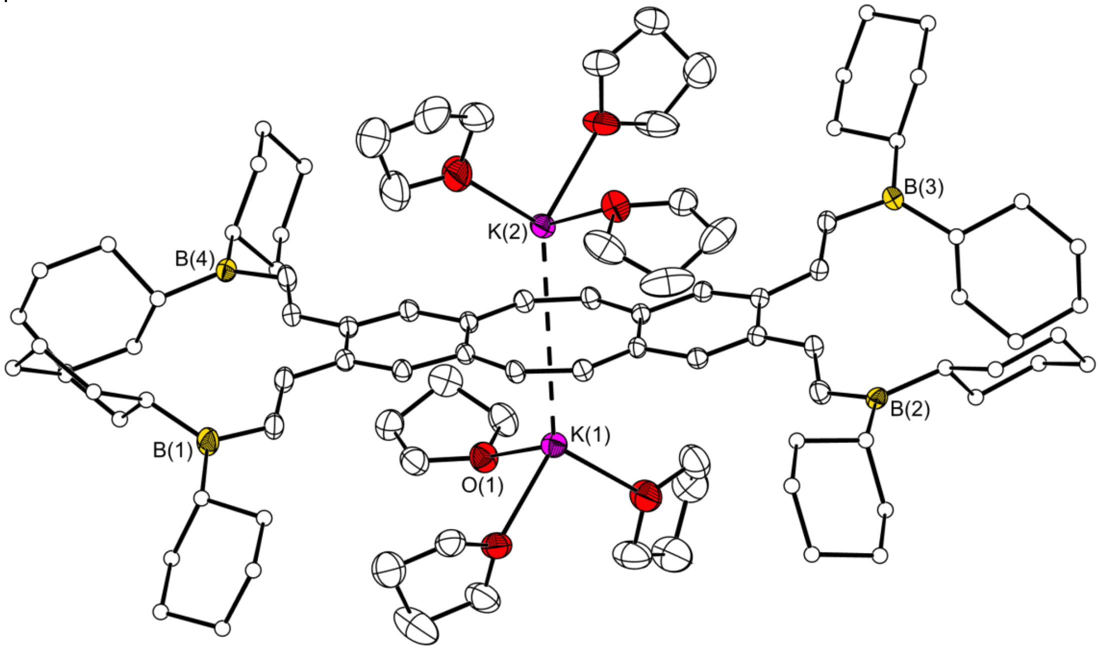

Figure S65. Molecular structure of **1A**^{THF} in the crystalline state. Displacement ellipsoids drawn at the 40% probability level. H atoms are omitted and the carbon atoms of the cyclohexyl groups are only shown as spheres for clarity.

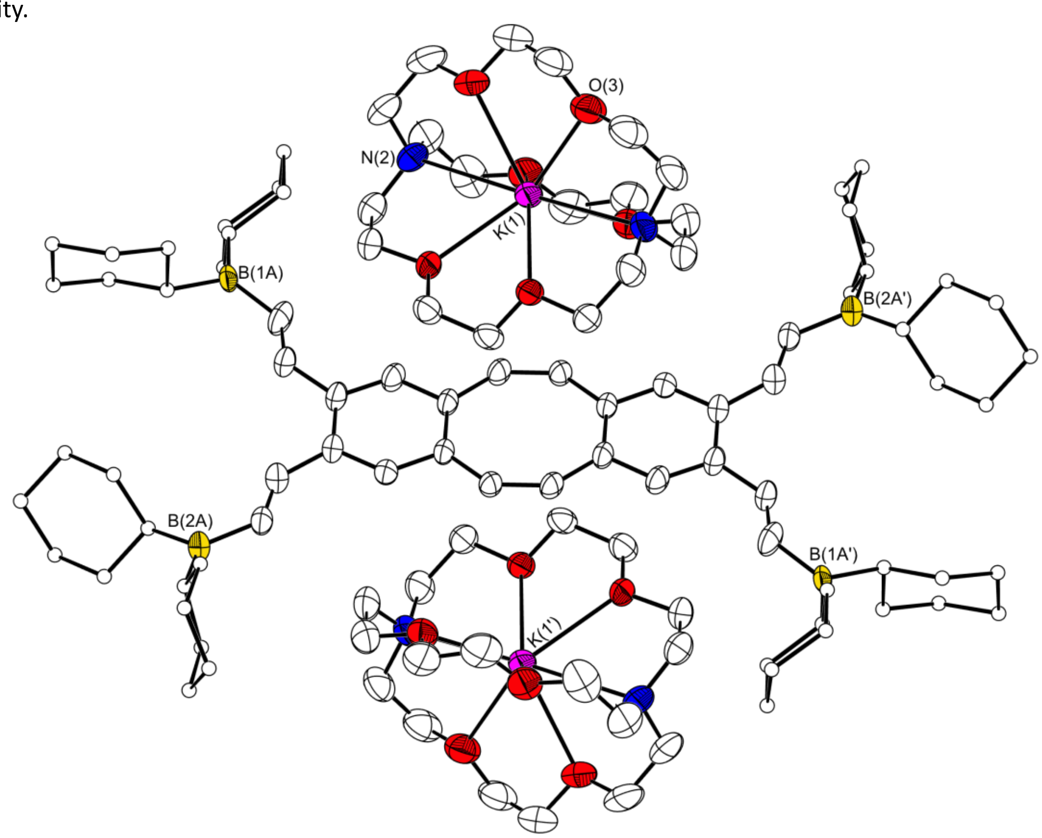

Figure S66. Molecular structure of **1A**^{cry} in the crystalline state. Displacement ellipsoids drawn at the 40% probability level. H atoms are omitted and the carbon atoms of the cyclohexyl groups are only shown as spheres for clarity. Symmetry code: 1–*x*, 1–*y*, 1–*z*.

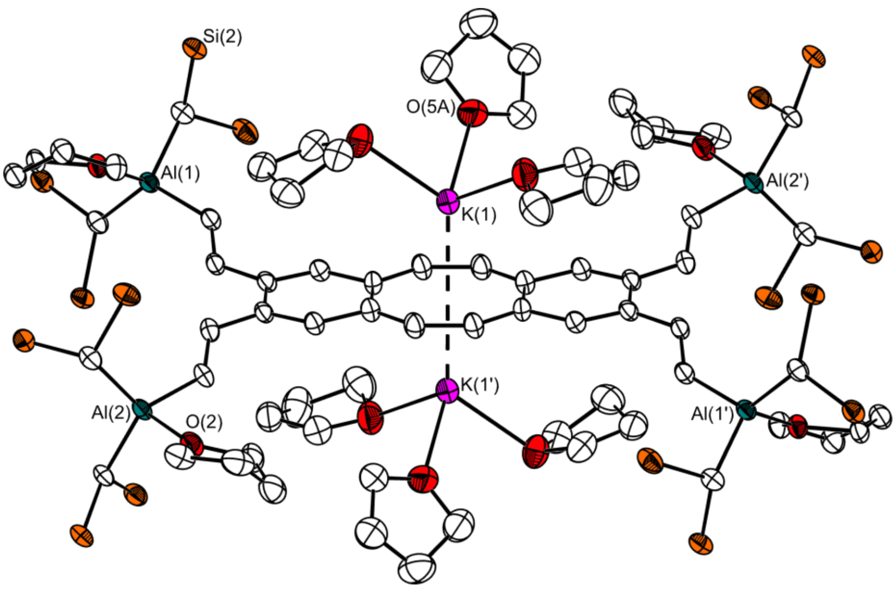

Figure S67. Molecular structure of **2A**^{THF} in the crystalline state. Displacement ellipsoids drawn at the 40% probability level. H atoms and methyl groups are omitted for clarity. Symmetry code: 1–*x*, 1–*y*, 1–*z*.

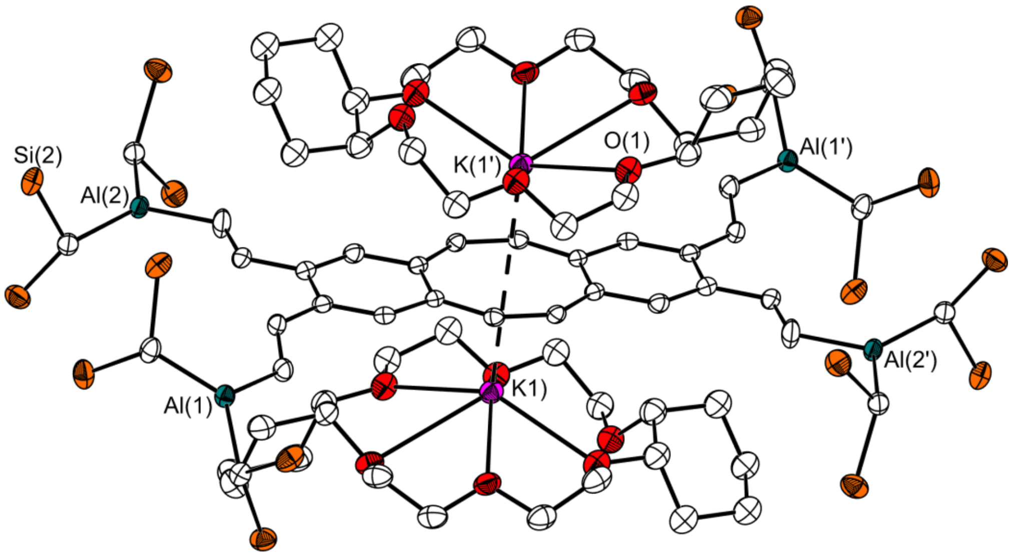

Figure S68. Molecular structure of **2A**^{cro} in the crystalline state. Displacement ellipsoids drawn at the 40% probability level. H atoms and methyl groups are omitted for clarity. Symmetry code: 1–*x*, 1–*y*, 1–*z*.

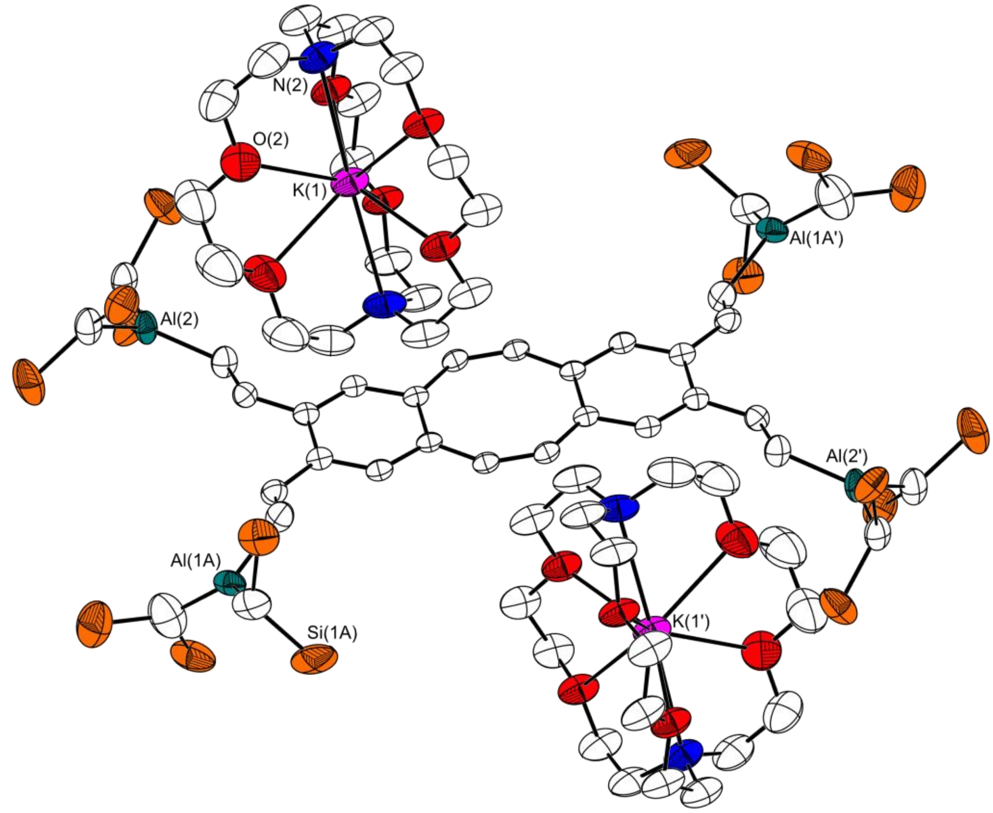

Figure S69. Molecular structure of **2A**^{cry} in the crystalline state. Displacement ellipsoids drawn at the 40% probability level. H atoms and methyl groups are omitted for clarity. Symmetry code: 1–*x*, 1–*y*, 1–*z*.

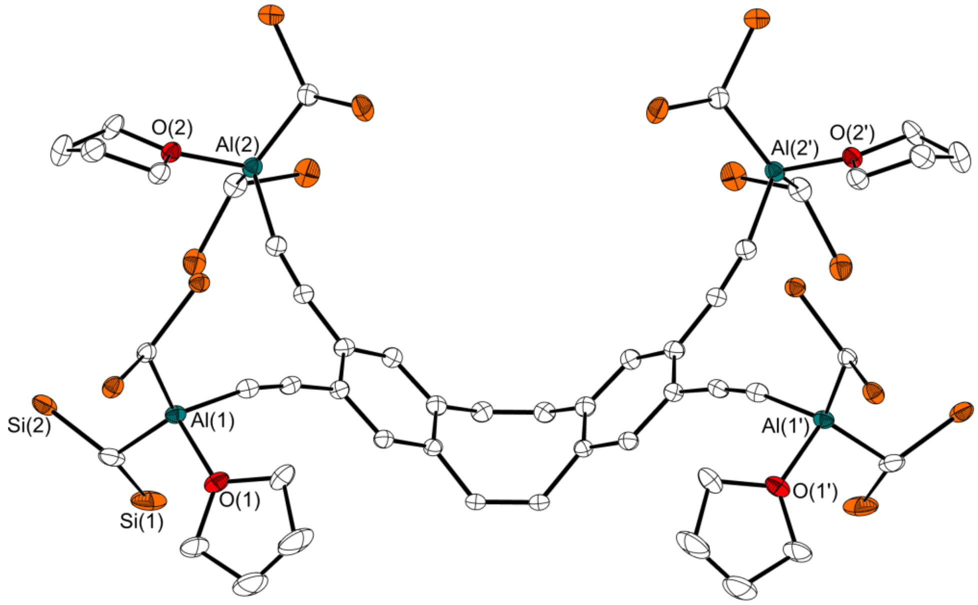

Figure S70. Molecular structure of **3**·4THF in the crystalline state. Displacement ellipsoids drawn at the 40% probability level. H atoms and methyl groups are omitted for clarity. Symmetry code: +*x*, 3/2–*y*, +*z*.

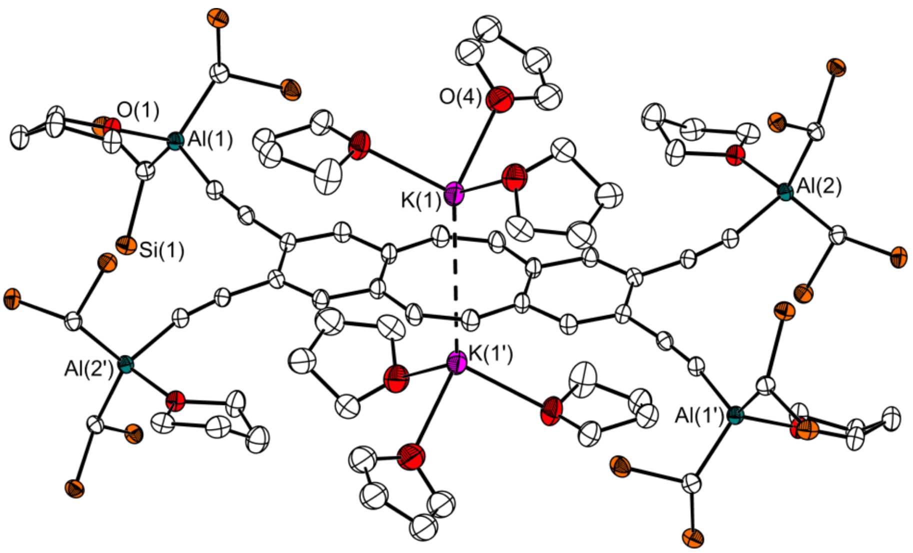

Figure S71. Molecular structure of **3A**^{THF} in the crystalline state. Displacement ellipsoids drawn at the 40% probability level. H atoms and methyl groups are omitted for clarity. Symmetry code: 1–*x*, 1–*y*, 1–*z*.

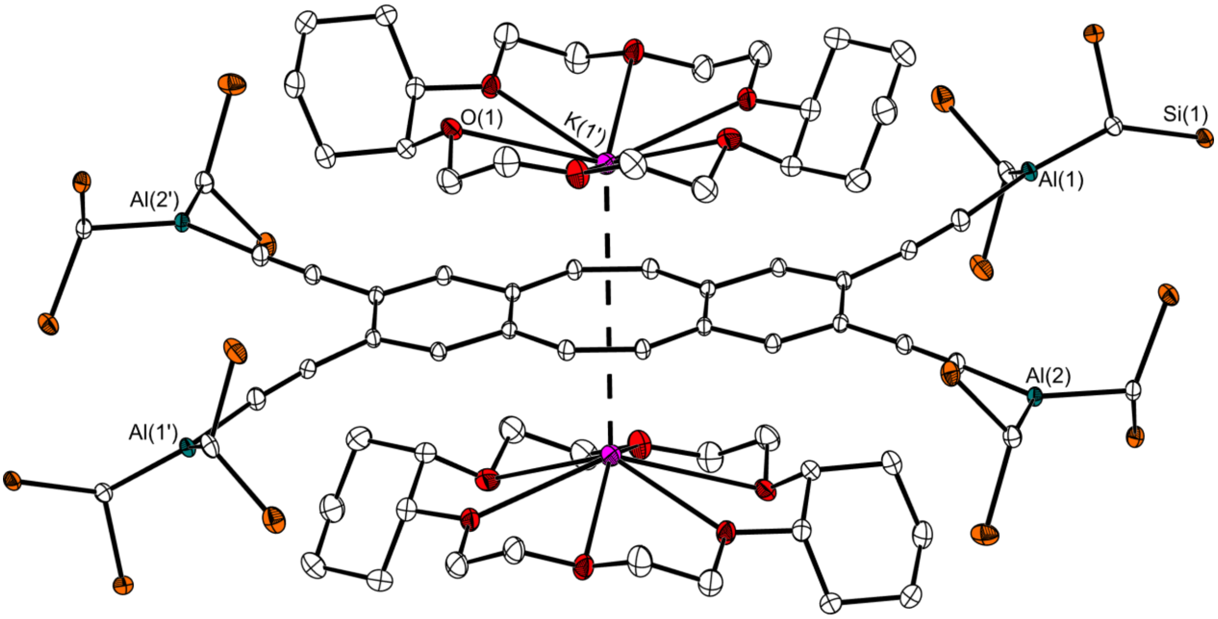

Figure S72. Molecular structure of **3A**^{cro} in the crystalline state. Displacement ellipsoids drawn at the 40% probability level. H atoms and methyl groups are omitted for clarity. Symmetry code: 1–*x*, 1–*y*, 1–*z*.

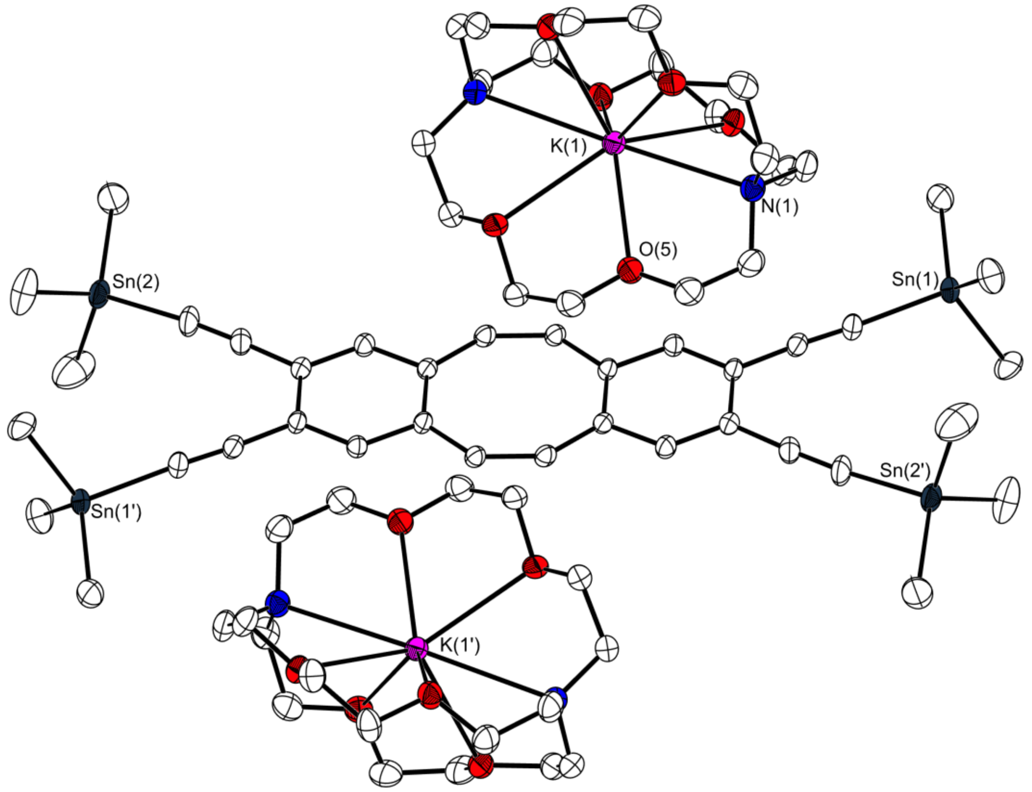

Figure S73. Molecular structure of **4A**^{cry} in the crystalline state. Displacement ellipsoids drawn at the 40% probability level. H atoms are omitted for clarity. Symmetry code: 1–*x*, 1–*y*, 1–*z*.

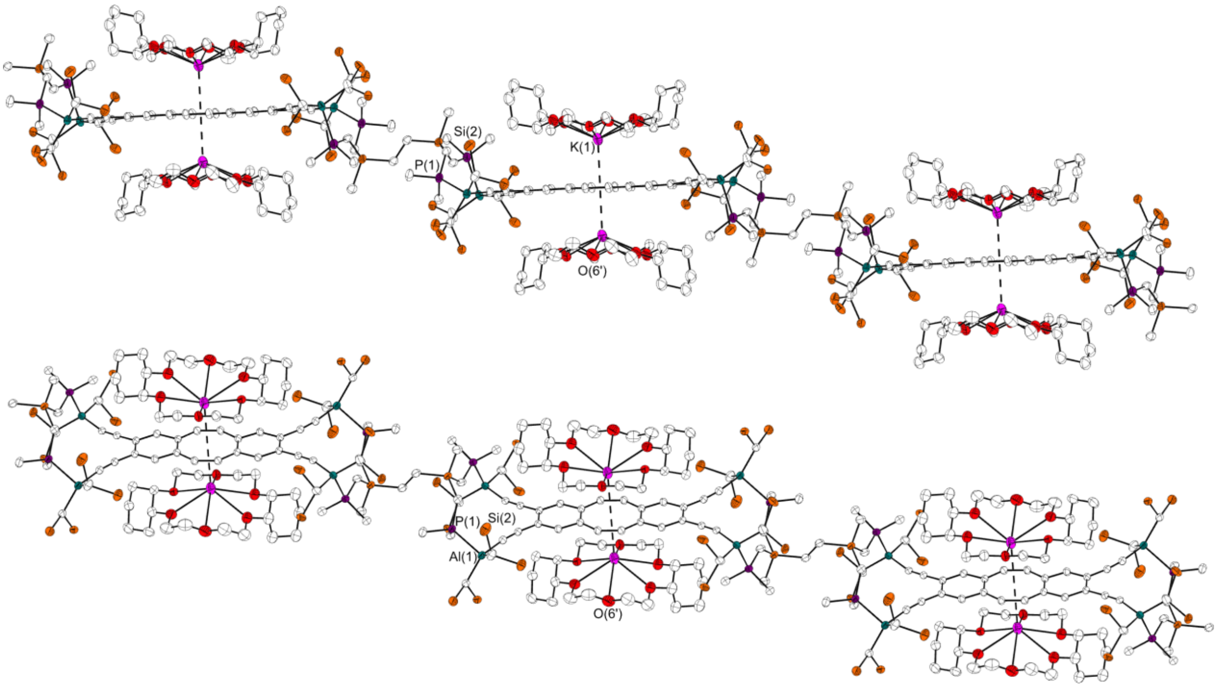

Figure S74. Molecular structure of [$\mathbf{3A}^{cro}\cdot P^4$]$_n$ in the crystalline state. Displacement ellipsoids drawn at the 40% probability level. H atoms are omitted for clarity.

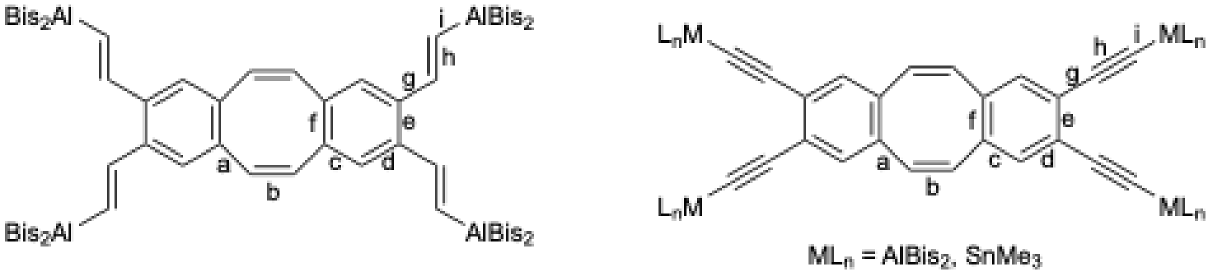

Scheme S2. Naming scheme of the bonds **a**–**i.**

Table S1. Comparison of bond lengths (in Å) **a**–**i** of **2**·4THF[1], $\mathbf{2A}^{THF}$, $\mathbf{2A}^{cro}$, $\mathbf{2A}^{cry}$ and literature data[6,7].

	**2**·4THF[1]	$\mathbf{2A}^{THF}$	$\mathbf{2A}^{cro}$	$\mathbf{2A}^{cry}$	Literature[6,7]
**a**	1.477(3) – 1.482(3)	1.425(4) – 1.427(3)	1.421(3) – 1.424(3)	1.416(4) – 1.425(4)	–
**b**	1.329(5) – 1.334(5)	1.399(4)	1.395(3)	1.386(4)	–
**c**	1.398(4)	1.434(3) – 1.437(4)	1.422(3) – 1.427(3)	1.416(4) – 1.421(4)	–
**d**	1.395(3) – 1.400(3)	1.366(4) – 1.371(3)	1.380(3) – 1.384(3)	1.376(4) – 1.382(4)	–
**e**	1.409(3)	1.450(4)	1.446(3)	1.446(4)	–
**f**	1.400(3)	1.454(4)	1.457(3)	1.458(4)	–
**g**	1.477(3) – 1.482(3)	1.471(4) – 1.478(3)	1.466(3) – 1.468(3)	1.453(4) – 1.458(4)	1.478(2) – 1.488(3)
**h**	1.334(4) – 1.340(4)	1.334(4) – 1.347(4)	1.335(3) – 1.347(3)	1.332(4) – 1.349(4)	1.331(3) – 1.348(3)
**i**	1.981(3) – 1.984(3)	1.969(3) – 1.970(3)	1.922(3) – 1.925(3)	1.834(4) – 1.910(4)	1.941(1) – 1.966(1)

Table S2. Comparison of bond lengths (in Å) **a**–**i** of **3**·4THF, **3A**THF, **3A**cro, and literature data[8] from a neutral system with Ph–CC–AlBis$_2$ units.

	**3**·4THF[a]	**3A**THF	**3A**cro	Literature[8]
**a**	1.465(6) – 1.486(5)	1.424(4) – 1.426(5)	1.418(2) – 1.424(2)	–
**b**	1.326(9) – 1.344(9)	1.398(5)	1.397(2) – 1.400(2)	–
**c**	1.387(6) – 1.408(5)	1.424(4) – 1.427(4)	1.426(2) – 1.430(2)	–
**d**	1.393(5) – 1.409(5)	1.377(4) – 1.379(4)	1.376(2) – 1.383(2)	–
**e**	1.397(5) – 1.411(6)	1.439(5)	1.449(2) – 1.450(2)	–
**f**	1.408(5) – 1.412(6)	1.452(5)	1.459(2) – 1.462(2)	–
**g**	1.432(6) – 1.438(5)	1.435(4) – 1.437(4)	1.426(2) – 1.429(2)	1.435(6) – 1.444(6)
**h**	1.213(6) – 1.225(6)	1.211(5) – 1.213(5)	1.218(2) – 1.221(2)	1.199(6) – 1.211(6)
**i**	1.952(4) – 1.961(5)	1.937(3) – 1.944(3)	1.894(2) – 1.905(2)	1.903(5) – 1.934(4)

[a] **3**·4THF crystallised as a trimer, one of the three molecules showed a high disorder of the AlBis$_2$·THF units, therefore only the two non-disordered molecules were used for the determination of the bond length ranges.

Table S3. Comparison of bond lengths (in Å) **a**–**i** of [**3**·P4]$_n$[1], [**3A**cro·P4]$_n$, **4**[1], and **4A**cry.

	[**3**·P4]$_n$[1]	[**3A**cro·P4]$_n$	**4**[1]	**4A**cry
**a**	1.481(2) – 1.489(2)	1.422(4) – 1.428(4)	1.477(3) – 1.482(3)	1.418(6) – 1.428(6)
**b**	1.329(2) – 1.330(2)	1.401(4)	1.327(3) – 1.335(3)	1.393(6)
**c**	1.388(2) – 1.397(2)	1.434(4)	1.394(3) – 1.402(3)	1.420(6) – 1.424(6)
**d**	1.398(2) – 1.406(2)	1.382(4) – 1.384(4)	1.393(3) – 1.399(3)	1.376(6) – 1.381(6)
**e**	1.415(2) – 1.416(2)	1.444(4)	1.411(3) – 1.414(3)	1.442(7)
**f**	1.400(3) – 1.404(3)	1.447(4)	1.402(3) – 1.406(3)	1.460(6)
**g**	1.438(2) – 1.442(2)	1.434(4) – 1.439(4)	1.433(3) – 1.440(3)	1.424(6) – 1.426(6)
**h**	1.208(2) – 1.216(3)	1.219(5) – 1.223(4)	1.202(3) – 1.203(3)	1.202(7) – 1.207(7)
**i**	1.963(2) – 1.973(2)	1.952(3) – 1.955(3)	2.104(2) – 2.117(2)	2.074(5) – 2.081(5)
**Al–P**	2.497(1) – 2.518(7)	2.481(1) – 2.524(2)	–	–

Table S4. Crystal data for $\mathbf{1A}^{THF}$, $\mathbf{1A}^{cry}$, $\mathbf{2A}^{THF}$, $\mathbf{2A}^{cro}$ and $\mathbf{2A}^{cry}$.

	$\mathbf{1A}^{THF,[a]}$	$\mathbf{1A}^{cry,[b]}$	$\mathbf{2A}^{THF,[c]}$	$\mathbf{2A}^{cro,[d]}$	$\mathbf{2A}^{cry,[e]}$
Empirical formula	$C_{108}H_{176}B_4K_2O_9$	$C_{124}H_{208}B_4K_2N_4O_{16}$	$C_{128}H_{264}Al_4K_2O_{12}Si_{16}$	$C_{132}H_{252}Al_4K_2O_{12}Si_{16}$	$C_{164}H_{288}Al_4K_2N_4O_{12}Si_{16}$
M [g mol^{-1}]	1739.92	2132.37	2630.94	2666.88	3143.53
T [K]	100.0(1)	100.0(1)	100.0(1)	100.0(1)	100.0(1)
Crystal system	triclinic	triclinic	triclinic	triclinic	triclinic
Space group	$P\bar{1}$	$P\bar{1}$	$P\bar{1}$	$P\bar{1}$	$P\bar{1}$
a [Å]	9.7829(2)	11.8509(3)	12.3918(3)	13.2700(3)	13.6570(2)
b [Å]	18.3119(3)	15.0517(4)	15.3295(3)	14.6642(4)	17.7758(3)
c [Å]	29.7951(3)	18.4022(5)	23.2875(5)	22.1940(5)	22.9274(4)
α [°]	84.310(1)	74.334(2)	105.607(2)	91.114(2)	96.225(1)
β [°]	84.165(1)	80.554(2)	102.626(2)	94.9453(19)	106.003(1)
γ [°]	77.325(2)	85.952(2)	100.482(2)	110.693(3)	110.513(2)
V [Å^3]	5164.28(15)	3116.53(15)	4018.86(16)	4019.39(19)	4879.32(15)
Z	2	1	1	1	1
ρ_{calc} [g cm^{-3}]	1.119	1.136	1.087	1.102	1.070
μ [mm^{-1}]	1.221	1.149	2.253	0.250	1.936
F(000) [e]	1908	1166	1442	1454	1710
Size [mm]	0.40 × 0.25 × 0.16	0.19 × 0.11 × 0.05	0.46 × 0.16 × 0.14	0.12 × 0.1 × 0.06	0.27 × 0.19 × 0.12
λ [Å]	1.54184 (Cu Kα)	1.54184 (Cu Kα)	1.54184 (Cu Kα)	0.71073 (Mo Kα)	1.54184 (Cu Kα)
2θ range [°]	4.962 to 152.818	6.100 to 152.026	6.192 to 152.284	6.598 to 54.206	5.452 to 133.198
hkl range	$-11 \le h \le 12$, $-22 \le k \le 22$, $-37 \le l \le 37$	$-14 \le h \le 14$, $-17 \le k \le 18$, $-23 \le l \le 22$	$-15 \le h \le 15$, $-19 \le k \le 17$, $-29 \le l \le 29$	$-17 \le h \le 17$, $-18 \le k \le 18$, $-28 \le l \le 28$	$-16 \le h \le 16$, $-21 \le k \le 21$, $-27 \le l \le 27$
Ref. collected	84814	46843	86303	180267	98781
Independent ref.	21298	12660	16579	17716	17241
$R_{int.}$ / R_{sigma}	0.0469 / 0.0334	0.0546 / 0.0452	0.1116 / 0.0630	0.0868 / 0.0496	0.0350 / 0.0204
Refl. with $I > 2\sigma(I)$.	18471	9293	13603	12633	13669
Data/restraints/param.	21298/360/1156	12660/366/795	16579/223/782	17716/607/863	17241/1213/1020
GoF on F^2	1.035	1.041	1.043	1.054	1.083
R_1 / ωR_2 [I>$2\sigma(I)$]	0.0507 / 0.1353	0.0539 / 0.1456	0.0655 / 0.1730	0.0582 / 0.1319	0.0928 / 0.2670
R_{int} (all data) /ωR_2	0.0575 / 0.1409	0.0725 / 0.1589	0.0772 / 0.1847	0.0918 / 0.1505	0.1075 / 0.2844
ρ_{fin} (max/min) [e Å^{-3}]	0.39/−0.32	0.38/−0.33	0.63/−0.91	0.56/−0.49	1.08/−0.58
CCDC	2441691	2441692	2441693	2441694	2441695

[a] A solvent mask was calculated and 236 electrons were found in a volume of 868 Å^3 in 5 voids per unit cell. This is consistent with the presence of 3 THF per asymmetric unit which account for 240 electrons per unit cell. Disorder of one THF molecule (O2; C77-C80) over two sites; ratio: 50:50. Disorder of one THF molecule (O3; C81-C84) over two sites; ratio: 76 (labelled "A"):24 (labelled "B"). Disorder of one THF molecule (O4; C85-C88) over two sites; ratio: 64 (labelled "A"):36 (labelled "B"). Partial disorder of one cyclohexyl unit (C26-C30) over two sites; ratio: 64 (labelled "A"):36 (labelled "B").
[b] Disorder of one BCy_2 group (B1; C11-22) over two sites; ratio 63 (labelled "A"):37 (labelled "B"). Disorder of one $CHBCy_2$ group (B2; C24-36) over two sites; ratio 80 (labelled "A"):20 (labelled "B"). A solvent mask was calculated and 157 electrons were found in a volume of 570 Å^3 in 1 void per unit cell. This is consistent with the presence of 4 THF per asymmetric unit which account for 160 electrons per unit cell. [c] Disorder of one THF molecule (O5; C57-60) over two sites; ratio 51 (labelled "A"):49 (labelled "B"). Partial disorder of one THF molecule (C50/51) over two sites; ratio 51 (labelled "A"):49 (labelled "B"). [d] Disorder over two sites (90:10) of O2, O3, C41 to C45, C47, C48 and C50. Necessary restraints and constraints were applied. [e] Disorder of one Bis_2Al-group (Al1; Si1-4; C11-24) and one bis(trimethylsilyl)methyl group (Si5/6; C27-33) over two sites; ratio 63 (labelled "A"):37 (labelled "B"). Partial disorder of one bis(trimethylsilyl)methyl group (Si7/8; C35-40) over two sites; ratio 79 (labelled "A"):21 (labelled "B"). A solvent mask was calculated and 326 electrons were found in a volume of 1290 Å^3 in 2 voids per unit cell. This is consistent with the presence of 6[C_6H_6], 2[C_6H_6] per asymmetric unit which account for 336 electrons per unit cell.

Table S5. Crystal data for **3**·4THF, **3A**^{THF}, **3A**^{cro}, [**3A**^{cro}·P4]$_n$ and **4A**^{cry}.

	**3**·4THF[a]	**3A**$^{THF,[b]}$	**3A**$^{cro,[c]}$	[**3A**^{cro}·P4]$_n$[d]	**4A**^{cry}
Empirical formula	$C_{344}H_{688}Al_{12}O_{26}Si_{48}$	$C_{140}H_{280}Al_4K_2O_{15}Si_{16}$	$C_{150}H_{262}Al_4K_2O_{12}Si_{16}$	$C_{172}H_{308}Al_4K_2O_{12}P_4Si_{18}$	$C_{80}H_{132}K_2N_4O_{14}Sn_4$
M [g mol^{-1}]	6912.98	2839.18	2893.14	3383.79	1926.85
T [K]	100.0(1)	100.0(1)	100.0(1)	100.0(1)	100.0(1)
Crystal system	orthorhombic	triclinic	triclinic	triclinic	monoclinic
Space group	*Pnma*	$P\bar{1}$	$P\bar{1}$	$P\bar{1}$	$P2_1/c$
a [Å]	16.6817(1)	11.7371(4)	13.50242(15)	14.0404(4)	19.7550(2)
b [Å]	42.3038(2)	15.6216(5)	22.0476(3)	19.5580(5)	18.8964(3)
c [Å]	62.4883(4)	24.9219(6)	32.9014(3)	20.2218(7)	12.3794(2)
α [°]	90	76.564(2)	107.1574(9)	75.597(3)	90
β [°]	90	77.400(3)	97.2907(9)	70.974(3)	93.023(1)
γ [°]	90	76.935(3)	98.0718(10)	85.188(2)	90
V [Å^3]	44098.0(4)	4262.7(2)	9118.18(18)	5084.6(3)	4614.78(11)
Z	4	1	2	1	2
ρ_{calc} [g cm^{-3}]	1.041	1.106	1.054	1.105	1.387
μ [mm^{-1}]	1.891	2.171	0.225	2.281	9.770
F(000) [e]	15152	1554	3144	1838	1980
Size [mm]	0.59 × 0.28 × 0.21	0.29 × 0.08 × 0.07	0.37 × 0.32 × 0.24	0.20 × 0.12 × 0.08	0.17 × 0.10 × 0.04
λ [Å]	1.54184 (Cu Kα)	1.54184 (Cu Kα)	0.71073 (Mo Kα)	1.54184 (Cu Kα)	1.54184 (Cu Kα)
2θ range [°]	4.728 to 140.15	5.918 to 133.188	6.392 to 60.162	5.828 to 152.622	6.478 to 152.916
hkl range	$-20 \le h \le 20$, $-51 \le k \le 51$, $-74 \le l \le 76$	$-13 \le h \le 13$, $-15 \le k \le 18$, $-29 \le l \le 29$	$-19 \le h \le 19$, $-31 \le k \le 31$, $-46 \le l \le 46$	$-17 \le h \le 16$, $-24 \le k \le 21$, $-25 \le l \le 24$	$-24 \le h \le 24$, $-21 \le k \le 23$, $-15 \le l \le 15$
Ref. collected	988627	65223	315748	63012	129109
Independent ref.	42478	15047	53420	20877	9575
$R_{int.}$ / R_{sigma}	0.0809 / 0.0217	0.1052 / 0.0800	0.0528 / 0.0453	0.0569 / 0.0606	0.0732 / 0.0297
Refl. with $I > 2\sigma(I)$.	38482	11065	39519	15611	8273
Data/restraints/param.	42478/2964/2248	15047/153/838	53420/1161/1659	20877/1097/1125	9575/0/476
GoF on F^2	1.070	1.034	1.028	1.049	1.075
R_1 / ωR_2 [I>2$\sigma(I)$]	0.1013 / 0.2447	0.0639 / 0.1642	0.0464 / 0.1058	0.0729 / 0.1871	0.0493 / 0.1305
R_{int} (all data) /ωR_2	0.1069 / 0.2484	0.0872 / 0.1893	0.0680 / 0.1148	0.0927 / 0.2032	0.0584 /0.1371
ρ_{fin} (max/min) [e Å^{-3}]	0.79/−0.90	0.76/−0.62	0.62/−0.53	0.81/−0.79	1.30/−1.75
CCDC	2441696	2441697	2441698	2441699	2441700

[a] Disorder of THF and Bis-groups of one complex. THF molecules are disordered over two sites in ratio 57:43 and 51:49. One Bis-group is disordered over three sites in ratio 0.38: 0.29:0.33, the other is disordered over two sites in ratio 0.64:0.36. Distances and displacement parameters were restrained. A solvent mask was calculated and 2352 electrons were found in a volume of 10514 Å^3 in 3 voids per unit cell. This is consistent with the presence of 2 THF, 12 THF per asymmetric unit which account for 2240 electrons per unit cell.

[b] Disorder of C50 over two sites; ratio: 77 (labelled "A"):23 (labelled "B"). Disorder of C53-C55 over two sites; ratio: 59 (labelled "A"):41 (labelled "B"). Disorder of C57 and C60 over two sites; ratio: 70 (labelled "A"):30 (labelled "B"). One solvent molecule (THF) is disordered and partially occupied. Therefore a solvent mask was calculated and 37 electrons were found in a volume of 186 Å^3 in 2 voids per unit cell. This is consistent with the presence of 1 THF per formula unit which account for 40 electrons per unit cell. [c] Disorder of C_6H_6 trough the lattice. The solvent molecules were removed and a solvent mask was calculated and 434 electrons were found in a volume of 2212 Å^3 in 2 voids per unit cell. This is consistent with the presence of 5[C_6H_6] per asymmetric unit which account for 420 electrons per unit cell. The bigger part of one crown ether is disordered over two sites in ratio 57:43. Also one $SiMe_3$ group is disordered over two sites in ratio 59:41. Necessary restraints were applied. [d] Partial disorder of one bis(tri-methylsilyl)methyl group (C27/31-33) and disorder of one benzene molecule (C75-80) over two sites; ratio 57 (labelled "A"):43 (labelled "B"). Partial disorder of the tetraphosphane species (Si9; C44-46), disorder of one cyclohexyl unit (C59-64) and disorder of one benzene molecule (C69-74) over two sites; ratio 83 (labelled "A"):17 (labelled "B"). A solvent mask was calculated and 84 electrons were found in a volume of 530 Å^3 in one void per unit cell. This is consistent with the presence of two benzene molecules per asymmetric unit which account for 84 electrons per unit cell.

Quantum Chemical Calculations

Molecular structures of **1** (R = -C(H)=C(H)-BCy_2), **1**$^{2-}$ (dianion), **3** (R = -CC-$AlBis_2$) and **3**$^{2-}$ (dianion) in free state (modelled as isolated molecules) have been optimised at the PBEh-3c level of theory.[9] For these and further calculations the Orca 6.0.1 software package[10] has been used, unless otherwise noted. As the starting approximation, the solid-state structures have been taken when available, otherwise they were manually constructed. The target convergence in geometry optimizations has been set to *TightOpt* level. In cases of small deviations from higher symmetry, the optimised structures were additionally symmetrized. Also in all Orca calculations we used the settings *TightSCF* and *DefGrid3*, as well as the RIJCOSX accelerating approximation[11] whenever possible. Depending on the molecule, several types of electronic structure have been tested, closed shell singlet (abbreviated further as CS, using restricted Kohn-Sham formalism RKS), open shell singlet (OS, broken-symmetry unrestricted Kohn-Sham, UKS), and triplet (T, UKS). All results are summarised in Table S6. The optimised molecular structures corresponding to the most stable electronic solutions are shown in Figures S75–S78. Spin density plot for the most stable OS electronic solution of **1**$^{2-}$ is shown in Figure S79.

Fractional occupation weighted density (FOD)[12] calculations at the PBE0/def2-TZVP (T_{el} = 10000 K) level of theory[13,14] were performed for all molecules, see Table S6 for the resulting N_{FOD} values. The FOD plots (Figures S80 – S83) show spatial distributions of the so-called "hot" electrons.

TD-DFT calculation for **1**$^{2-}$ was performed at the PBE0/def2-TZVP level of theory using the TDA approximation and CPCM solution model for the THF solvent. The number of calculated roots was restricted to 40. Selected transitions from this calculation are listed in Table S7. Frontier molecular orbitals are collected in Table S8. Simulated UV-Vis spectrum is shown in Figures S84.

Natural bond orbital (NBO) analyses[15] have been performed using NBO 7.0.10 program.[16] The required wave-functions have been taken from single-point PBE0/def2-TZVP calculations using the optimised most stable structures. Note, for **1**$^{2-}$ the OS electronic solution has been used. The natural charges and Wiberg bond indices for selected atoms and atom pairs are collected in Table S9.

From the same wave-functions electron densities were calculated and processed for obtaining electron density difference plots. The procedure was as follows. An electron density has been calculated for neutral **1** taking the geometry optimised for **1**$^{2-}$. The obtained density has been subtracted from the electron density of **1**$^{2-}$. Thus, the difference density has been obtained and plotted, see Figure S85. Analogously the difference density has been calculated for the pair **3** – **3**$^{2-}$, see Figure S86.

Calculations for complexes of **1**, **1**$^{2-}$, **3** and **3**$^{2-}$ with PMe_3 have been done at the PBEh-3c level of theory. Geometry optimizations were performed for closed shell singlets using the same settings as described above. To each molecule four PMe_3 species were coordinated, one per Lewis acid function B or Al. All complexes appeared to be theoretically stable. Using the minimised electronic energies the energies have been calculated for reactions (PLA is one of **1**, **1**$^{2-}$, **3** and **3**$^{2-}$):

$$\text{PLA} + 4\ \text{PMe}_3 \rightarrow \text{PLA}\cdot 4\text{PMe}_3 \qquad \Delta E$$

The obtained ΔE values were (in kcal/mol) −89.7, −33.9, −97.2 and −68.6 for **1**, **1**$^{2-}$, **3** and **3**$^{2-}$, respectively.

Table S6. Results[a] of the quantum-chemical calculations

Mol.	E_{DFT}	$<S^2>_{DFT}$	N_{FOD}
**1**	0 (CS), 45.6 (T)	2.086 (T)	1.93
$\mathbf{1}^{2-}$	1.3 (CS), 0 (OS), 5.3 (T)	0.622 (OS), 2.044 (T)	3.48
**3**	0 (CS), 60.5 (T)	2.104 (T)	2.33
$\mathbf{3}^{2-}$	0 (CS), 12.2 (T)	2.031 (T)	3.88

[a] E_{DFT} are relative adiabatic energies in kcal/mol obtained from DFT calculations for different electronic solutions (CS closed shell singlet, OS broken-symmetry open-shell singlet, T triplet) indicated in parentheses; $<S^2>_{DFT}$ are expectation values of the spin-squared operator for the respective electronic solutions; N_{FOD} shows the number of "hot" electrons obtained as the result of FOD calculation.

Table S7. Most important transitions (vertical energy differences) in the TD-DFT calculation of $\mathbf{1}^{2-}$. Wavelengths λ in nm, oscillator strengths f via transition electric dipole moments and assignments are listed.

λ	f	Assignment
844	2.61	91 % HOMO → LUMO
439	3.92	91 % HOMO-1 → LUMO+1

Table S8. Frontier molecular orbitals (isosurfaces 0.05 a.u.) and respective energies (eV) in TD-DFT calculation of $\mathbf{1}^{2-}$.

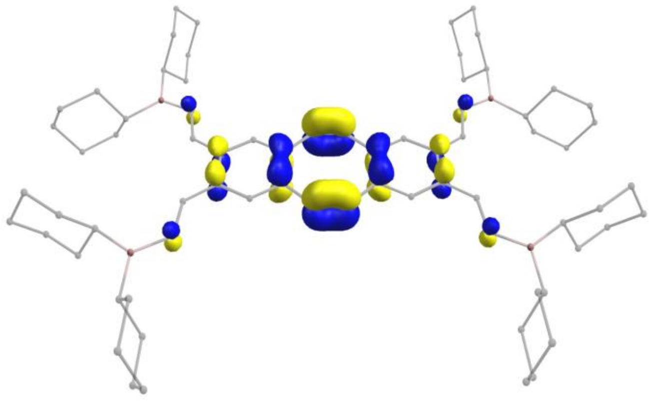 HOMO-1 (−4.3147)	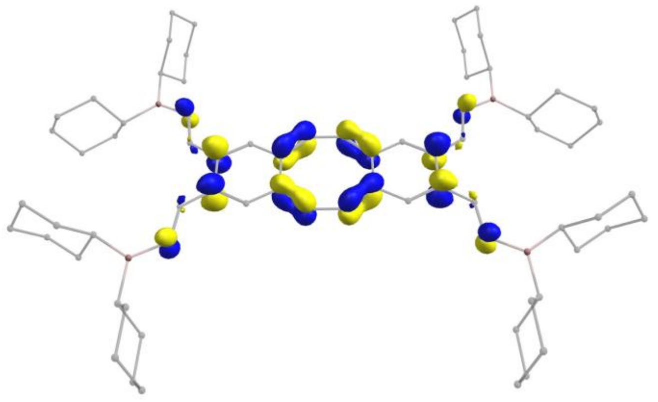 HOMO (−2.8634)
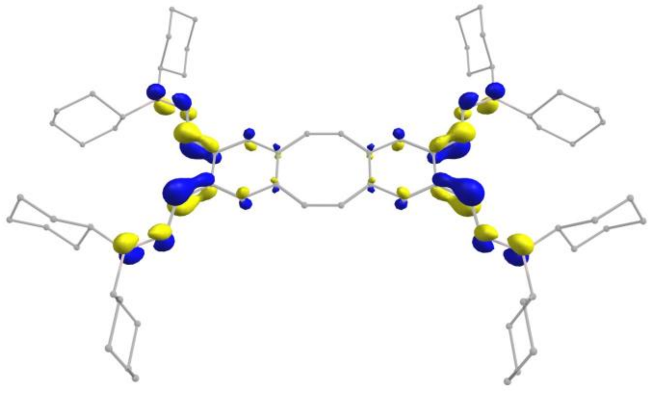 LUMO (−1.4428)	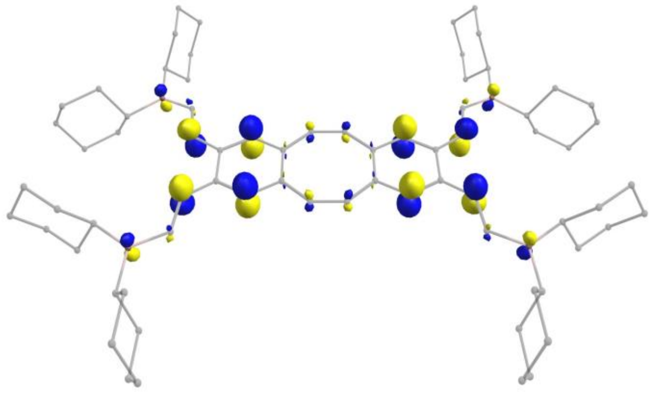 LUMO+1 (−0.9677)

Table S9. Results of NBO calculations. Natural charges and Wiberg bond indices are given for single atoms and atomic pairs, respectively. Natural spin densities are given in parentheses.

Atom or Atom Pair	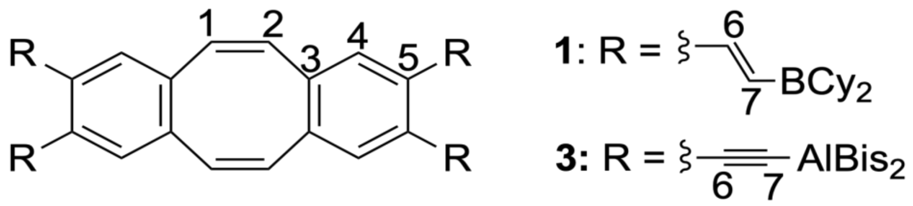			
	**1**	$\mathbf{1}^{2-}$	**3**	$\mathbf{1}^{2-}$
C1	−0.18	−0.22 (0.057)	−0.18	−0.24
C2	−0.18	−0.22 (-0.057)	−0.18	−0.24
C3	−0.05	−0.09 (0.043)	−0.05	−0.10
C4	−0.16	−0.11 (-0.035)	−0.15	−0.10
C5	−0.06	−0.14 (0.045)	−0.10	−0.21
C6	−0.10	−0.08 (0.035)	−0.01	0.09
C7	−0.57	−0.66 (0.046)	−0.64	−0.84
B/Al	0.99	0.80 (0.074)	1.99	1.89
C1-C2 (b)	1.89	1.59	1.90	1.51
C2-C3 (a)	1.05	1.21	1.04	1.26
C3-C4 (c)	1.40	1.42	1.40	1.37
C4-C5 (d)	1.39	1.34	1.38	1.38
C5-C6 (g)	1.09	1.26	1.11	1.21
C6-C7 (h)	1.80	1.55	2.77	2.61
C7-B/C7-Al (i)	0.94	1.14	0.50	0.62

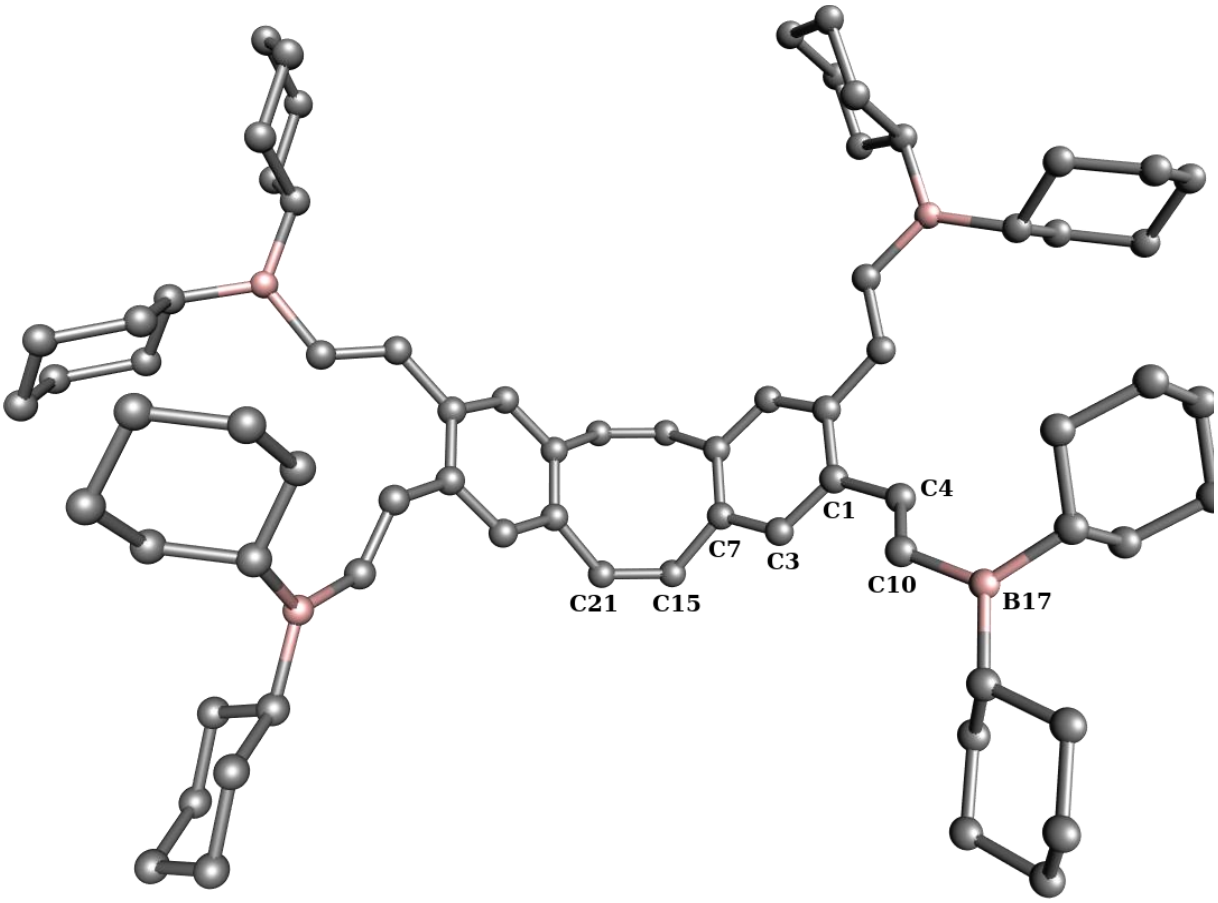

Figure S75. Optimised molecular structure of free **1** (C_1 symmetry). Hydrogen atoms are omitted for clarity. The internal working numbering of the selected atoms is shown. Selected equilibrium parameters (Å, degrees) are: *r*(C3-C1)=1.390, *r*(C4-C1)=1.465, *r*(C7-C3)=1.390, *r*(C10-C4)=1.342, *r*(C15-C7)=1.472, *r*(B17-C10)=1.557, *r*(C21-C15)=1.330, *a*(C3-C1-C4)=120.0, *a*(C1-C3-C7)=122.9, *a*(C1-C4-C10)=125.8, *a*(C3-C7-C15)=117.9, *a*(C4-C10-B17)=125.1, *a*(C7-C15-C21)=126.9, *t*(C7-C3-C1-C4)=177.5, *t*(C3-C1-C4-C10)=−30.5, *t*(C1-C3-C7-C15)=177.8, *t*(C1-C4-C10-B17)=172.5, *t*(C3-C7-C15-C21)=124.0.

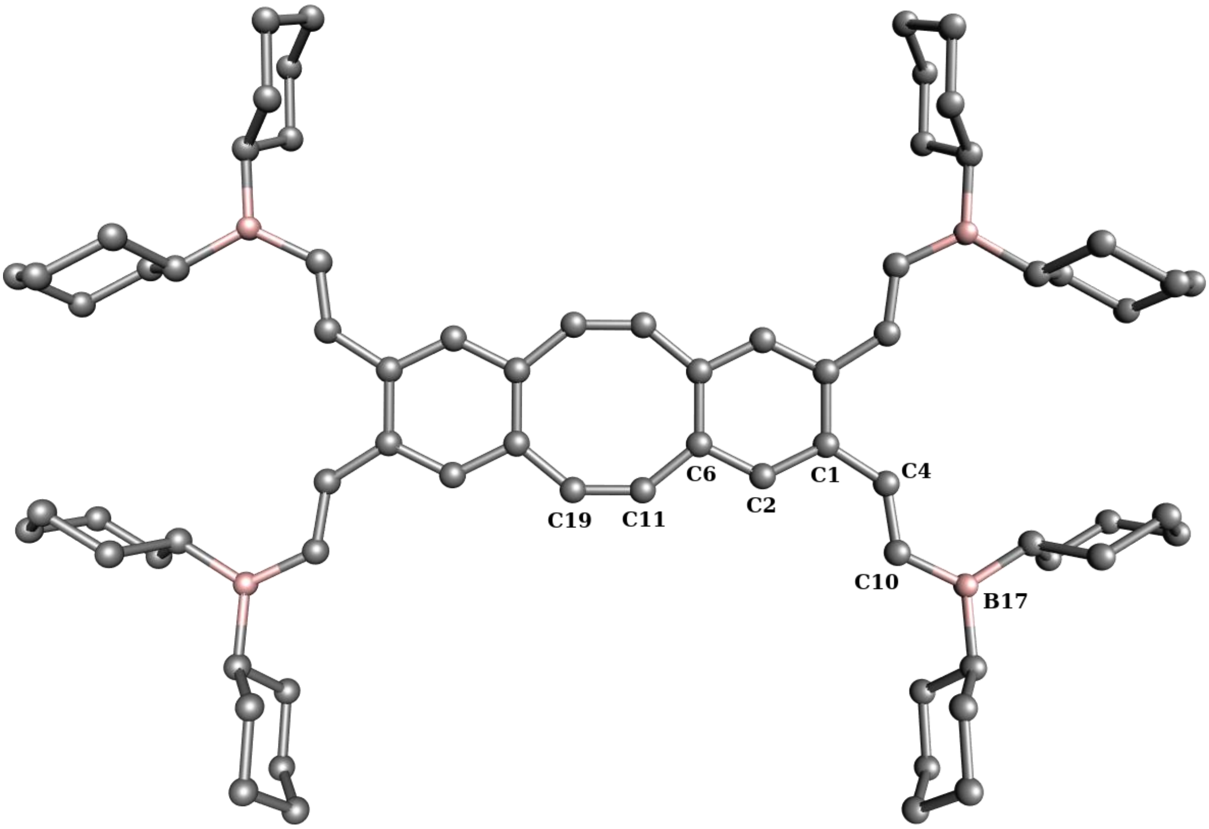

Figure S76. Optimised molecular structure of free $\mathbf{1}^{2-}$ (C_{2h} symmetry). Hydrogen atoms are omitted for clarity. The internal working numbering of the selected atoms is shown. Selected equilibrium parameters (Å, degrees) are: *r*(C2-C1)=1.397, *r*(C4-C1)=1.422, *r*(C6-C2)=1.393, *r*(C10-C4)=1.377, *r*(C11-C6)=1.433, *r*(B17-C10)=1.510, *r*(C19-C11)=1.363, *a*(C2-C1-C4)=120.8, *a*(C1-C2-C6)=126.7, *a*(C1-C4-C10)=130.8, *a*(C2-C6-C11)=113.8, *a*(C4-C10-B17)=123.7, *a*(C6-C11-C19)=140.5, *t*(C6-C2-C1-C4)=–177.6, *t*(C2-C1-C4-C10)=-6.3, *t*(C1-C2-C6-C11)=179.5, *t*(C1-C4-C10-B17)=–179.5, *t*(C2-C6-C11-C19)=179.9.

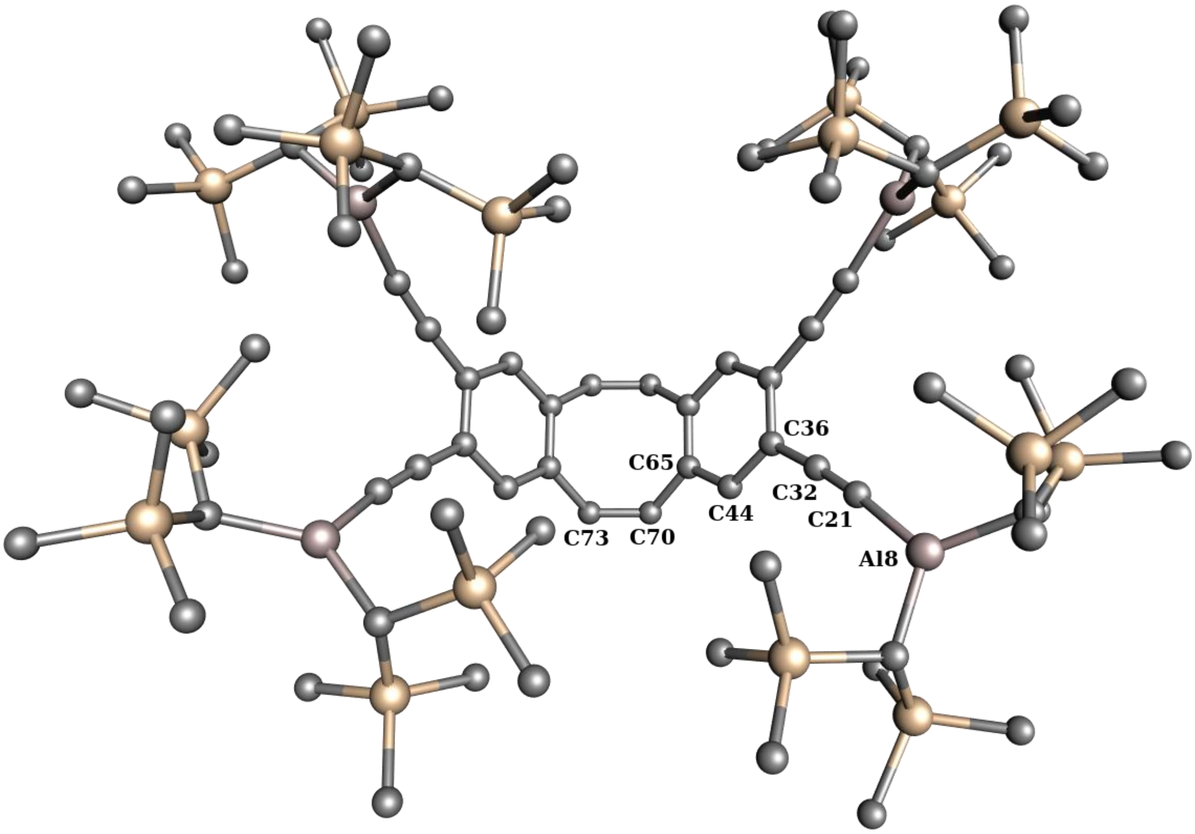

Figure S77. Optimised molecular structure of free **3** (C_1 symmetry). Hydrogen atoms are omitted for clarity. The internal working numbering of the selected atoms is shown. Selected equilibrium parameters (Å, degrees) are: *r*(C21-Al8)=1.915, *r*(C32-C21)=1.217, *r*(C36-C32)=1.419, *r*(C44-C36)=1.392, *r*(C65-C44)=1.389, *r*(C70-C65)=1.474, *r*(C73-C70)=1.329, *a*(Al8-C21-C32)=173.0, *a*(C21-C32-C36)=175.7, *a*(C32-C36-C44)=118.9, *a*(C36-C44-C65)=122.2, *a*(C44-C65-C70)=118.4, *a*(C65-C70-C73)=124.7, *t*(Al8-C21-C32-C36)=25.1, *t*(C21-C32-C36-C44)=0.7, *t*(C32-C36-C44-C65)=177.8, *t*(C36-C44-C65-C70)=−179.3, *t*(C44-C65-C70-C73)=117.2.

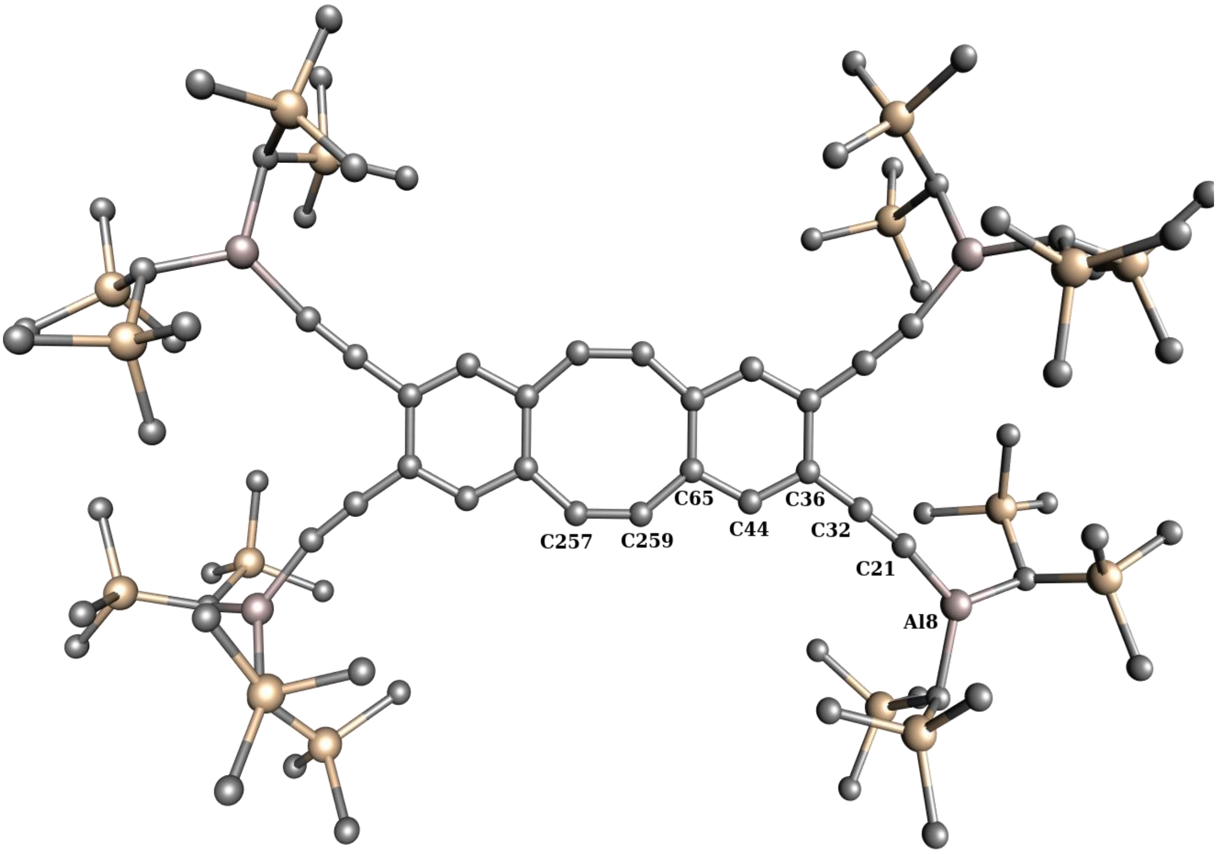

Figure S78. Optimised molecular structure of free **3**$^{2-}$ (C_1 symmetry). Hydrogen atoms are omitted for clarity. The internal working numbering of the selected atoms is shown. Selected equilibrium parameters (Å, degrees) are: *r*(C21-Al8)=1.862, *r*(C32-C21)=1.231, *r*(C36-C32)=1.396, *r*(C44-C36)=1.387, *r*(C65-C44)=1.405, *r*(C259-C65)=1.418, *r*(C259-C257)=1.376, *a*(Al8-C21-C32)=168.9, *a*(C21-C32-C36)=176.3, *a*(C32-C36-C44)=119.0, *a*(C36-C44-C65)=126.3, *a*(C44-C65-C259)=113.6, *a*(C65-C259-C257)=140.1, *t*(Al8-C21-C32-C36)=13.9, *t*(C21-C32-C36-C44)=30.0, *t*(C32-C36-C44-C65)=−175.9, *t*(C36-C44-C65-C259)=180.0, *t*(C44-C65-C259-C257)=−179.8.

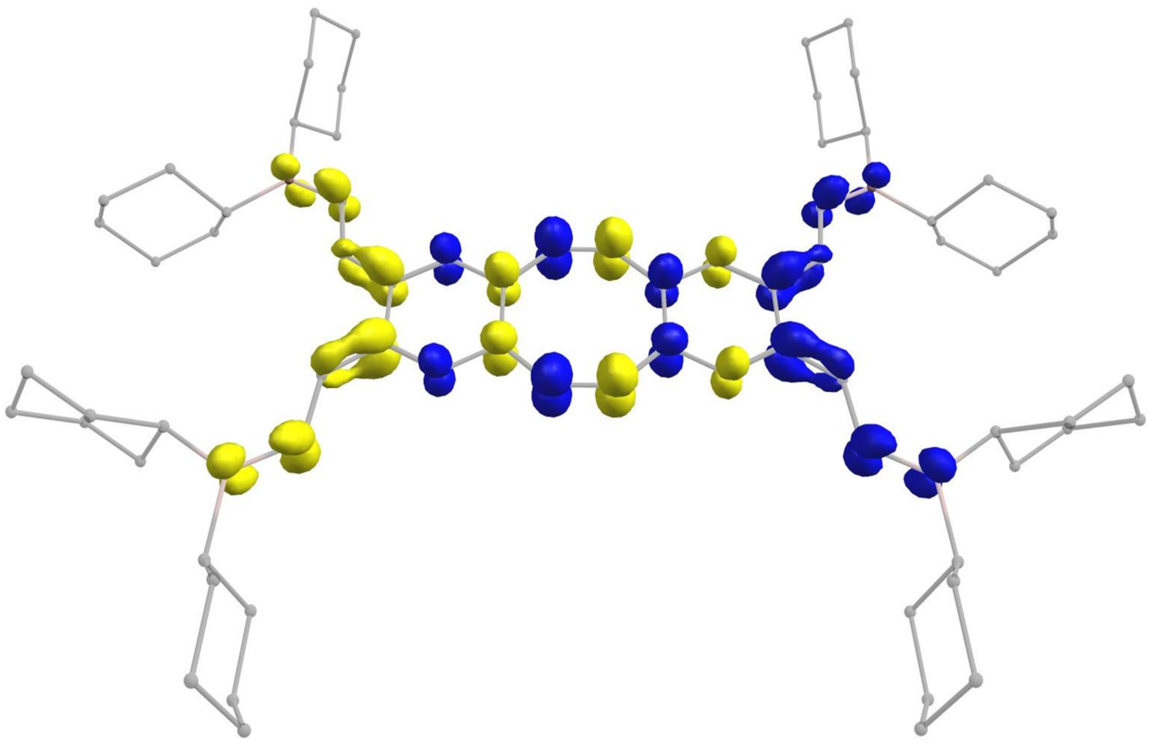

Figure S79. Spin density (isosurfaces 0.005 a.u.) of **1**$^{2-}$. Hydrogen atoms are omitted for clarity.

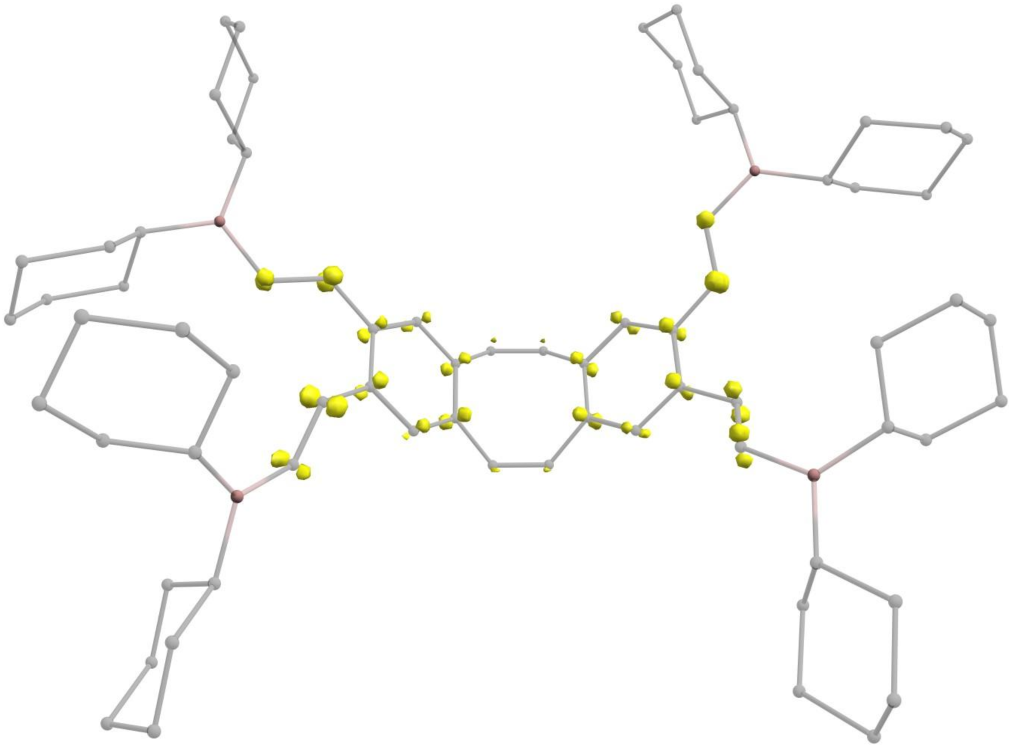

Figure S80. FOD plot (isosurfaces 0.005 a.u. in yellow) of **1**. Hydrogen atoms are omitted for clarity.

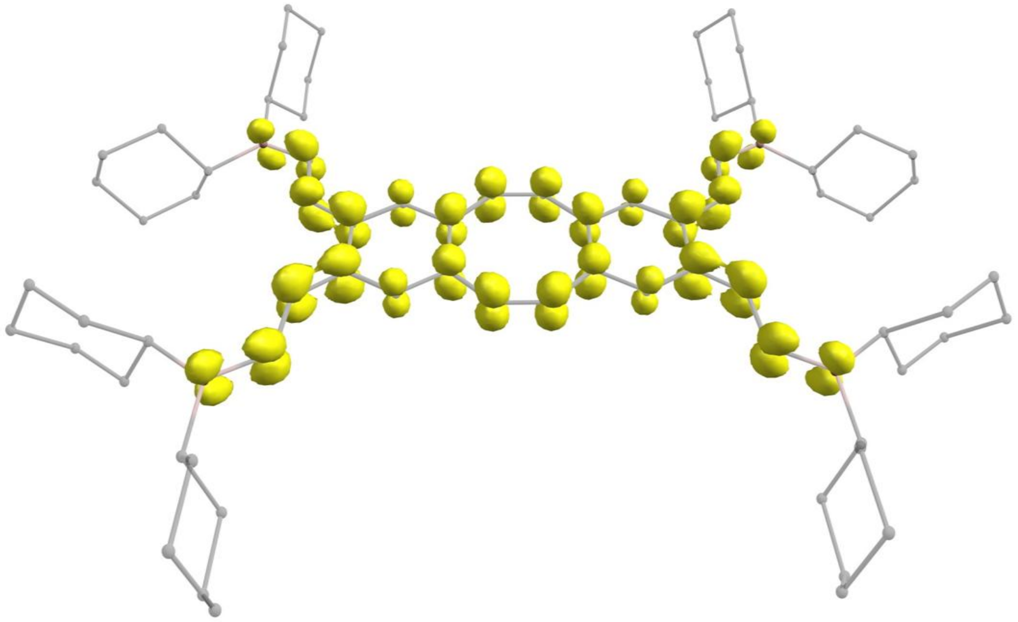

Figure S81. FOD plot (isosurfaces 0.005 a.u. in yellow) of **1**$^{2-}$. Hydrogen atoms are omitted for clarity.

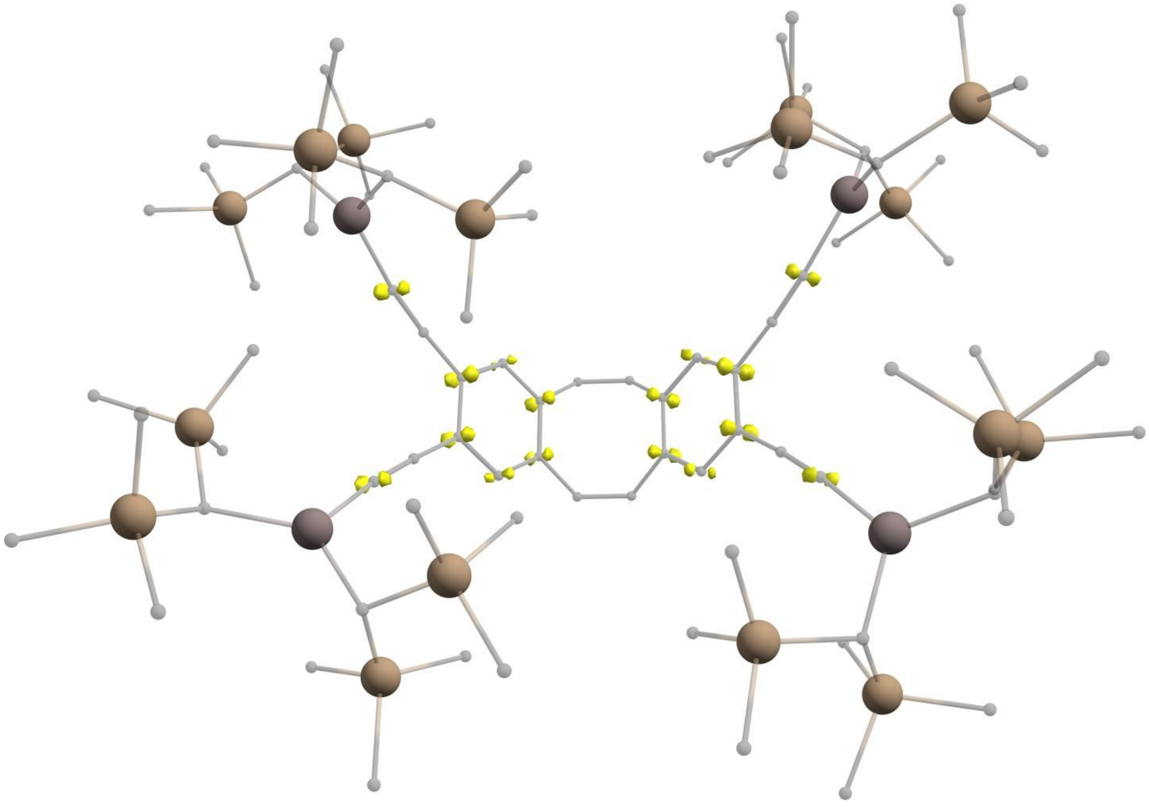

Figure S82. FOD plot (isosurfaces 0.005 a.u. in yellow) of **3**. Hydrogen atoms are omitted for clarity.

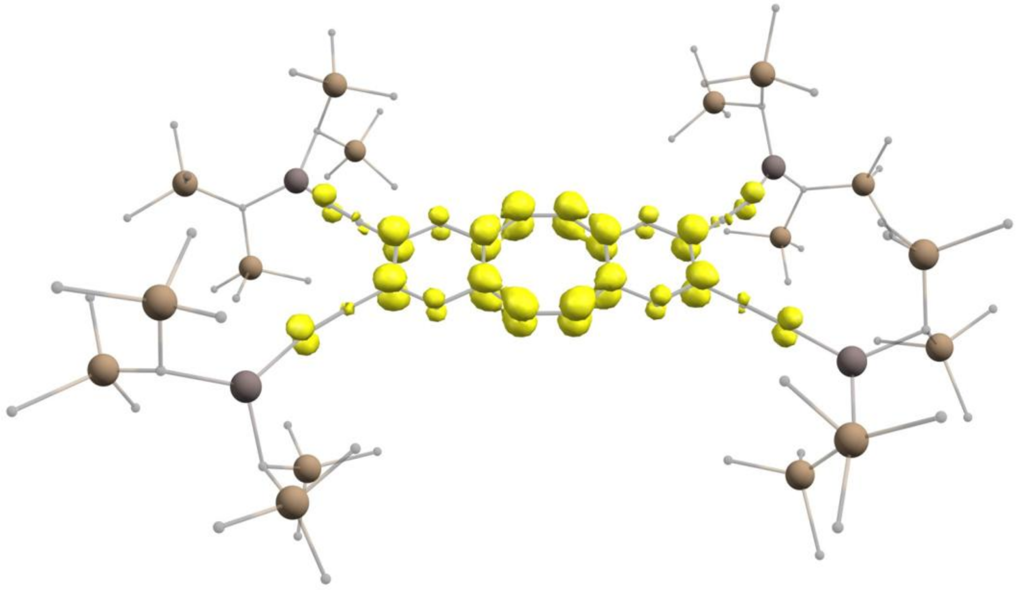

Figure S83. FOD plot (isosurfaces 0.005 a.u. in yellow) of **3**$^{2-}$. Hydrogen atoms are omitted for clarity.

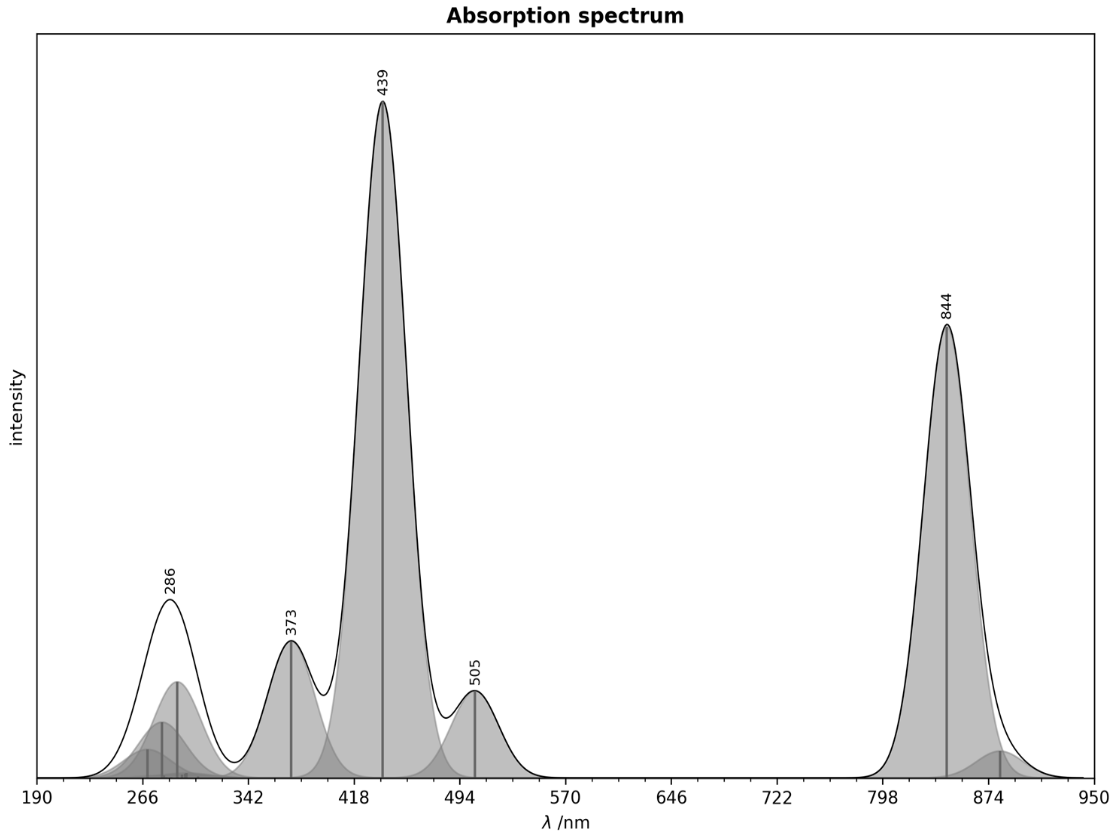

Figure S84. Simulated UV-Vis spectrum of **1**$^{2-}$ based on TD-DFT calculation. Individual transitions are shown as vertical sticks and are approximated as Gaussian functions (grey areas with 20 nm FWHM). Full line is the sum of all Gaussians. No empirical shift has been applied to the transition energies.

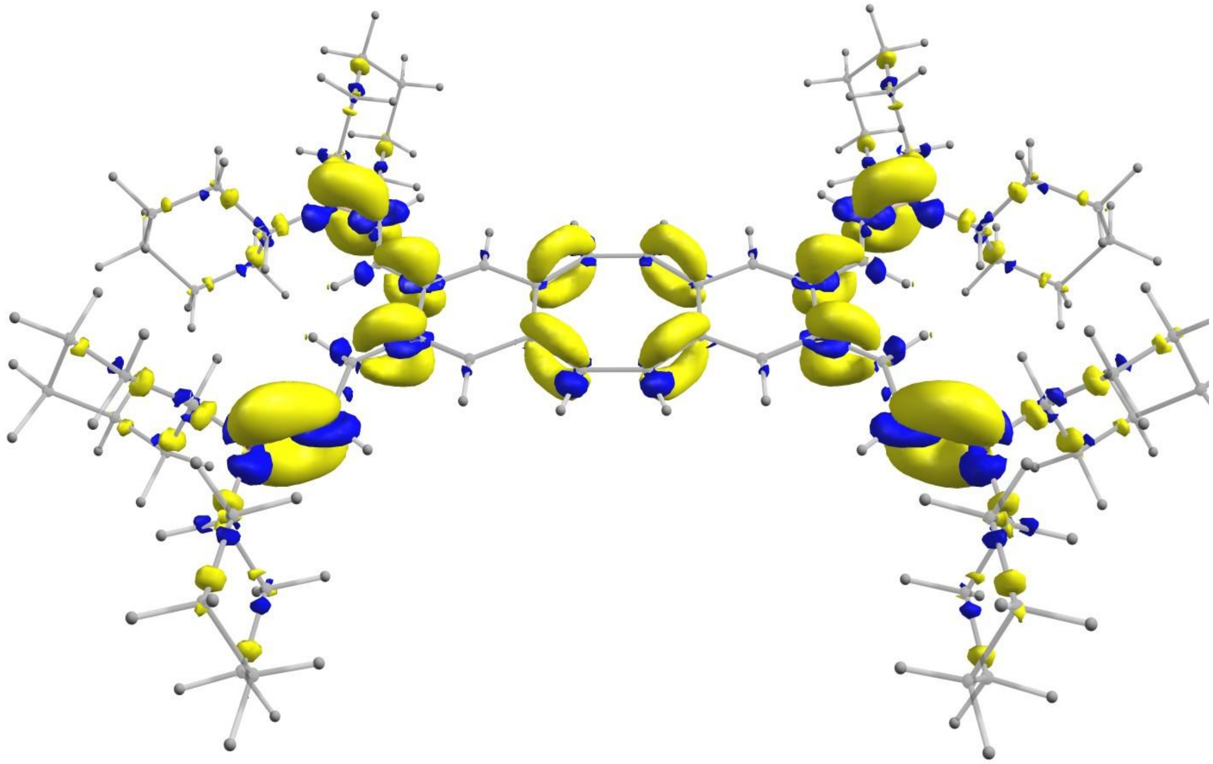

Figure S85. Electron density difference (isosurfaces for ±0.003 a.u.) for the pair **1** – **1**$^{2-}$. Yellow surfaces correspond to the positive value (0.003 a.u., more electron density in **1**$^{2-}$), blue surfaces correspond to negative value (−0.003 a.u., more electron density in **1**).

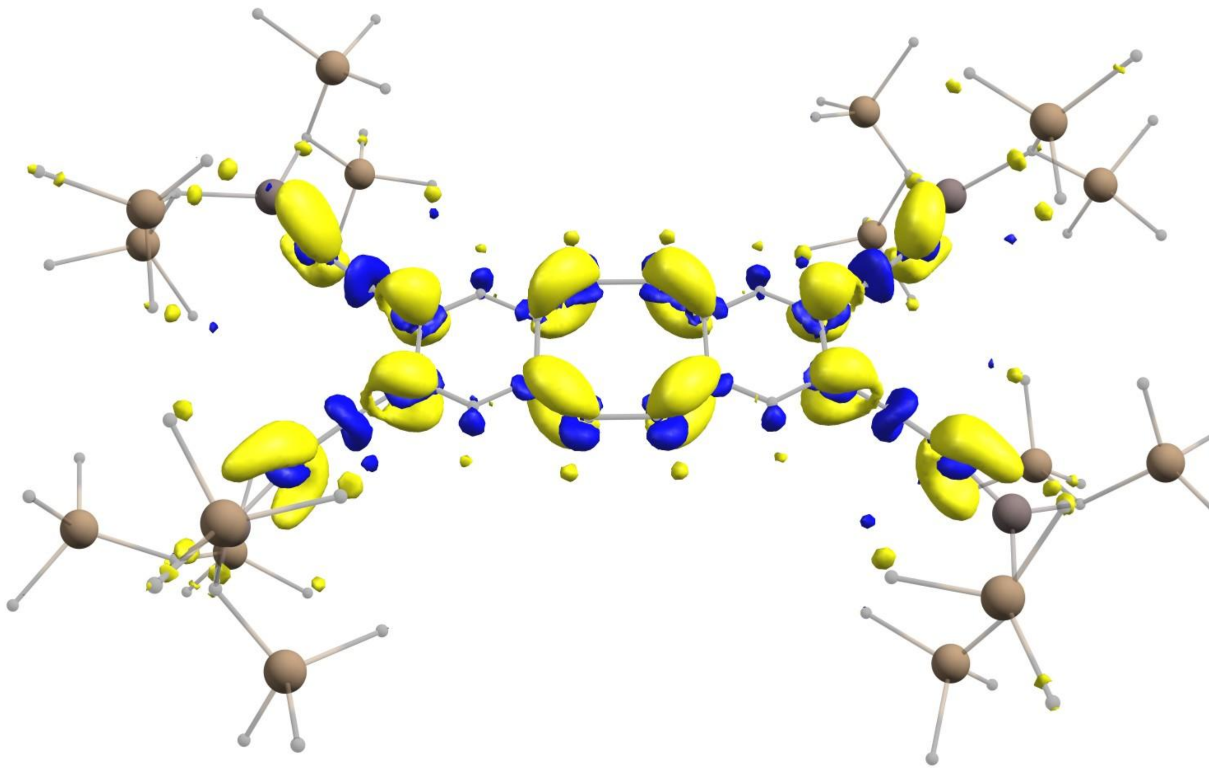

Figure S86. Electron density difference (isosurfaces for ±0.003 a.u.) for the pair **3** – **3**$^{2-}$. Yellow surfaces correspond to the positive value (0.003 a.u., more electron density in **3**$^{2-}$), blue surfaces correspond to negative value (−0.003 a.u., more electron density in **3**).

References

[1] M. J. Klingsiek, J. Buth, P. C. Trapp, A. Mix, J.-H. Lamm, B. Neumann, G. Stammler, N. W. Mitzel, *Inorg. Chem. Front.* **2025**, 10.1039.D5QI00823A.

[2] O. V. Dolomanov, L. J. Bourhis, R. J. Gildea, J. A. K. Howard, H. Puschmann, *J. Appl. Crystallogr.* **2009**, *42*, 339–341.

[3] G. M. Sheldrick, *Acta Crystallogr. Sect. C* **2015**, *71*, 3–8.

[4] G. M. Sheldrick, *Acta Crystallogr. Sect. A* **2008**, *64*, 112–122.

[5] L. J. Bourhis, O. V. Dolomanov, R. J. Gildea, J. A. K. Howard, H. Puschmann, *Acta Crystallogr. Sect. Found. Adv.* **2015**, *71*, 59–75.

[6] J. Rudlof, N. Aders, J.-H. Lamm, B. Neumann, H.-G. Stammler, N. W. Mitzel, *ChemistryOpen* **2021**, *10*, 1020–1027.

[7] N. Aders, P. C. Trapp, J.-H. Lamm, J. L. Beckmann, B. Neumann, H.-G. Stammler, N. W. Mitzel, *Organometallics* **2022**, *41*, 3600–3611.

[8] M. Franke, M. J. Klingsiek, J. Buth, J.-H. Lamm, B. Neumann, H.-G. Stammler, N. W. Mitzel, *Dalton Trans.* **2024**, *53*, 7751–7762.

[9] S. Grimme, J. G. Brandenburg, C. Bannwarth, A. Hansen, *J. Chem. Phys.* **2015**, *143*, 054107.

[10] F. Neese, *WIREs Comput. Mol. Sci.* **2022**, *12*, e1606.

[11] F. Neese, F. Wennmohs, A. Hansen, U. Becker, *Chem. Phys.* **2009**, *356*, 98–109.

[12] C. A. Bauer, A. Hansen, S. Grimme, *Chem. – Eur. J.* **2017**, *23*, 6150–6164.

[13] F. Weigend, R. Ahlrichs, *Phys. Chem. Chem. Phys.* **2005**, *7*, 3297.

[14] C. Adamo, V. Barone, *J. Chem. Phys.* **1999**, *110*, 6158–6170.

[15] F. Weinhold, *Valency and Bonding: A Natural Bond Orbital Donor-Acceptor Perspective*, Cambridge University Press, Cambridge, UK, **2005**.

[16] NBO 7.0. E. D. Glendening, J. K. Badenhoop, A. E. Reed, J. E. Carpenter, J. A. Bohmann, C. M. Morales, P. Karafiloglou, C. R. Landis, and F. Weinhold, Theoretical Chemistry Institute, University of Wisconsin, Madison, WI **2018**.
